# gcMeta: a Global Catalogue of Metagenomics platform to support the archiving, standardization and analysis of microbiome data

**DOI:** 10.1093/nar/gky1008

**Published:** 2018-10-26

**Authors:** Wenyu Shi, Heyuan Qi, Qinglan Sun, Guomei Fan, Shuangjiang Liu, Jun Wang, Baoli Zhu, Hongwei Liu, Fangqing Zhao, Xiaochen Wang, Xiaoxuan Hu, Wei Li, Jia Liu, Ye Tian, Linhuan Wu, Juncai Ma

**Affiliations:** 1 Microbial Resource and Big Data Center, Institute of Microbiology, Chinese Academy of Sciences, Beijing 100101, China; 2 State Key Laboratory of Microbial Resources, Institute of Microbiology, Chinese Academy of Sciences, Beijing 100101, China; 3 CAS Key Laboratory of Pathogenic Microbiology and Immunology, Institute of Microbiology, Chinese Academy of Science, Beijing 100101, China; 4 University of Chinese Academy of Sciences, Beijing 100049, China; 5 Collaborative Innovation Centre for Diagnosis and Treatment of Infectious Diseases First Attainted Hospital, College of Medicine, Zhejiang University, Hangzhou 310058, China; 6 Beijing Key Laboratory of Antimicrobial Resistance and Pathogen Genomics, Beijing 100101, China; 7 State Key Laboratory of Mycology, Institute of Microbiology, Chinese Academy of Science, Beijing 100101, China; 8 Computational Genomics Lab, Beijing Institutes of Life Science, Chinese Academy of Sciences, Beijing 100101, China; 9 Internet of Things Information Technology and Application Laboratory, Computer Network Information Center, Chinese Academy of Sciences. Beijing 100101, China

## Abstract

Meta-omics approaches have been increasingly used to study the structure and function of the microbial communities. A variety of large-scale collaborative projects are being conducted to encompass samples from diverse environments and habitats. This change has resulted in enormous demands for long-term data maintenance and capacity for data analysis. The Global Catalogue of Metagenomics (gcMeta) is a part of the ‘Chinese Academy of Sciences Initiative of Microbiome (CAS-CMI)’, which focuses on studying the human and environmental microbiome, establishing depositories of samples, strains and data, as well as promoting international collaboration. To accommodate and rationally organize massive datasets derived from several thousands of human and environmental microbiome samples, gcMeta features a database management system for archiving and publishing data in a standardized way. Another main feature is the integration of more than ninety web-based data analysis tools and workflows through a Docker platform which enables data analysis by using various operating systems. This platform has been rapidly expanding, and now hosts data from the CAS-CMI and a number of other ongoing research projects. In conclusion, this platform presents a powerful and user-friendly service to support worldwide collaborative efforts in the field of meta-omics research. This platform is freely accessible at https://gcmeta.wdcm.org/.

## INTRODUCTION

‘Meta-omics’ (e.g. metataxonomics, metagenomics and metatranscriptomics) approaches have been increasingly used to study the structure, function and intercellular interactions of the microbial communities and the fundamental mechanisms of microbial life and evolution. Dramatic progress in the next generation sequencing technology has made large-scale sampling and sequencing possible, even for individual laboratory. Meta-omics has also promoted collaborative efforts in a grand vision across the international research community, as exemplified by the Earth Microbiome Project (EMP) (1) and Human Microbiome Project (HMP) (2). These collaborative projects have produced large volume of data and hence generated meaningful interpretations from a full spectrum of sources which are impossible with a single independent study. Along with these changes, microbiome research is becoming a data driven science (3) and rapid advances in this area have brought about significant challenges. Firstly, comparing data from independent research groups becomes difficult if standard operating procedures (SOPs) and reporting standards are not followed. Adhering to universal standards in every step of the study, including sampling, sequencing, data submission, data analysis, and data publication, is necessary to understand the results of a single study within a broader context (4). Recently, significant efforts have been made for the development of universal protocols and standards (5). Although, well-organized and reputable collaborative projects often have built-in standards (http://www.microbiome-standards.org/) and SOPs (6), sometimes it becomes difficult for different projects to implement identical standards. For example, human and environmental microbiota data from different projects are not readily comparable due to inconsistencies in standards, protocols as well as workflows (7). Further developments, and more importantly, adoption of these SOPs and standards by all projects and labs worldwide is crucial for the scientific community. The second challenge is long-term preservation and open access of the data. Integration of all relevant publicly available data is a prerequisite for future cross-studies. A stable and robust data infrastructure is needed that would provide a reliable data archive and rational data organization, thus ensuring data reproducibility and allowing data reinterpretation. The third challenge is to analyze Gigabyte (GB) to Terabyte (TB) scale data on a single computer. Despite the availability of a variety of stand-alone tools, it is almost impossible for any given individual lab to have sufficient infrastructure for data storage and to maintain multi-computer network clusters resources (8). Currently, there are several public resources, including the European Bioinformatics Institute (EBI) metagenomics (9), the Metagenomics RAST server (MG-RAST) (10) and the JGI IMG Integrated Microbial Genomes & Microbiomes (IMG/M) (11). However, considering the rapid increase in data volume and growing demands for data analysis capacity, more public services with the ability to provide data archiving and cloud-based data analysis are required (12).

Because of the wide geographical coverage, rich ecosystem, as well as diverse ethnicity and lifestyles of the people, China harbors enormous diversity of microbial communities. In comparison to developed countries, however, the microbial communities in China are less comprehensively and systematically studied. China also lacks nationwide collaborative projects. The Chinese Academy of Sciences Initiative of Microbiome (CAS-CMI) is one of the leading projects organized at the national level to find solutions to the current challenges in human and environmental health, agriculture and industrial developments. At the same time, it will establish the biobanks (samples, strains and data) of Chinese Microbiome Initiative, and support long term preservation and reuse of data worldwide in a free and open way.

The Global Catalogue of Metagenomics (gcMeta) platform is a part of the CAS-CMI. As a partner database of the World Data Center for Microorganisms (WDCM) (13) as well, gcMeta has two features: firstly, designing and implementing as a standardized and state-of-art database management system to support long-term preservation and integration data from the CAS-CMI project as well as from microbiome research projects worldwide. Secondly, the platform provides web-based tools and pre-defined workflows, along with computing resources for massive data analysis requirements globally.

## PLATFORM DESIGN AND IMPLEMENTATION

### How to use the platform

**Figure 1.**
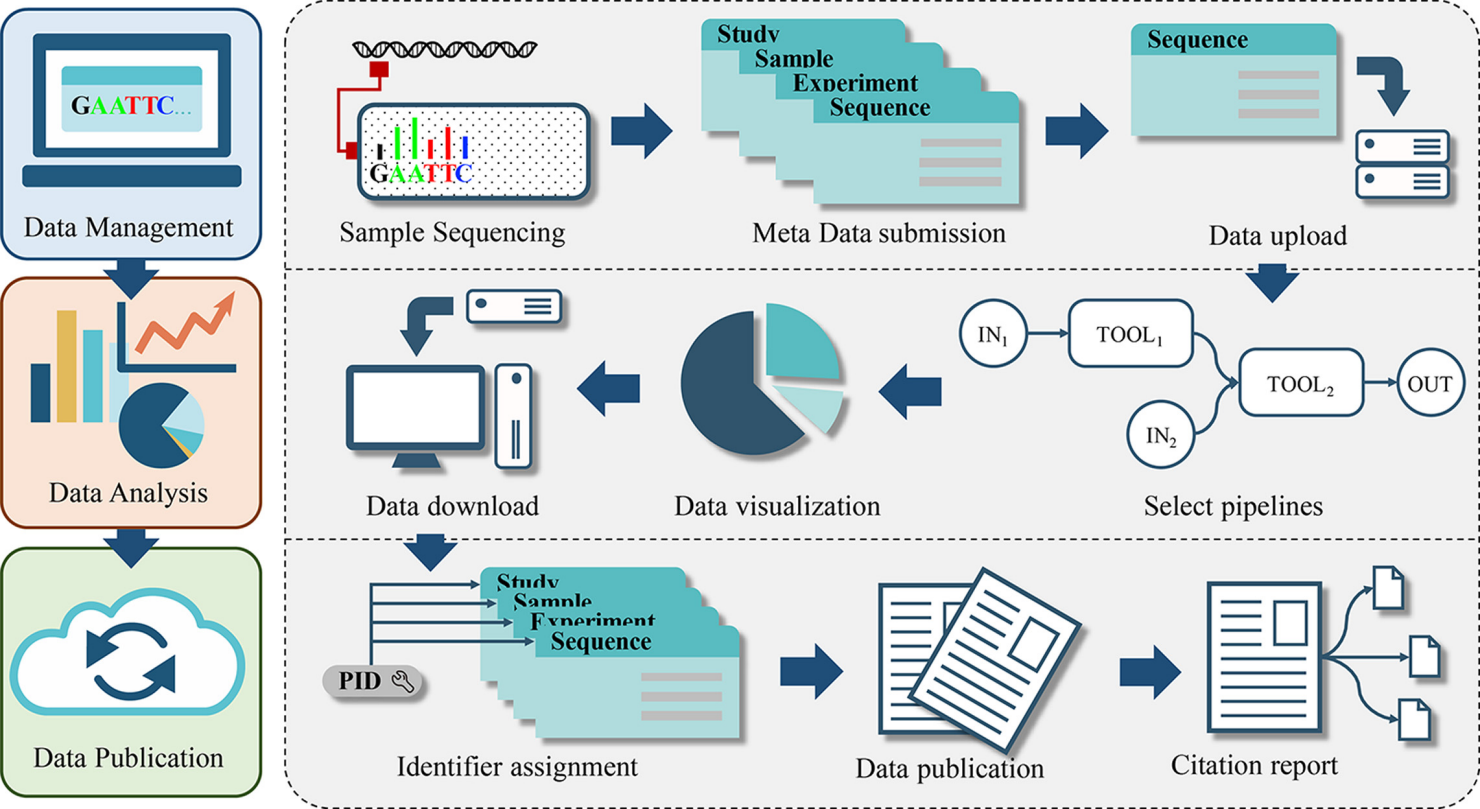
General pipeline of the gcMeta platform. The functional services of gcMeta can be described in three parts: data management, data analysis and data publication. Users submit the meta-data and primary raw data into the system under their own accounts. Users are allowed to analyze the data by preinstalled tools and workflows. Data and results could be downloaded for further analysis. A unique identifier PID would be assigned to each record before the data is public. If the data is further cited in other resources with the PID, the citation could be traced automatically.

The platform supplies management, analysis and publication services for microbiome related data, including genomes, marker genes, metagenomes, metatranscriptomes and their associated metadata (Figure 1). The users can upload the raw data and their metadata into the system via a web submission interface or a data upload web application. After data quality check, the data can be browsed in the system under the user's account. Currently, we provide web-based analysis workflows for marker genes and whole-genome shotgun sequencing (WGS) data. The users can use these workflows or individual tools for data analysis and visualization. The Global Unique Persistent Identifier (GUID) system is used for the open data. To publish the data, users should change the status of their data from ‘private’ to ‘public’, then, the system assigns a persistent identifier (PID) (http://www.pidconsortium.eu/) to the each of the records. The PID is used to cite the data elsewhere and provide a report to the users.

For data protection, login is required before data submission and exploring the full functions. We provide a temporary guest account effective for 24 h along with any submissions, uploaded files and analysis results. All the public available primary raw data or metadata could be downloaded. Access to gcMeta is free at https://gcmeta.wdcm.org/.

### Database design

**Figure 2.**
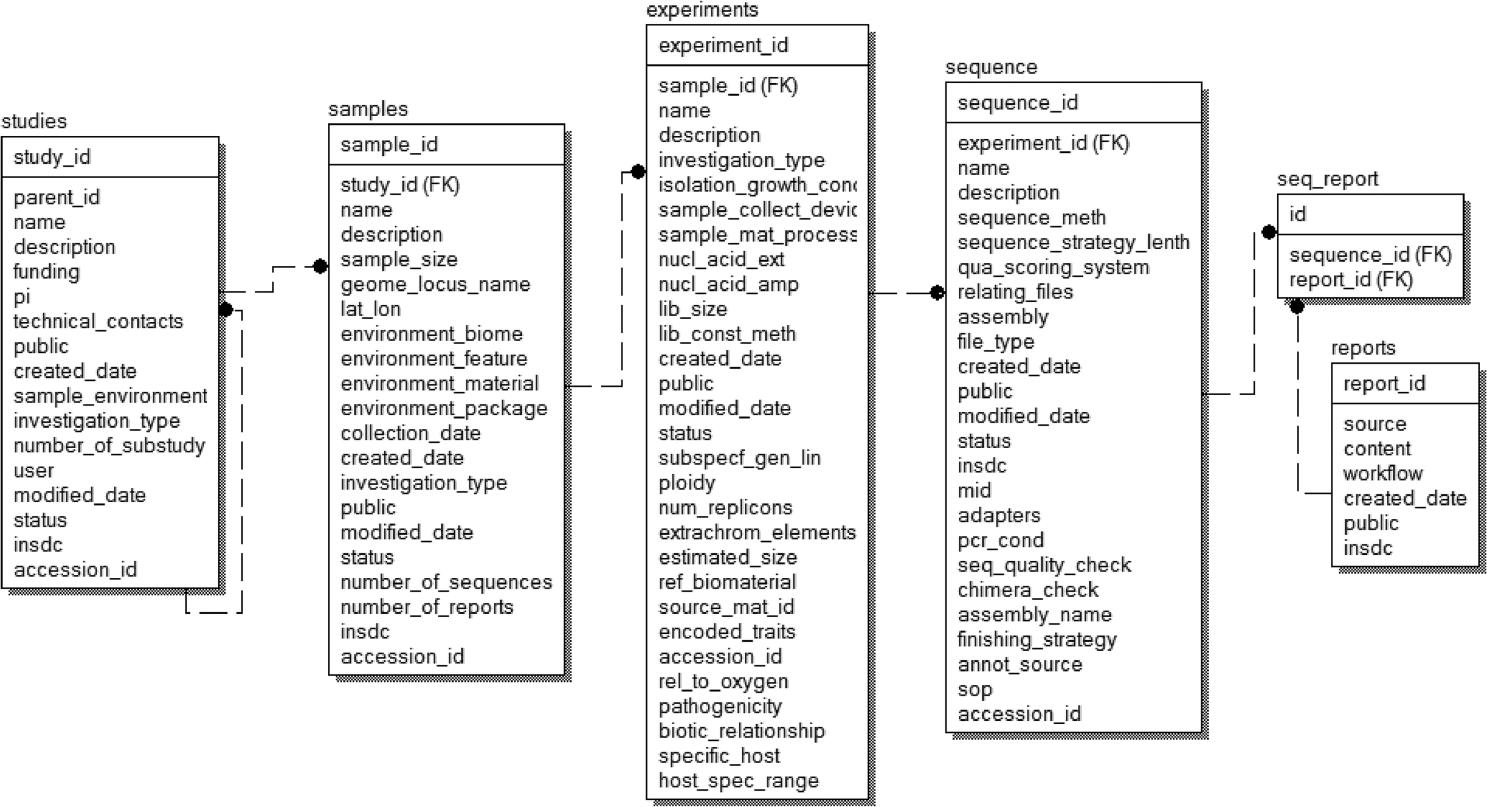
Database schema of gcMeta. Main data structure and relationships between the different tables are illustrated.

The database hosts information on samples and their associate metadata, and primary ‘raw’ data. A relational database is used to host all relevant data. Schema of the database is shown in Figure 2. The major data record types are ‘Study’, ‘Sample’, ‘Experiment’ and ‘Sequence’. ‘Study’ could include several ‘sub-studies’ and is related to ‘Sample’ by the ‘Study ID’. The samples and their associated metadata are recorded. ‘Sample’ is referenced to ‘Experiment’. ‘Experiment’ is further referenced to sequence information. The sequence information contains the sequencing methods and strategies, as well as the processing of the sequencing results, including data quality control and assembly. The gcMeta platform is implemented by an open source database system PostgreSQL.

Ontologies and data standards are crucial to ensure reusability and interoperability of data. To ensure data comparability and consistency between CAS-CMI and public data resources, gcMeta adopts the Minimum Information about Metagenomic Sequence (MIMS) (14) and Minimum Information about a MARKer gene Sequence (MIMARKS) (15). It also uses the Minimum Information of any(x) Sequence (MIxS), which describes 15 different environmental packages to specify the environmental context of a microbial sample. The Environment Ontology (ENVO) (16) for the three environmental metadata fields including environmental biome, feature and material is used to describe the sampling in the system, using a total of 95 controlled terms.

### Data sources

As of August 2018, gcMeta has archived a total of 3053 studies and 124 052 samples, hosting more than 120 TB of sequencing data. We have two major data sources. The first is publicly available data (e.g. MG-RAST, EBI metagenomics and HMP). Publicly available data are integrated based on isolation sources, environmental features and experiment types, and thus allowing data comparison across different data resources. Efforts were made to overcome varying data quality level, format and (lack of) metadata description. Expression was unified according to environmental ontologies. Secondly, gcMeta serves as a data catalogue for the CAS-CMI project and some other ongoing projects in China. This platform has been rapidly expanding, and now hosts CAS-CMI project sample data from waste water, human gut, characteristic Chinese fermented food and so on. The number of samples from these projects is more than 2000 up to now.

### Data management services

**Figure 3.**
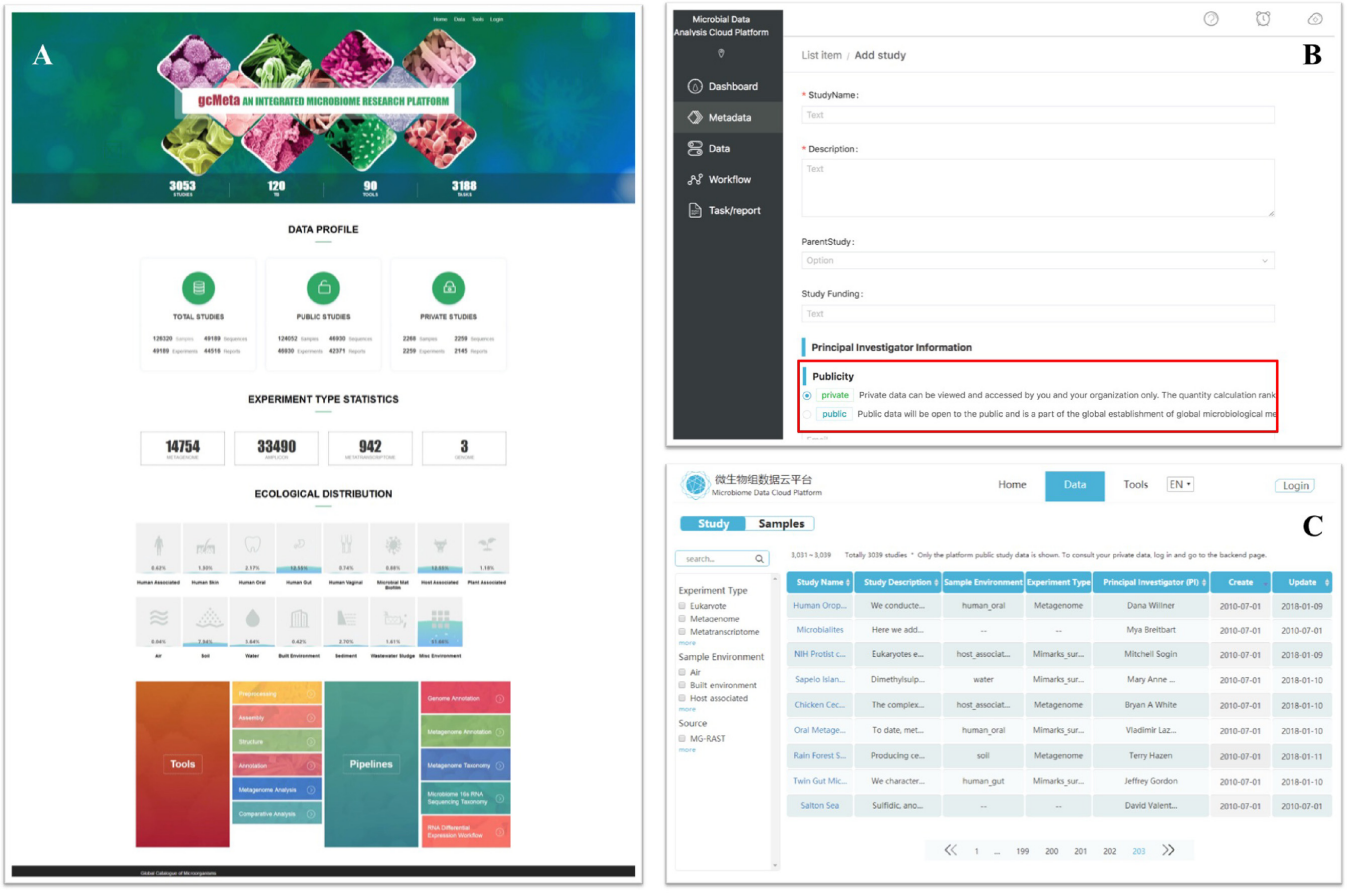
Screenshots and examples of user cases in gcMeta. (**A**) Homepage of the gcMeta. Statistic number of public and private studies, samples, experiments and runs are showed in the homepage. (**B**) A screenshot of data submission by web table. Each entry could be set ‘private’ or ‘public’ as highlighted in the red box. (**C**) A screenshot of database browser. In the search interface, search results could be filtered by ‘experiment type’, ‘sample environment’ and ‘data sources’.

The system allows two routes of data submission. Users can establish a record of their studies and associated samples and experiments online one by one through a web form as indicated in Figure 3. For raw sequence data, users can upload data to the system using a web application. To submit high volume of data in a single session, users can choose a simple tab-delimited file format such as Microsoft Excel. Implementation of data standards occurs during database design as well as prior to the generation of the data in the sampling stage. We also use data validation tools during the on-batch data submission. The system is able to accept data submission from all over the world and offers standardized quality control for the submitted data.

The platform under the CAS-CMI project coordinates with other research institutes, universities, and hospital across China for data repository. Since the ongoing projects are one of the forms of data sources, some data are currently not available to the general public at this stage; data can be accessed via project members only, but it will eventually be publicly available. Data submitters can upload and share their pre-publication data with their research collaborators. Only controlled-access is available for pre-publication data. When required by journals, the data status could be seamlessly switched from ‘private’ to ‘public’ in the system. In this way, we encourage data sharing while protecting data privacy and security. We limit the number of mandatory fields to keep a balance between the burden on contributing scientists and reusability of the data for future analysis. The data could be searched and browsed online after it is submitted.

After the data is set public, PID will be assigned to each ‘Study’ ‘Sample’ ‘Experiment’ and ‘Sequence’ record. The PID is a Global Unique Persistent Identifier system that provides long-term identifying service similar to Digital Object Identifier (DOI) (http://www.doi.org/). An example of PID in gcMeta for ‘Study’ is 21.86101/gcm.study.88c292e8f67c11e7b172b49691092464, where ‘21’ is the handle prefix for PID. ‘gcm.study’ is identifier for ‘Study’ records in the gcMeta database and ‘88c292e8f67c11e7b172b49691092464’ is a randomly assigned series code for the record. The PID can be used to search the Handle (http://hdl.handle.net) site.

### Data analysis and visualization workflows

Metagenomics data are often referred as ‘marker gene amplification metagenomics’ and ‘full shotgun metagenomics’. Depending on the data types, general workflows for data analysis include two different categories. A wide array of tools are currently available to carry out each step of the workflows (17). In gcMeta, we supply analysis tools and workflows which are installed based on Docker technologies, and thus allow users with limited computational resources to perform analysis of metagenomic samples.

### Dockerized analysis tools

More than 90 tools could be used for microbial genomic and metagenomic analysis in web-based interactive mode. These tools are grouped into 6 different categories according to their functions: raw reads preprocessing, sequence assembly, genome structural analysis, database annotation, community profiling and sequence alignment (shown in Table 1). (1) Raw reads preprocessing contains formatting, trimming, filtering, error-correcting and other tools, which are used to reformat or filter the raw data for downstream analysis. (2) Sequence assembly includes assembly, extension and validation tools for both DNA and RNA sequences. Short or long reads become contigs, scaffolds, draft genomes or even complete genomes after this process. (3) Genome structural analysis contains tools for gene prediction, tandem or interspersed repeat detection, CRISPR array detection, etc. The outputs of some tools can be used for further annotation with various databases. (4) Database annotation groups some of the automatic gene annotation tools such as Prokka, DFAST and InterProScan. Formatted databases for annotation are stored in gcMeta for sequence alignment. (5) Community profiling includes tools for classification and quantification, *de novo* binning, community function prediction and downstream analysis both from short reads and contigs of metagenomic data. (6) Sequence alignment contains mapping and alignment, phylogenetic and evolution analysis tools.

**Table 1.** Tools embedded in the gcMeta platform. The tools belong to the group raw reads preprocessing, sequence assembly, genome structural analysis, database annotation, community profiling and sequence alignment are set as red, blue, purple, orange green and yellow respectively. BBtools software suite (http://jgi.doe.gov/data-and-tools/bbtools/), FastQC (http://www.bioinformatics.bbsrc.ac.uk/projects/fastqc/), fastp (https://github.com/OpenGene/fastp/), Trim Galore (http://www.bioinformatics.babraham.ac.uk/projects/trim_galore/), minced (https://github.com/ctSkennerton/minced/tree/master) and RepeatMasker (http://ftp.genome.washington.edu/cgi-bin/RepeatMasker) are all referenced to their websites

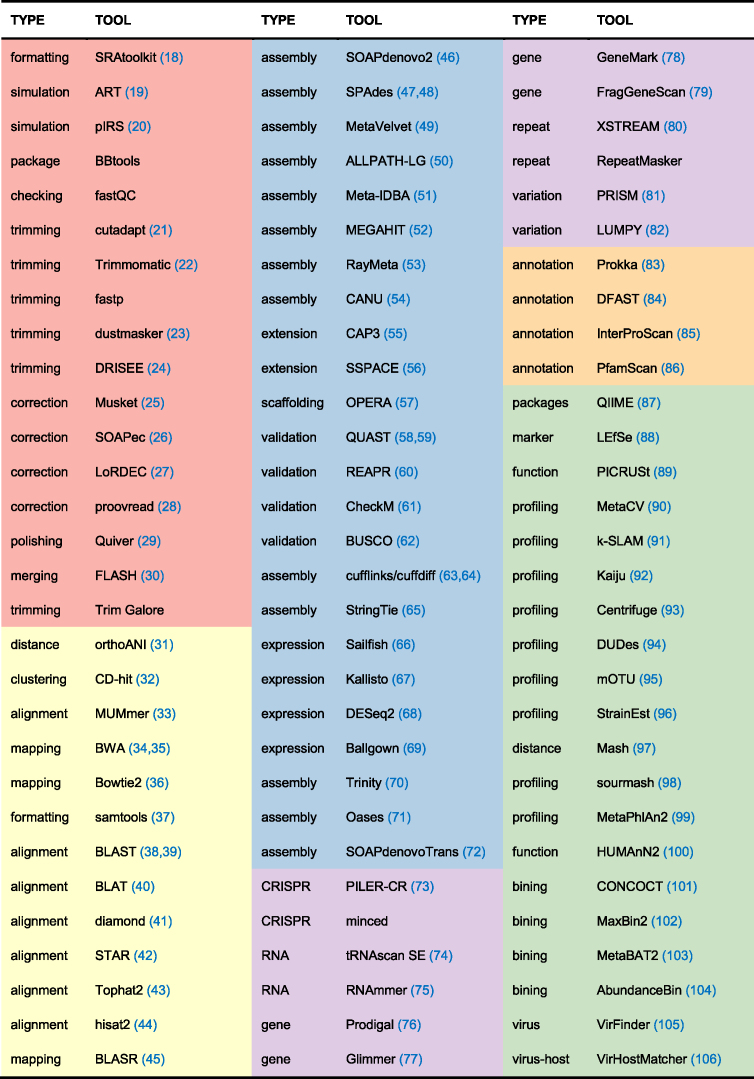

### Integrated analysis workflows

**Figure 4.**
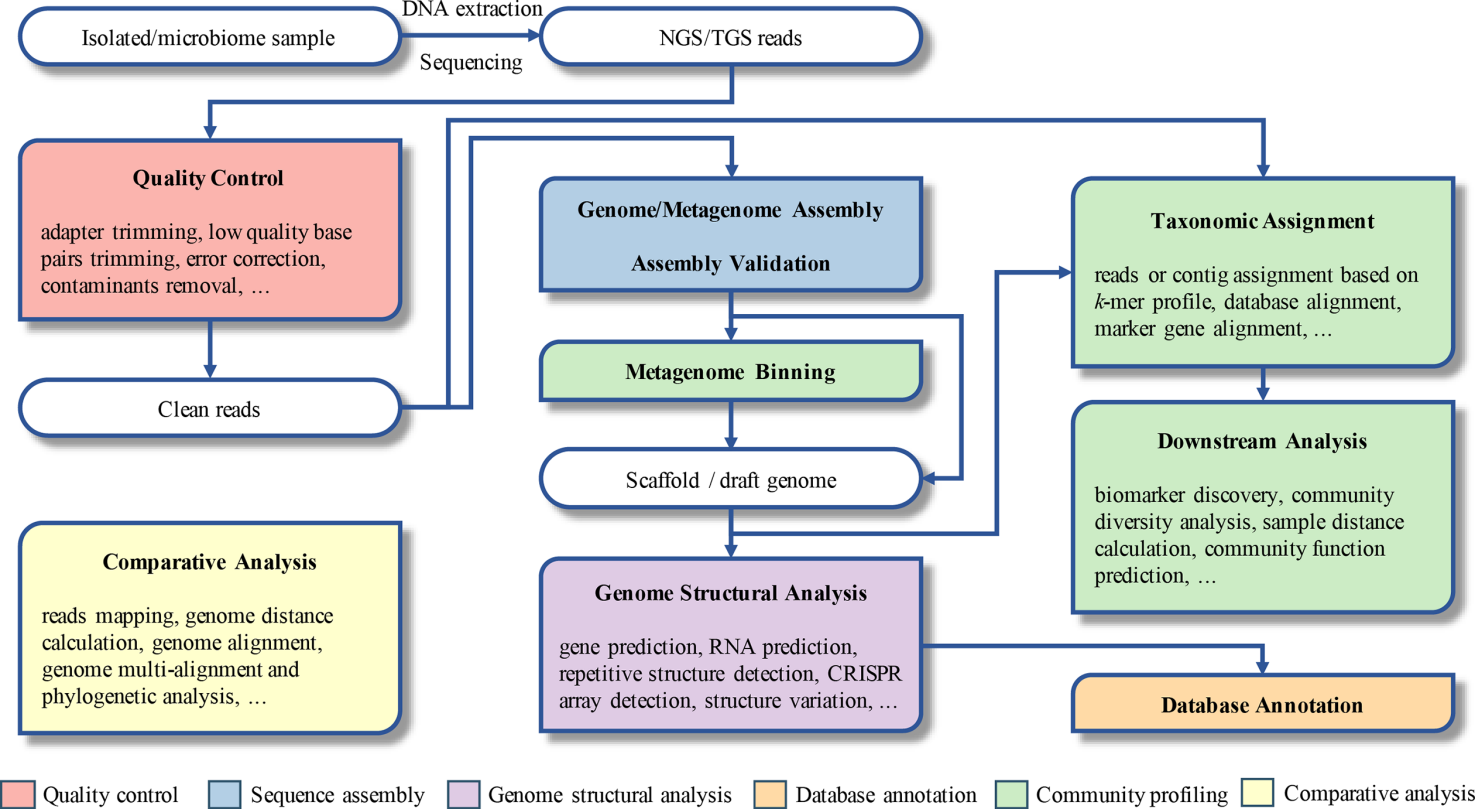
Integrated workflows on gcMeta. The tools can be grouped into 6 clusters shown in different colors (metagenome binning, taxonomic assignment and downstream analysis are all belong to the group community profiling shown in green color). Tools from different functional groups are connected in proper sequence to create workflows. Five main workflows covering different tools according to analysis aims are accessible from a unified user interface exemplified. Comparative analysis tools (shown in yellow) are widely involved in all the workflows. NGS and TGS stands for next-generation sequencing and third-generation sequencing, respectively.

Since outputs of upstream tools can be severed as the inputs of downstream tools, tools can be easily concatenated to achieve a predefined workflow in this platform. Till date, there has been no generally accepted ‘analysis standard’ for a metagenomic analysis workflow. Most workflows involve aspects such as quality control, assembly, binning, taxonomic assignment and functional annotation. However, each workflow is tailored for specific computing resources, aims of analysis and characteristics of the data. In gcMeta, we provide five main workflows for genomes, marker genes, metagenomes analysis. All merged in the workflow overview in Figure 4, and they are:

1. Metagenome assembly and annotation (microbiome sample - NGS reads—quality control—assembly and validation—binning—genome structural analysis—database annotation): In this workflow, we assemble the short reads into contigs. These contigs can be further sorted or binned by similarity to assemble partial to full genomes of microorganisms. The assembled sequences are used for subsequent structural and functional analysis. Firstly, NGS reads, as input, are trimmed into clean reads after performing quality control (host genome contamination removal with Bowtie using parameter ‘very-sensitive’, quality viewing with FastQC and trimming with cutadapt and Trimmomatic). During the host contamination removal process, users can upload the host reference genome or use the index file we provide. Next, clean reads are assembled into contig and scaffold (MEGAHIT). After assembly, contigs and scaffolds are clustered into different bins (MaxBin) and used to perform genome structural analysis (CRISPR detection with PILER-CR, gene prediction with Prodigal, RNA identification with tRNAscan). Then, genes are used to perform annotation (annotation with all referred annotation and alignment tools).
2. Metagenomic 16S rRNA sequencing amplicon taxonomic assignment (microbiome sample—NGS reads—quality control—taxonomic assignment—downstream analysis): As shown in Figure 4, NGS reads, as input, are trimmed and demultiplexed (cutadapt, dada2, demux plugins) with QIIME2 (https://qiime2.org/), and used to perform taxonomic assignment, diversity analysis (diversity analysis, feature-classifier, feature-table, taxa, composition plugins) and phylogenic analysis (phylogeny plugins) with QIIME2 and other downstream analysis (function prediction with PICRUSt and biomarker discovery with LEfSe).
3. Reference based metagenome taxonomic assignment (microbiome sample—NGS reads—quality control—taxonomic assignment—downstream analysis): Read-based taxonomic assignment uses the unassembled DNA or mRNA sequence reads directly and compares them against reference databases to assign taxonomy name to the sequence. NGS reads, as input, are trimmed into clean reads as depicted in workflow 1. Clean reads are then used to perform taxonomic (MetaPhlAn2) and functional assignment (HUMAnN2).
4. Genome assembly and annotation (isolated sample—NGS/TGS reads—quality control—assembly and validation—Genome structural analysis - database annotation): For NGS reads, the workflow is the same as workflow 1, except that there is no contamination removal step in quality control process and contig binning step in assembly process in workflow 4. For TGS reads, as input, are trimmed into clean reads and assembled into contigs and scaffolds with CANU. The draft genomes are then polished with Quiver. Structure analysis and annotation process are the same as in workflow 1.
5. RNA-seq analysis (isolated sample - NGS reads—quality control—alignment—assembly and differential expression analysis): This workflow allows users to identify differentially expressed genes and transcripts by comparing each sample with RNA-seq data. Firstly, NGS reads, as input, are trimmed into clean reads after quality control (quality viewing with FastQC and trimming with TrimGalore). Next, cleaned reads are aligned to the reference genomes with Hisat2. Then, the alignment result is used as an input to assemble transcripts. After assembly, differential expression analysis based on the assembled transcripts will be executed with DESeq2.

**Figure 5.**
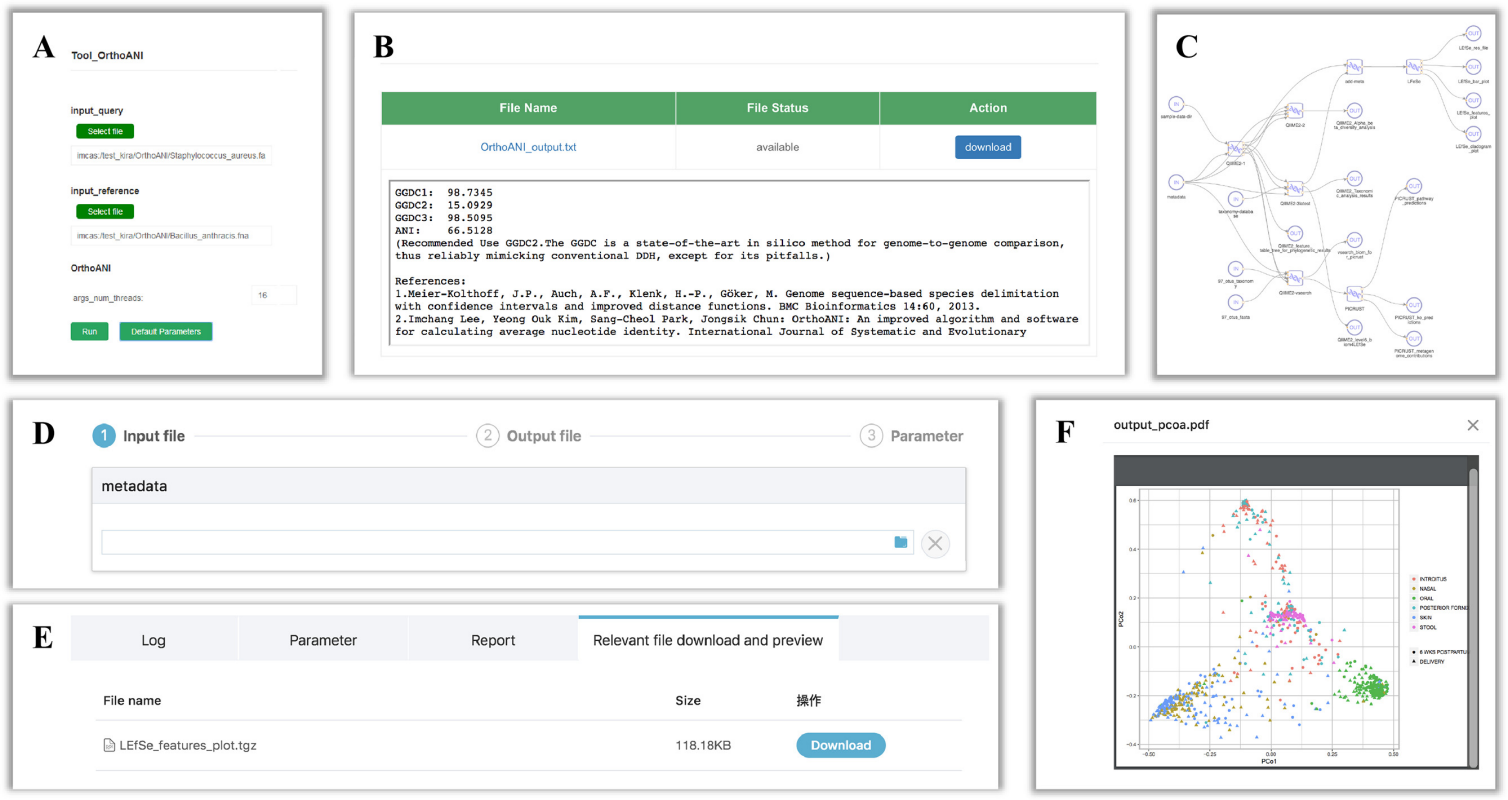
Screenshots of the utility of the tool and workflow. (A, B) The ANI (average nucleotide identity) and dDDH (digital DNA-DNA hybridization) calculation tool which can be used by guest users. (**A**) Screenshots of the job submission including file upload module and necessary arguments setting. (**B**) The results of the job. (C–F) Metagenomic 16S rRNA sequencing taxonomic assignment workflow. (**C**) A screenshot of the sketch of the workflow. (**D**) The screenshots of the inputs, ouputs and arguments settings. (**E**) The result of the workflow. (**F**) The screenshots of the visualization of the analysis result. The example shows PCoA plot generated by ggplot package.

### Implementation and utility of the tools and workflows

The currently available tools and workflows are developed for different server systems, and often difficult to install, configure and deploy by the microbiologists. Since software typically depends on libraries and other components of the installed environment, a workflow implemented in one environment may not work in another environment without extra configuration. To solve this problem, we use the Docker-based technologies. Docker is a Linux-based container technology that allows tools to run across a wide range of operating systems, and helps users to deploy and reproduce tools and workflows without undue efforts (107).

The tools and workflows provide a web-based interface for the users as indicated in Figure 5. The job can be submitted into the tasks queue after the users submit their files into the system and select a specific tool or workflow and set the essential parameters. If sufficient computing resources are not available, the job is put on waiting schedule. Job status is refreshed in the task page for user view. When the job is completed, results could be downloaded and visualized online.

Details of all the tools and workflows, including input format requirements, arguments setting and examples, are described in the Supplementary file and online manual https://gcmeta.wdcm.org/im/manual/index.jsp.

### System design and implementation

**Figure 6.**
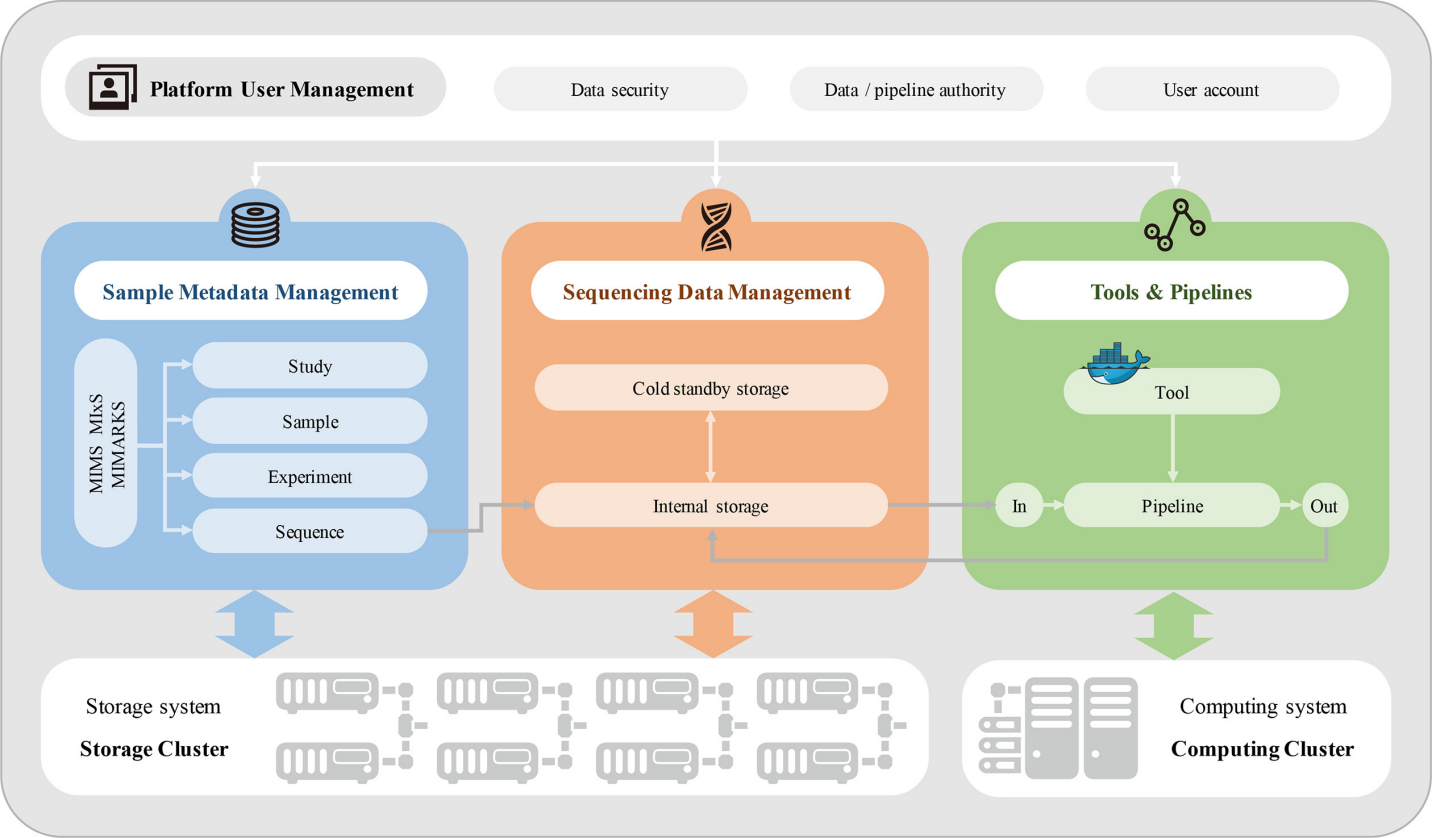
System structure of gcMeta. The platform integrates storage cluster and computing cluster resources with database management system and Docker based tools and workflows to supply comprehensive data archive, publication and analysis service to users.

The system as indicated in Figure 6 is based on centralized computing and storage resources. The database management system is divided into metadata management, sequencing raw data management and user information management. The current version of the gcMeta database is constructed on the basis of PostgreSQL for metadata and user information, and MongoDB for sequencing raw data index information. The system is operated on Linux servers. The web interface was developed using Python. Tools and workflows were installed with Docker technologies.

## DISCUSSION

With the vast diversity of microbial communities and exponentially increasing amount of meta-omics data, we are facing great challenges in organized data management and deep data mining. As a part of the efforts of CAS-CMI, gcMeta provides long-term data preservation and management following the internationally used standards and hence serves as a public data repository. We provide data protection for pre-publication data and GUID for public data, which ensures the reuse of data as required by the scientific community. The platform houses and incorporates data from public and ongoing microbiome projects, and supports comparative analysis of the data collected from distinct projects.

Another key feature of the platform is we offer a set of data analysis and visualization tools and predefined workflows by web interface which facilitates data analysis by microbiologists in an easy way. As the system is based on the Docker technology, it can be run by a variety of operational systems. The analysis application is integrated with the in-house computing resources which provide scientists a robust site for powerful data analysis.

We will keep updating the database and workflows to support the rapidly increasing datasets and complicated studies. gcMeta accepts data submission for single genome, microbiome and transcriptome data. Meanwhile, we will establish connection with other data portals to ensure data properly deposited and preserved within the International Nucleotide Sequence Database Consortium (INSDC). On the other hand, the meta-omics data analysis is becoming more and more diverse. Predefined workflows could not fit for all the analysis aims. Therefore, the future version of gcMeta will provide customized workflows for professional bioinformatics who are interested in the data and computing resources while hope to develop their own analysis workflows.

Additionally, current meta-omics data and associated analysis results are widely dispersed in different kinds of resources from public data archives to specialized databases. Integration of various types of meta-omics data is essential for a comprehensive understanding of the structure, functions and expressions of a specific community, species, strain or gene. Updating our database schema to accommodate diverse data and providing rational links among those data through semantic web technologies are future planned directions as well.

Moreover, with increasing data from microbiome projects in China and worldwide, high quality reference data are needed for accurate data annotation. However, the current type strain genomic data resources in the public database are still unable to fullfill the requirements. The Global Catalogue of Microorganisms (GCM) 2.0 type strains (108) sequencing project and The Genomic Encyclopedia of Bacteria and Archaea (GEBA) project (109) are the ongoing efforts to fill in this gap. We plan to integrate the GCM 2.0 sequencing outputs into our system to provide more accurate annotation of metagenomics data. In conclusion, gcMeta will continuously improve to accommodate the evolving meta-omics researches.

## Supplementary Material

Supplementary Data
