# Supplementary material for: gcMeta: a Global Catalogue of Metagenomics platform to support the archiving, standardization and analysis of microbiome data
Source: Nucleic Acids Res. 2018 Oct 26;47(Database issue):D637–48. doi: 10.1093/nar/gky1008 (PMC6324004; doi:10.1093/nar/gky1008)
Supplement: Supplementary Data [file gky1008_supplemental_files.docx]

**gcMeta Tools and Workflows Setting**

**MANUAL v1.0**

**Web site: https://gcmeta.wdcm.org/**

**Bowtie2 (reads alignment)**

Introduction: Bowtie 2 is an ultrafast and memory-efficient tool for aligning sequencing reads to long reference sequences. It is particularly good at aligning reads of about 50 up to 100s or 1,000s of characters, and particularly good at aligning to relatively long (e.g. mammalian) genomes.

Input: Paired-end reads and reference genome (in fasta format).

Output: Alignment results (in SAM format).

Test inputs:

input_reference (fasta/fa/fna)

imcas:/test_kira/Lactobacillus.fasta

input_seq1 (fastq/fq)

imcas:/test_kira/simulate/pirs_100_600_1.fq

input_seq2 (fastq/fq)

imcas:/test_kira/simulate/pirs_100_600_2.fq

Test arguments:

args_threads (integer, default=1~16, [NULL])

Test example:

?.sam 313.52MB

**BWA (reads alignment)**

Introduction: BWA is a software package for mapping low-divergent sequences against a large reference genome. It consists of three algorithms: BWA-backtrack, BWA-SW and BWA-MEM. BWA-MEM and BWA-SW share similar features such as long-read support and split alignment, but BWA-MEM, which is the latest, is generally recommended for high-quality queries as it is faster and more accurate. BWA-MEM also has better performance than BWA-backtrack for 70-100bp Illumina reads.

Input: Paired-end reads (in fastq format) and reference genome (in fasta format).

Output: Alignment results (in SAM format).

Test inputs:

input_seq1 (fastq/fq)

imcas:/test_kira/simulate/pirs_100_600_1.fq

input_seq2 (fastq/fq)

imcas:/test_kira/simulate/pirs_100_600_2.fq

input_reference (fasta/fa/fna)

imcas:/test_kira/simulate/Lactobacillus.fasta

Test arguments:

args_threads (integer=1~16, [NULL])

args_mark_shorter_split (optional, [TRUE]) # **for BWA-MEM**

Test example:

*.sam 291.63MB (BWA-MEM)

*.sam 321.35MB (BWA-ALN)

*.sam 138.58MB (BWA-SW)

Supplementary:

MEM is used for performing split mapping. SW is used for performing long reads mapping (700bp~1000bp). ALN is used for performing short reads mapping (<100bp).

**fastQC (quality control)**

Introduction: A quality control tool for high throughput sequence data.

Input: Raw sequencing data (in fastq format).

Output: Quality control report (zip file including html&zip).

Test inputs:

input_seq (fastq/fq/gz)

imcas:/test_kira/soapec/yeast_test_1.fq

imcas:/test_kira/soapec/yeast_test_2.fq

Test arguments:

args_threads (integer=1~16, [NULL])

Test example:

?.tgz 1002.70KB

Unzip it, tempoutput includes:

?_fastqc.html 627K, 622K

?_fastqc.zip 336K, 337K


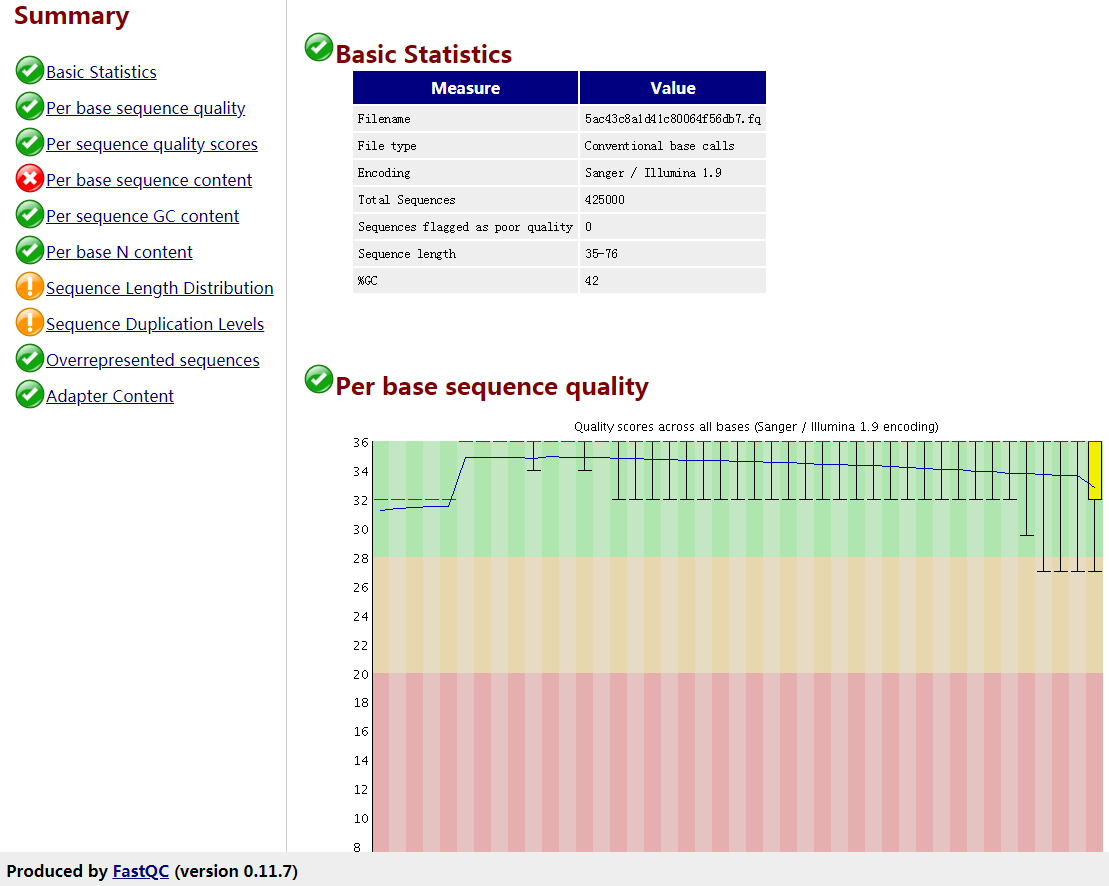


Figure: fastQC html report

**fastp (quality control)**

Introduction: A tool designed to provide fast all-in-one preprocessing for FastQ files.

Input: Raw sequencing data (in fastq format).

Output: Quality control report (in html&json format) and cleaned paired-end reads (in fastq format).

Test inputs:

input_seq (fastq/fq/gz)

imcas:/test_kira/soapec/yeast_test_1.fq

imcas:/test_kira/soapec/yeast_test_2.fq

Test arguments:

args_cut_mean_quality (the bases in the sliding window with the lowest mean quality, integer=10~30, [NULL=20])

args_threads (integer=1~16, [NULL])

Test example:

?.fastq 86.51MB

?.fastq 86.46MB

?.html 426.20KB

?.json 97.53KB


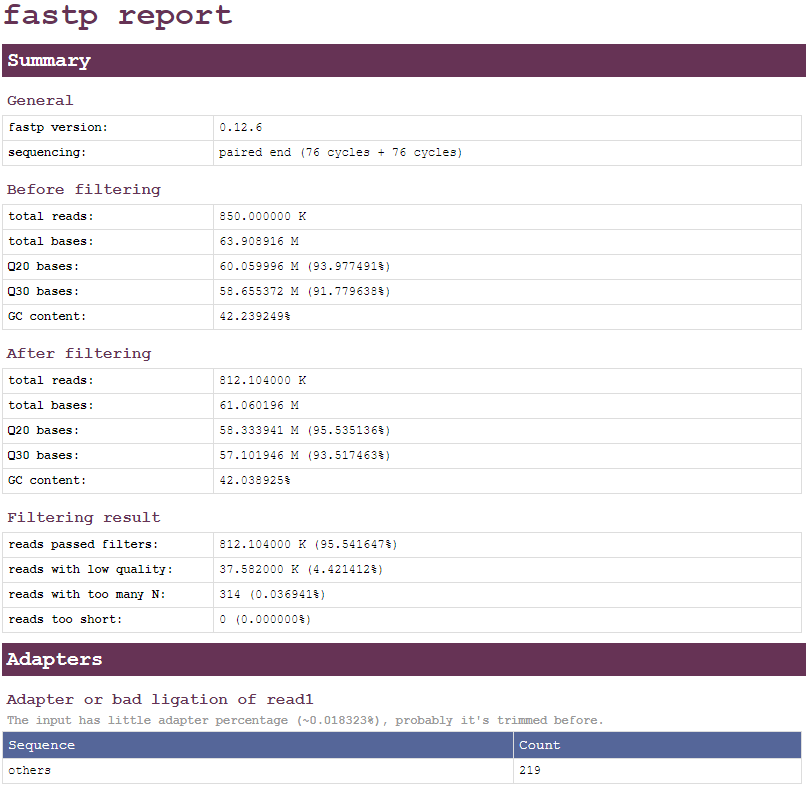


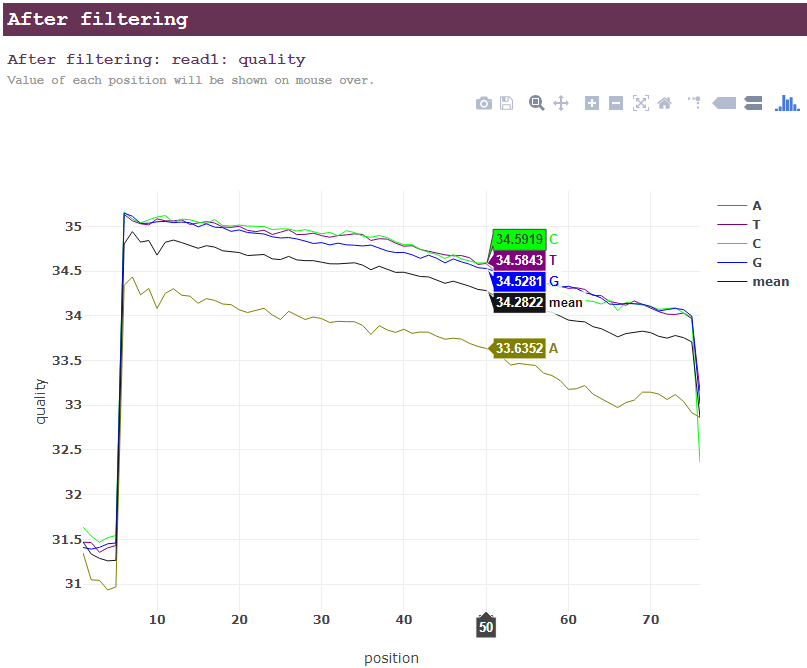


Figure: fastp html report

**MUSCLE (multiple alignment)**

Introduction: MUSCLE is one of the best-performing multiple alignment programs according to published benchmark tests, with accuracy and speed that are consistently better than CLUSTALW.

Input: Multiple sequences (in fasta format).

Output: Alignment results (in fasta/CLUSTALW/HTML/MSF format).

Test inputs:

input_fasta (fasta/fa/fna)

imcas:/test_kira/muscle/multi-genes.fa (5 homologous genes)

Test arguments:

args_fmt (output format, enum=-fasta|-msf|-html|[-clw]) -clw

Test example:

?.txt 11.61KB


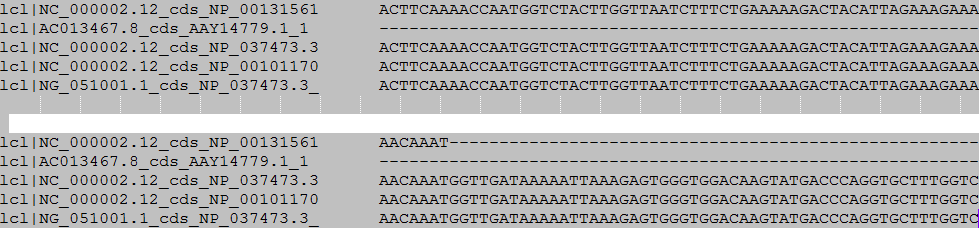


Figure: clustal format alignment result

**fastq-dump (format conversion)**

Introduction: fastq-dump is a subfunction of SRA (Sequence Read Archive) Toolkit which is used to convert sra format into fastq format.

Input: SRA format file

Output: fastq format file

Test inputs:

input_sra (sra)

imcas:/test_kira/sra/SRR5511068.sra

Test example:

?.fastq 495.96MB, 496.18MB

**SAMtools-view (format conversion)**

Introduction: SAM file <=> BAM file.

Input&Output: SAM file & BAM file

Test inputs:

input_sam & inpput_bam (SAM&BAM)

imcas:/[test_kira](javascript:;)/SAMtools/yeast.sam

imcas:/[test_kira](javascript:;)/SAMtools/yeast.bam

**SAMtools-sort** **(format conversion)**

Introduction: The sort command sorts a BAM file based on its position in the reference, as determined by its alignment.

Input: BAM file

Output: sorted BAM file

Test inputs:

input_bam (BAM)

imcas:/test_kira/SAMtools/yeast.bam

**SAMtools-mpileup (variant calling)**

Introduction: The mpileup command produces a pileup format (or BCF) file giving, for each genomic coordinate, the overlapping read bases and indels at that position in the input BAM files(s). This can be used for SNP calling for example.

Input: reference genome (in fasta format) and sorted BAM file (output file from SAMtools-sort, in bam format).

Output: binary counterpart of variant call format file (in BCF format)

Test inputs:

input_bam (bam)

imcas:/test_kira/SAMtools/yeast.sorted.bam

input_reference (fas/fasta/fna/fa)

imcas:/test_kira/SAMtools/GCF_000146045.2_R64_genomic.fna

Test example:

?.bcf 861.10MB

**SAMtools-index (format conversion)**

Introduction: The index command creates a new index file that allows fast look-up of data in a (sorted) SAM or BAM. (Note: You must sort the bam files before you can index)

Input: sorted BAM file (output file from SAMtools-sort, in bam format)

Output: bai file

Test inputs:

input_bam (bam)

imcas:/test_kira/Samtools/yeast.sorted.bam

**BCFtools-view (format conversion)**

Introduction: Convert between VCF and BCF. Calling variant candidates and estimate allele frequencies.

Input: bcf file (output file from Samtools-mpileup)

Output: vcf file (Variant Call Format file)

Test inputs:

input_bcf (bcf)

/[test_kira](javascript:;)/Samtools/yeast.bcf

**SAMtools-flagstat (statistics)**

Introduction: Give the comparison result of BAM file.

Input&output: BAM file & txt file

Test inputs:

input_bam (bam)

/test_kira/SAMtools/yeast.bam

Test example:

?.txt 388.00B


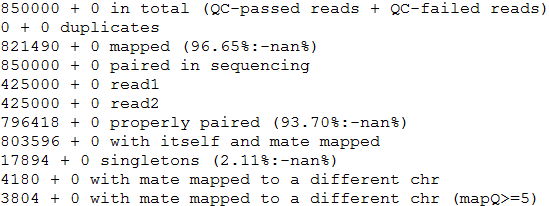


Figure: the report of the mapping result

**QUAST (assembly validation)**

Introduction: QUAST stands for QUality ASsessment Tool. The tool evaluates genome assemblies by computing various metrics.

Input: assembly fragment sequences (in fasta format, multiple file is allowed), reference genome (in fasta format) and its genes and operons annotation (in gff or text format).

Output: assembly validation report (in zip format).

Test inputs:

input_query (fasta/fa)

imcas:/test_kira/QUAST/SOAPdenovo.fa

imcas:/test_kira/QUAST/SPAdes.fasta

input_reference (gz/fasta/fa/fna)

imcas:/test_kira/Lactobacillus.fasta

input_genes (gff/txt)

input_operons (gff/txt)

Test arguments:

args_m (Lower threshold for contig length, integer, [500]) 500

args_s (Assemblies are scaffolds, split them and add contigs to the comparison, flag, [NO]) **True**

args_e (Genome is eukaryotic, flag, [NO]) **True**

Test example:

?.tgz 658.55KB

Unzip it, in quast_results/results_date_time

Please open report.html


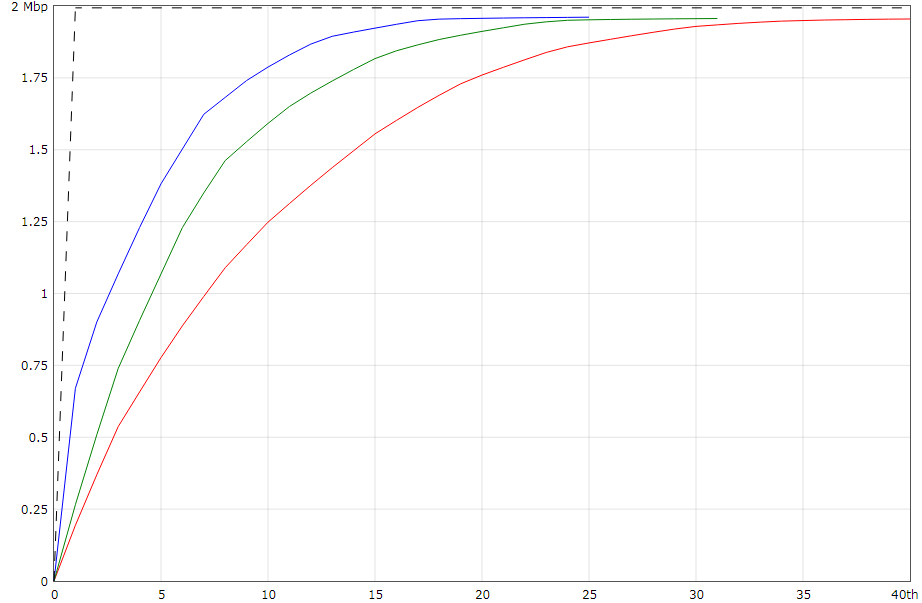


Figure: cumulative length plot describe the result of SPAdes is better.

Click View in Icarus contig browser


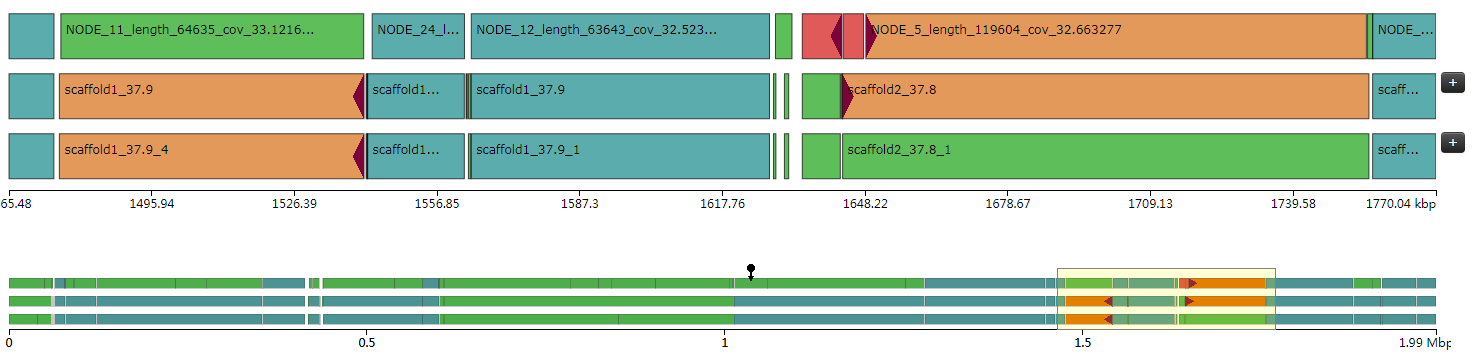


Figure: contig alignment viewer

**SOAPdenovo2 (genome assembly)**

Introduction: SOAPdenovo is a novel short-read assembly method that can build a de novo draft assembly for the human-sized genomes. The program is specially designed to assemble Illumina GA short reads. It creates new opportunities for building reference sequences and carrying out accurate analyses of unexplored genomes in a cost effective way.

Input: Paired-end sequencing reads, two libraries are supported (in fasta or fastq format).

Output: Assembled contig and scaffold (in fasta format) and assembly report.

Test inputs:

input_pe11 (fasta/fa/fastq/fq)

imcas:/test_kira/simulate/pirs_100_600_1.fq

input_pe12 (fasta/fa/fastq/fq)

imcas:/test_kira/simulate/pirs_100_600_2.fq

Test arguments:

args_avg_ins1 (integer) **600**

args_reverse_seq1 (forward-reverse=0, reverse-forward=1, integer, [0]) 0

args_asm_flags1 (contig=1, scaffold=2, contig&scaffold=3, integer, [3]) 3

args_rank1 (order to run the library, integer, [1]) 1

args_kmerFreqCutoff (kmers with frequency no larger than KmerFreqCutoff will be deleted, integer, [0]) 0

args_edgeCovCutoff (edges with coverage no larger than EdgeCovCutoff will be deleted, interger, [1]) 1

args_kmer (integer=13~127, [23]) **17**

args_maxKmer (maximum kmer size used for multi-kmer, integer=kmer~127, [NULL]) **31**

args_mergeLevel (the strength of merging similar sequences during contiging, integer=0~3, [1]) 1

args_resolverepeats (resolve repeats by reads, flag, [NO]) **true**

args_fillgaps (fill gaps in scaffolds, flag, [NO]) **true**

args_cpu (number of cpu for use, integer, [8]) 8

Test example:

?.fa (contig) 1.94MB

?.fa (scaffold) 1.90MB

?.txt 1.66KB

Supplementary:

input_pe21, input_pe22, args_avg_ins2, args_reverse_seq2, args_asm_flags2, args_rank2 means the arguments of the second library.

**SPAdes (genome assembly)**

Introduction: SPAdes – St. Petersburg genome assembler – is an assembly toolkit containing various assembly pipelines.

Input: Paired-end sequencing reads, two libraries are supported (in fasta or fastq format).

Output: Assembled scaffold (in fasta format).

Test inputs:

input_pe11 (fasta/fa/fastq/fq)

imcas:/test_kira/simulate/pirs_100_600_1.fq

input_pe12 (fasta/fa/fastq/fq)

imcas:/test_kira/simulate/pirs_100_600_2.fq

Test arguments:

kmersize (comma-separated list of k-mer sizes and must be odd and less than 128, string) 17,31

type (perform common/single-cell/metagenome/rna/plasmid assembly, enum=[NULL] /sc/meta/rna/plasmid)

careful (tries to reduce number of mismatches and short indels, flag, [False]) **true**

threads (number of threads, integer=, [16]) 16

onlyassembler (runs only assembling without read error correction, flag, [False])

Test example:

?.fasta 1.91MB

**CANU (genome assembly for long reads)**

Introduction: Canu is a fork of the Celera Assembler designed for high-noise single-molecule sequencing (such as the PacBio RSII or Oxford Nanopore MinION).

Input: pacbio/nanopore/tru-seq reads (in fasta, fastq or zip format).

Output: Assembled sequences (in fasta format) and assembly report.

Test inputs:

input_pacbio (fastq/fq/fasta/fa/gz)

imcas:/test_kira/pacbio/pacbio.fastq

Test arguments:

args_genomesize (estimated genome size, eg: ecoli genomesize=4.8M, float) **5**

args_type (sequencing method, enum=-nanopore-raw|[-pacbio-raw])

Test example:

?. fasta 4.47MB

?.txt 60.97KB

?.tgz 162.38MB (All assembly detail files)


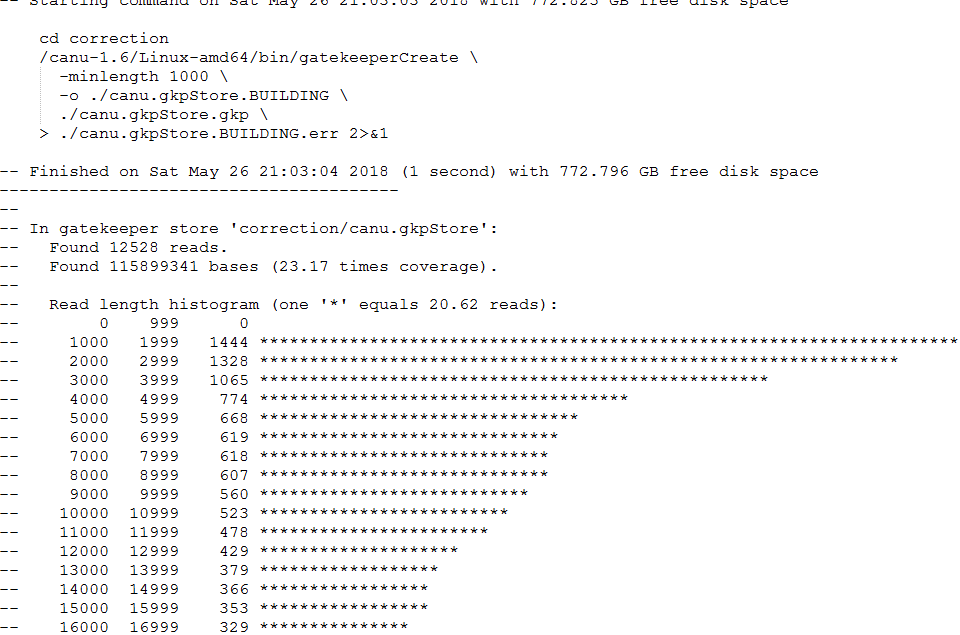


Figure: CANU report (txt output file)

**XSTREAM (tandem repeat detection)**

Introduction: XSTREAM is a tool for rapidly identifying and modeling the architecture of “fundamental” Tandem Repeats (TRs) in protein sequences. Due to the general nature of TRs, however, any sequence including DNA (or even numbers!) can be processed.

Input: Any sequence even numbers (in fasta or text format)

Output: Tandem repeat sequence, position, alignment and other information (in zip format).

Test inputs:

input_seq (fa/fasta/txt/fna)

imcas:/test_kira/repeatProtein.fa

Test arguments:

args_t (use more than one CPU, integer, [1])

args_e (minimum copy number, integer, [2]) **3**

args_m (minimum length of repeat element, integer, [3])

arg_x (maximum length of repeat element, integer, [NULL= unlimited])

args_g (maximum gaps between repeat element, integer, [3])

args_i (minimum word match, float, [0.7])

args_I (minimum consensus match, float, [0.8])

args_D (maximum indel error, float, [0.5])

Test Example:

?.tgz 54.58KB

Unzip it, 3 html files are included:

*_1.html is the summary of all the repetitive sequences.

*_2.html is the structure of all the repetitive sequences.

*_3.html is the sequences.


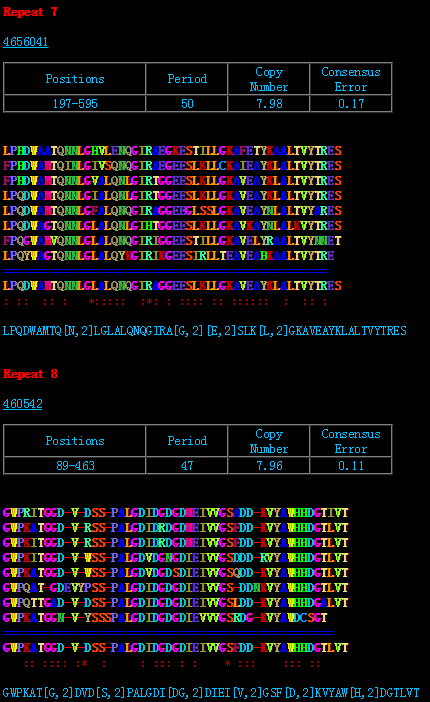


Figure: structure of all the repetitive sequences

**SOAPec (error correction)**

Introduction: SOAP error correction module.

Input: Error-existing reads (in fasta or fastq format)

Output: Error-pruned reads (in fasta or fastq format)

Test inputs:

input_seq1 (fastq/fq/fasta/fa)

imcas:/test_kira/soapec/yeast_test_1.fq

input_seq2 (fastq/fq/fasta/fa)

imcas:/test_kira/soapec/yeast_test_2.fq

Test arguments:

args_threads (set the thread number, integer, [1]) **4**

args_kmer (set the kmer size, integer=11~17, [17]) **15**

args_lowfreqcutoff (the low frequency cutoff of consecutive kmer, integer, [3]) 3

args_ lengthcutoff (the minimum length of trimmed read, integer, [50]) **40**

args_spaceseed (Set the size of space-seed in kmer, k=10, s=5: 'ATTCG-----GTACG', k=11, s=4: 'ATTCG--A--GTACG', integer, [0]) 0

Test Example:

?.fastq 64.59MB

?.fastq 64.54MB

**NUCmer (long sequence alignment)**

Introduction: MUMmer is a system for rapidly aligning entire genomes, whether in complete or draft form.

Input: The alignment query and reference sequence (in fasta format)

Output: The alignment coordinate (in text format)

Test inputs:

input_reference (fasta/fa/fna)

imcas:/test_kira/NC_011740.1.fa

input_query (fasta/fa/fna)

imcas:/test_kira/NC_002695.1.fa

Test Example:

?.coords 94.12KB


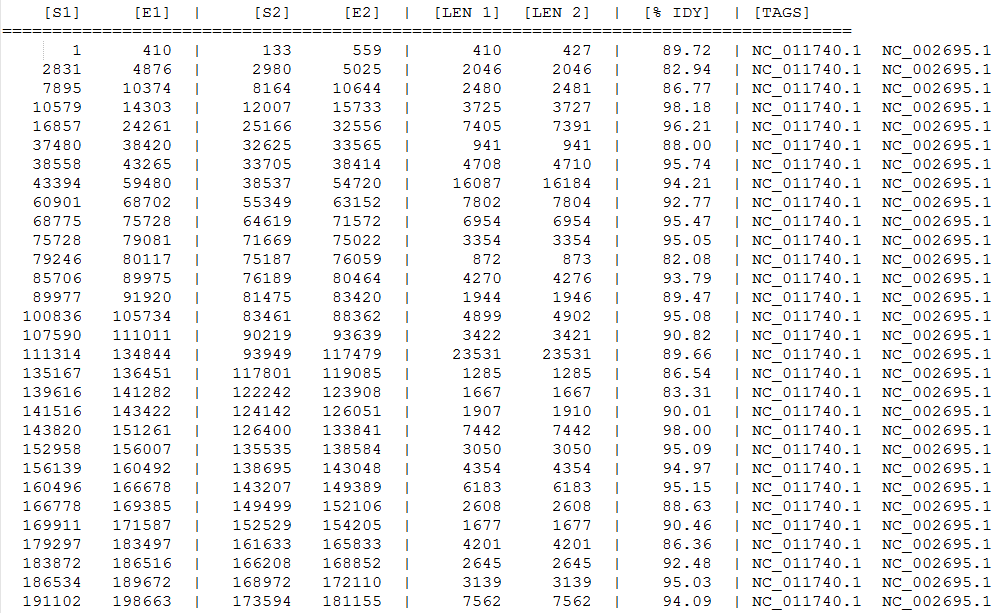


Figure: alignment coordinate output

**Prodigal (gene recognition)**

Introduction: Prodigal is a protein-coding gene prediction software tool for bacterial and archaeal genomes. The acronym stands for PROkaryotic DYnamic Programming Genefinding ALgorithm.

Input: nucleotide sequence (in fasta or zip format).

Output: predicted gene (in cds, gff and pep format)

Test inputs:

input_file (fna/fasta/fa/gz)

imcas:/test_kira/Lactobacillus.fasta

Test arguments:

arg_procedure (enum=meta|[single]) single

Test example:

?.gff 424.51KB

?.pep 822.01KB

?.cds 1.94MB


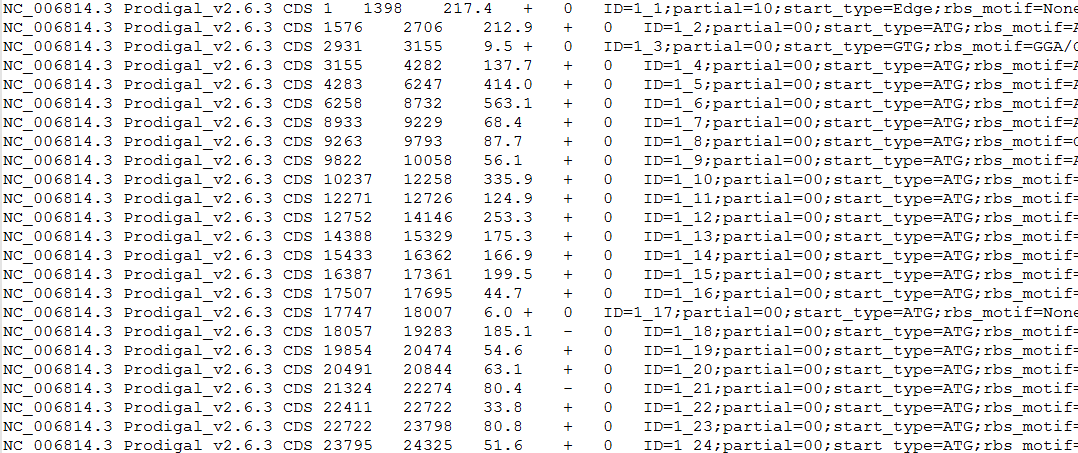


Figure: gff3 output

Supplementary (gff3 format):

Column 1: "seqid", the combination of [a-zA-Z0-9.:^*$@!+_?-|]

Column 2: "source", eg: "Genescan", "Genbank", "." stands for empty

Column 3: "type", eg: "Gene", "cDNA", "mRNA"

Column 4&5: "start"&"end"

Column 6: "score"

Column 7: "strand", eg: "+", "-", "?"

Column 8: "phase", for CDS, start position of coding nt = 0, 1, 2

Column 9: "attributes"

**Trimmomatic (quality control)**

Introduction: A flexible read trimming tool for Illumina NGS data.

Input: raw sequencing reads (in fastq format).

Output: cleaned reads including forward-paired, forward-unpaired, reverse-paired, reverse-unpaired (in fastq format).

Test inputs:

input_forward (fastq/fq/gz)

imcas:/test_kira/soapec/yeast_test_1.fq

input_reverse (fastq/fq/gz)

imcas:/test_kira/soapec/yeast_test_2.fq

Test arguments:

args_threads (integer) 4

args_minlen (set the minimum length of the reads, integer) 40

args_leading (Cut bases off the start of a read, if below a threshold quality, integer) 3

args_trailing (Cut bases off the end of a read, if below a threshold quality, integer) 3

args_windowsize (Perform a sliding window trimming, set the widow size, integer) 4

args_windowqual (Perform a sliding window trimming, set the minimum average quality, integer) 20

args_adapters (Cut adapter and other illumina-specific sequences from the read , enum=TruSeq3-SE.fa|[TruSeq3-PE.fa]|TruSeq3-PE-2.fa|TruSeq2-SE.fa|TruSeq2-PE.fa|NexteraPE-PE.fa)

Test example:

?.fp.fq 85.18MB (forward-paired)

?.rp.fq 85.01MB (reverse-paired)

?.fu.fq 626.47KB (forward-unpaired)

?.ru.fq 4.03MB (reverse-unpaired)

**MetaPhlAn2 (metagenome WGS profiling)**

Introduction: MetaPhlAn is a computational tool for profiling the composition of microbial communities (Bacteria, Archaea, Eukaryotes and Viruses) from metagenomic shotgun sequencing data (i.e. not 16S) with species-level.

Input: metagenome WGS sequencing reads (in fasta, fastq or zip format).

Output: the profile of metagenome sample (in text format).

Test inputs:

input_file (fa/fasta/fq/fastq/gz/bz) **multiple sequences is supported!**

imcas:/test_kira/metaphlan2/*

Test arguments:

multipleproc (multiple thread, integer) 16

inputtype (enum=[fastq]|fasta|multifasta|multifastq) **fasta**

Test example:

?.txt 10.68KB

**PICRUSt (metagenome 16s function )**

Introduction: PICRUSt (pronounced “pie crust”) is a bioinformatics software package designed to predict metagenome functional content from marker gene (e.g., 16S rRNA) surveys and full genomes.

Input: OTU file (in biom format).

Output: KO (KEGG Orthology) predictions, pathway predictions (in biom format) and metagenome contributions (in txt format).

Test inputs:

input_file (biom)

imcas:/[test_kira](javascript:;)/PICRUSt/otus.biom

Test arguments:

args_type_of_prediction (type of functional predictions, enum=[ko]|cog|rfam) ko

args_metadata_category (the metadata category that describes the hierarchy, enum= [KEGG_Pathways]|COG_Category) KEGG_Pathways

args_level (the level in the hierarchy to collapse to, integer = 1~9, [3]) 3

args_limit_functions (If provided, only output predictions for the specified function ids. Multiple function ids can be passed using comma delimiters, string)

**K01727,K01194,K01216,K11049,K00389,K00449**

Test example:

?.biom 2.01MB **KO predictions**

?.biom 153.43KB **pathway predictions**

?.txt 281.35KB


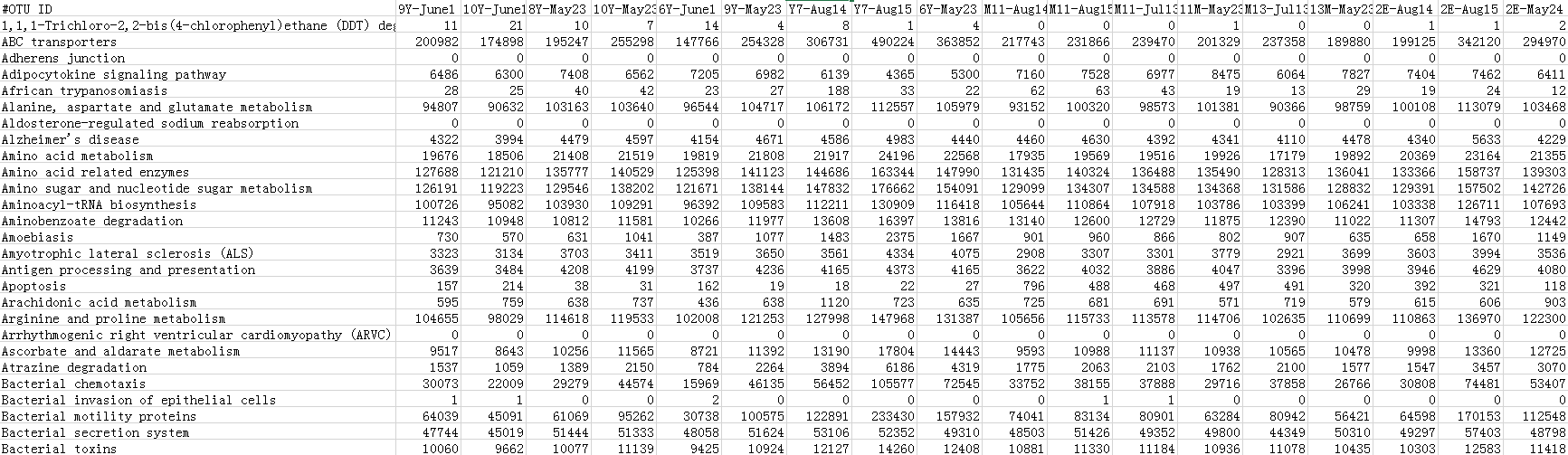


Figure: pathway table


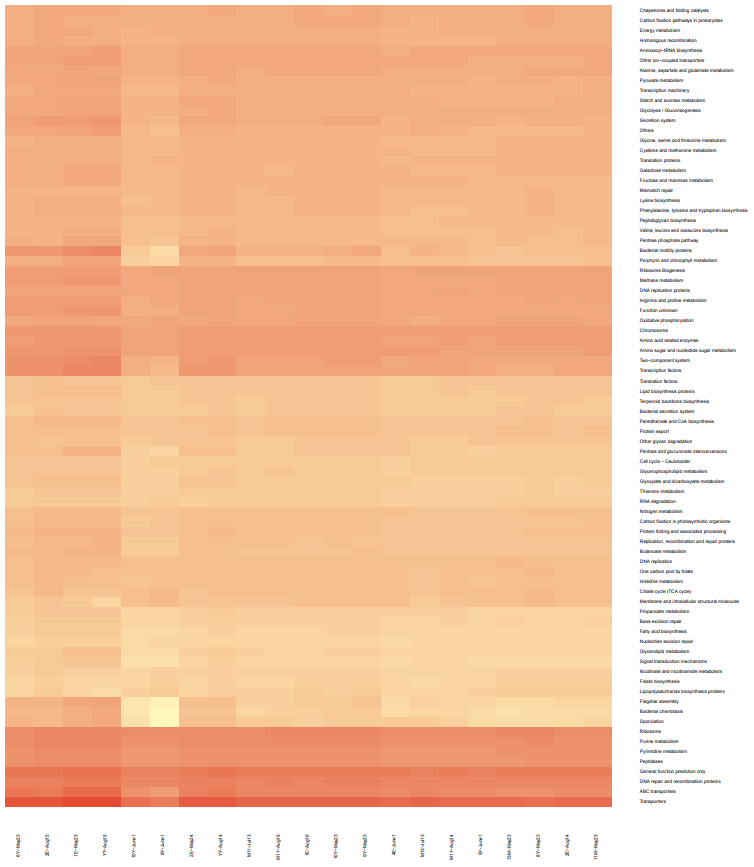


Figure: heatmap view of top 80 pathways

**biom_add_metadata (format conversion)**

Introduction: Add metadata to a BIOM table.

Input: txt/tsv file contains information of sample (in text format, qiime map file please see http://qiime.org/documentation/file_formats.html) and OTU table (in biom format).

Output: A biom file added metadata information (in biom format).

Test inputs:

input_metadata_txt (txt/tsv)

imcas:/test_kira/biom/map.tsv

input_otu_table (biom)

imcas:/test_kira/biom/otus.biom

Test example:

?.biom 1.01MB

**biom2txt & txt2biom (format conversion)**

Introduction: biom file <=> text file.

Input & Output: biom file & text file.

Test inputs:

imcas:/test_kira/biom/otus.biom

imcas:/test_kira/biom/otus.txt

Test arguments:

args_header_key (The observation metadata to include from the input BIOM table file when creating a tsv table file, by default no observation metadata will be included, string, [null]).

args_output_formate (enum=to-json|to-hdf5|[to-tsv]).

**Musket (error correction)**

Introduction: Musket is a well-established leading next-generation sequencing read error correction algorithm targetting Illumina sequencing.

Input: Error-existing reads (in fasta or fastq format)

Output: Error-pruned reads (in fasta or fastq format)

Test inputs:

input_seq1 (fastq/fasta)

imcas:/test_kira/soapec/yeast_test_1.fq

input_seq2 (fastq/fasta)

imcas:/test_kira/soapec/yeast_test_2.fq

Test arguments:

args_number_threads : number of threads (integer=2~16, [2]). 4

args_inorder: keep sequences outputed in the same order with the input (flag, [TRUE]).

args_k_mer_size: specify two paramters: k-mer size (integer=1~100, [21]). 21

args_genome_size: estimated total number of k-mers for this k-mer size. This value just balances the memory consumption between Bloom filters and hash tables ( integer = 1~1000M). 12000000

Test example:

?.fq 70.14MB

?.fq 70.19MB

**DIAMOND (ultra-fast alignment)**

Introduction: DIAMOND is a new high-throughput program for aligning DNA reads or protein sequences against a protein reference database.

Input: Reference sequences and query sequences (in fasta format).

Output: All the HSPs (in txt format).

Test inputs:

input_reference_fasta (fasta/fa/fna/fas)

imcas:/[test_kira](javascript:;)/blast/reference_prot.fas **rice protein database**

input_query_sequence (fasta/fa/fna/fas)

imcas:/test_kira/blast/query_prot.fas **test1**

imcas:/[test_kira](javascript:;)/Lactobacillus.fasta **test2**

Test arguments:

args_threads (integer, default=1~16, [NULL])

args_command (enum=blastp|[blastx]). **blastp in test1**

args_outfmt (output format, 0=BLAST pairwise, 5=BLAST XML, 6=BLAST tabular, 100=DIAMOND alignment archive (DAA), 101=SAM, enum = 0|5|[6]|100|101)

args_evalue (float, [null])

Test example:

?.txt 3.45KB **test1**

?.txt 4.43KB **test2**


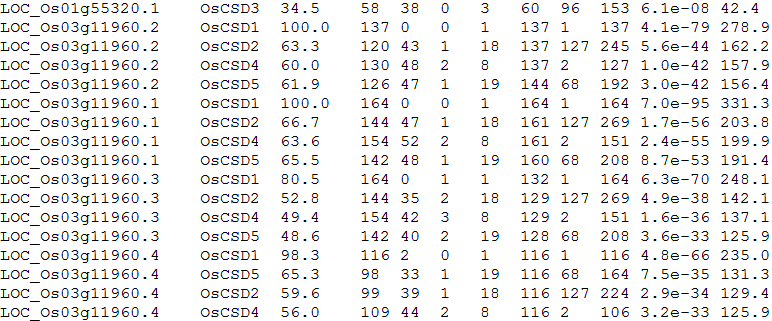


Figure: query_prot.fas alignment result in fmt=6

Supplementary:

BLAST output format (fmt=6):

Column 1: Query label;

Column 2: Target (database sequnece or cluster centroid) label;

Column 3: Percent identity;

Column 4: Alignment length;

Column 5: Number of mismatches;

Column 6&7: Start position and end position in query;

Column 8&9: Start position and end position in target;

Column 10: E-value calculated using Karlin-Altschul statistics;

Column 11: Bit score calculated using Karlin-Altschul statistics.

**LEfSe (metagenome effect factor analysis)**

Introduction: LEfSe (Linear discriminant analysis Effect Size) determines the features (organisms, clades, operational taxonomic units, genes, or functions) most likely to explain differences between classes by coupling standard tests for statistical significance with additional tests encoding biological consistency and effect relevance.

Input: OTU file which separate the classification level by “|” (in text format).

Output: Analysis results (in txt format), bar plot and cladogram plot (in pdf format)

Test inputs:

input_file (txt/csv)

imcas:/test_kira/lefse/lefse.csv

Test arguments:

args_row_class_1 (set which feature use as class, int =1~1000, [1]) 1

args_row_subclass_1 (set which feature use as subclass, -1 means no subclass, int =-1~1000, [-1]) **2**

args_row_subject_1 (set which feature use as subject, -1 means no subject, int =-1~1000, [-1]) **3**

args_whether_features_1 (set whether the features are on rows or on columns, enum=c|[r])

args_normalization_value_1 (set the normalization value, float, [1.0]). **1000000.0**

args_stratege_muti_class_2 (set the multiple testing correction options, enum=[0]|1|2) 0

args_Wilcoxon_test_2 (set the alpha value for the Wilcoxon test, enum=0.1|[0.05]|0.01) 0.05

args_one_against_one_2 (for multiclass tasks, set whether the test is performed in a more strict mode, **1 stands for more strict!! Default setting in LEfSe website is "All-against-all (more strict)"**, enum=[0]|1) 0

args_threshold_absolute_value_2 (set the threshold on the absolute value of the logarithmic LDA score, float, [2.0]) 2.0

args_same_name_2 (set whether perform the wilcoxon test only among the subclasses with the same name, int, [0]) 0

args_Anova_test_2 (set the alpha value for the Anova test, enum=0.1|[0.05]|0.01) 0.05

args_bar_subclades_3 (number of label levels to be displayed, int=-1~1000, [1]) 1

args_ dpi (dpi for the plot, enum=72|[150]|300|600|1200) 150

args_feature_num_5 (set the number of the feature to plot, int=0~100000)

args_feature_name_5 (set the name of the feature to plot, levels separated by ".", string, [NULL])

Test example:

?.pdf 18.60KB **barplot**

?.pdf 363.73KB **cladogram**

?.txt 100.72KB

*.tgz 230.23KB **features plot** (a zip file containing several features plot)


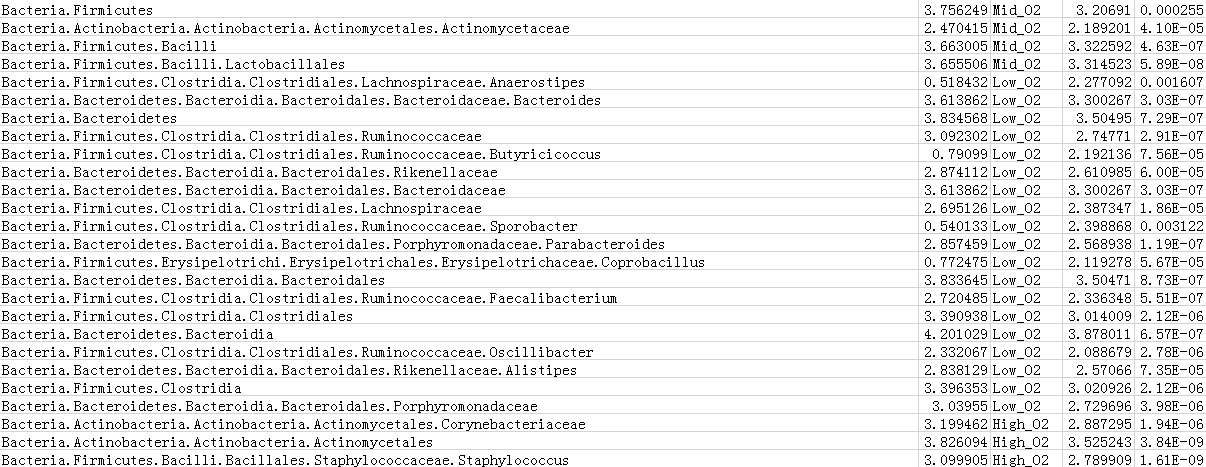


Figure: LEfSe text output, feature | log average | class | LDA effect size | p-value


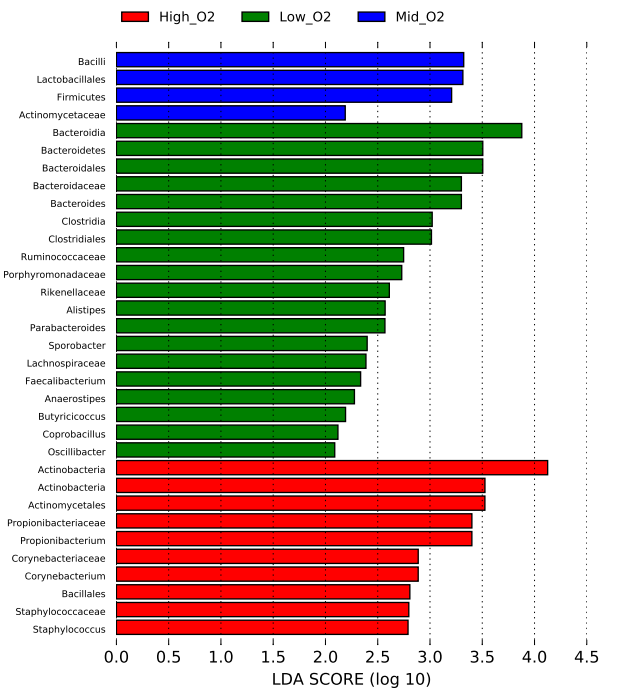


Figure: LDA score of all the bio-markers


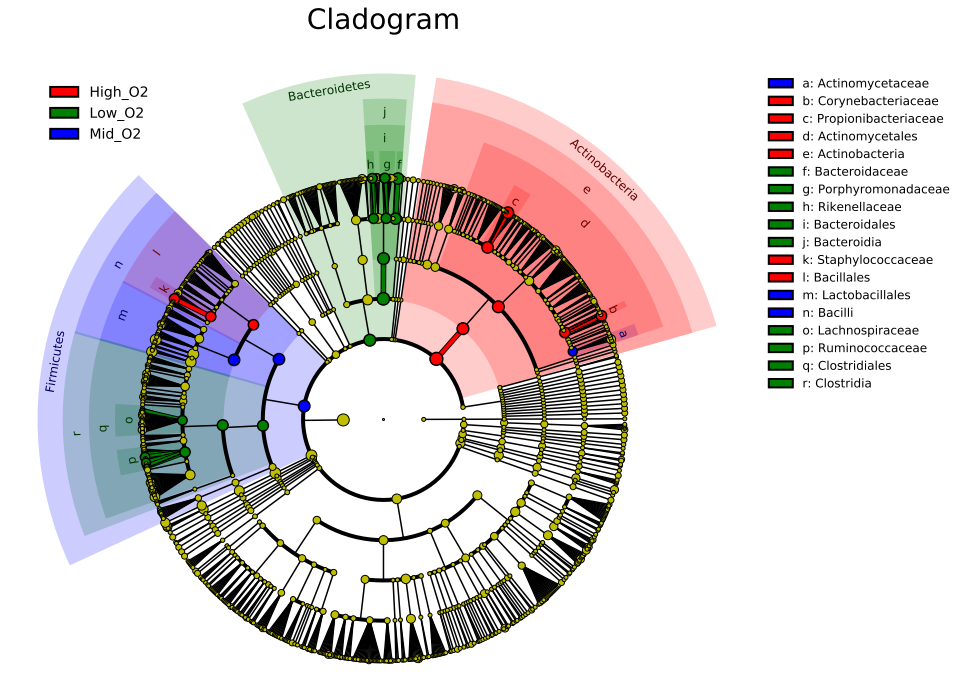


Figure: cladogram shows the effect of all the bio-markers


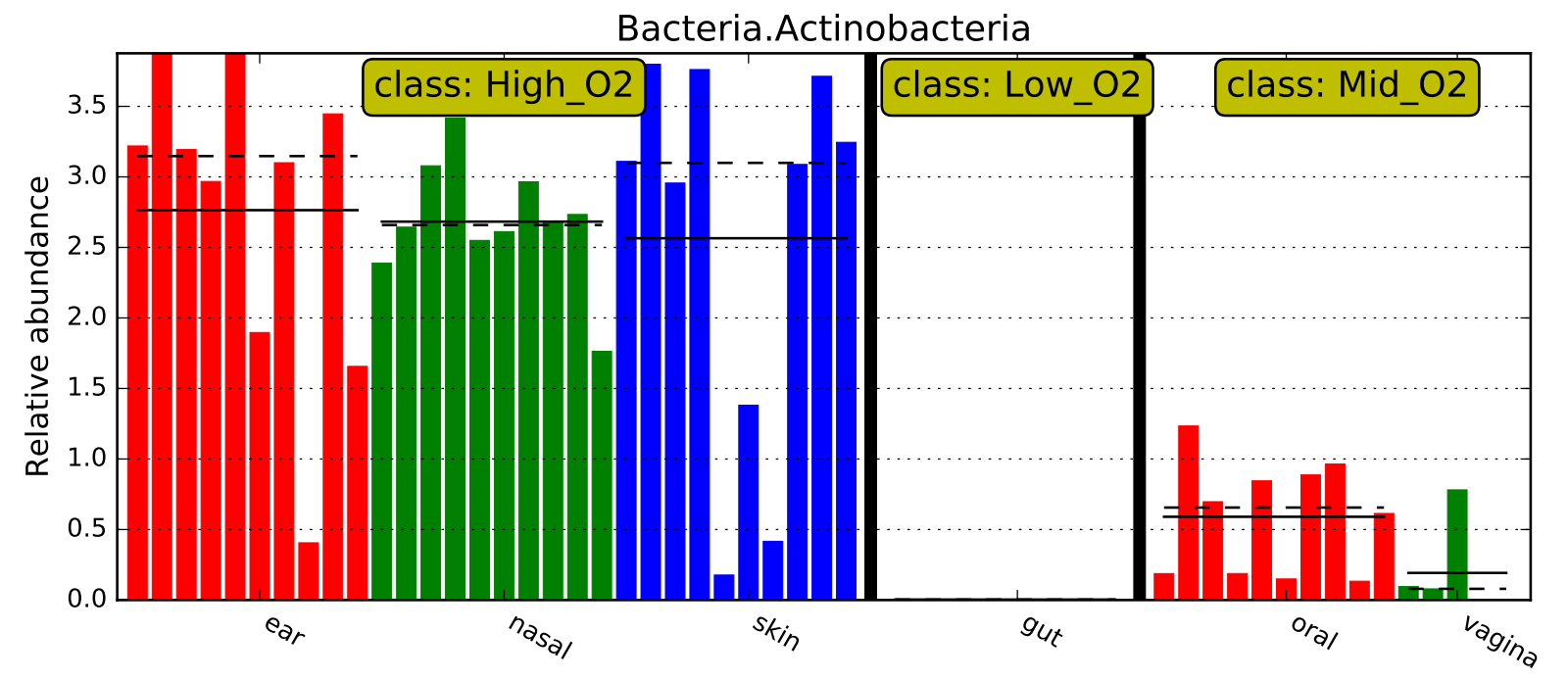


Figure: differential features of mainclass and subclass

**CD-HIT (protein cluster)**

Introduction: CD-HIT stands for Cluster Database at High Identity with Tolerance. The program (cd-hit) takes a fasta format sequence database as input and produces a set of 'non-redundant' (nr) representative sequences as output. In addition cd-hit outputs a cluster file, documenting the sequence 'groupies' for each nr sequence representative.

Input: Protein or nt sequences (in fasta format).

Output: 'non-redundant' (nr) representative sequences and a cluster file (in a zip file).

Test inputs:

input_seq (fa/fas/fasta)

imcas:/test_kira/cdhit/test-cdhit.fas

Test arguments:

args_identity_threshold (identical amino acids divided by the full length of the shorter sequence, float, [0.9])

args_threads (number of threads, integer, [1])

args_word_length (see http://www.bioinformatics.org/cd-hit/cd-hit-user-guide.pdf, integer, [5])

**PILER-CR (CRISPR element detection)**

Introduction: PILER-CR is public domain software for finding CRISPR repeats.

Input: DNA sequences (in fasta format).

Output: Direct repeat sequence, CRISPR arrays structure (in text format).

Test inputs:

input_seq (fasta/fa/fna)

imcas:/test_kira/Lactobacillus.fasta

Test example:

?.txt 7.86KB

?.fasta 120.00B


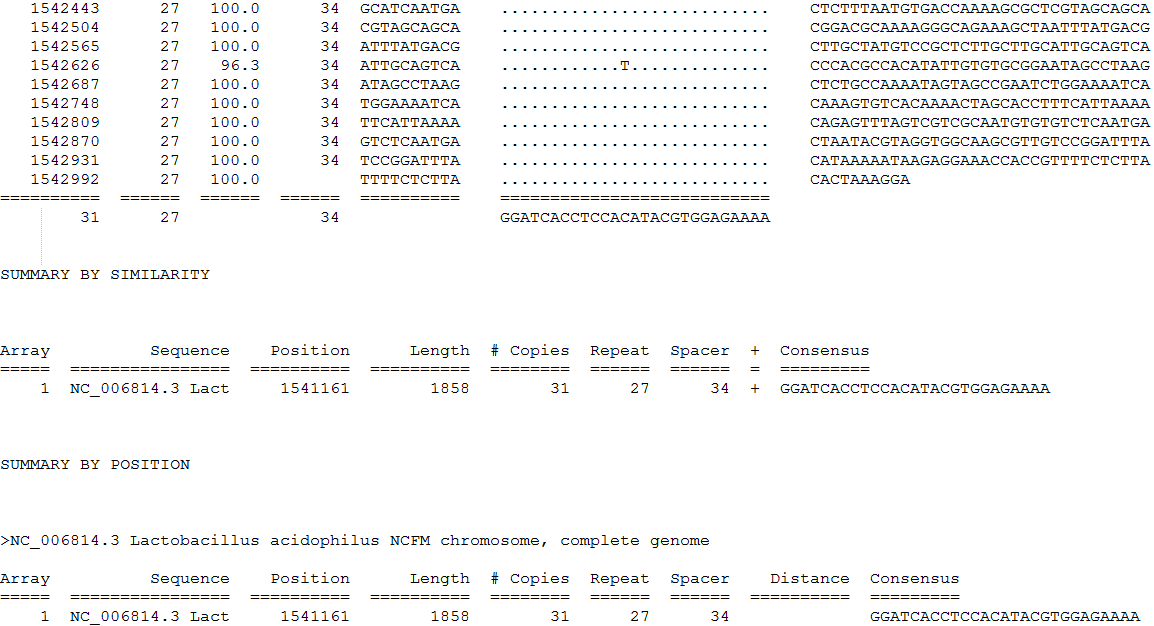


Figure: CRISPR arrays structure

**Cufflinks (transcript assembly and abundance estimation)**

Introduction: Cufflinks assembles transcripts, estimates their abundances, and tests for differential expression and regulation in RNA-Seq samples. It accepts aligned RNA-Seq reads and assembles the alignments into a parsimonious set of transcripts. Cufflinks then estimates the relative abundances of these transcripts based on how many reads support each one, taking into account biases in library preparation protocols.

Input: Output file named ‘accepted_hits.bam’ from tophat2 and gtf/gff file of the reference genome (optional, in fasta format).

Output: Assembled transcript and expression level file (zip file including gff file and FPKM file)

Test inputs:

input_bam (bam)

imcas:/test_kira/cufflinks/accepted_hits.bam

Test arguments:

args_threads (integer, [1])

Test example:

?.tar.gz 559.00B

unzip it, 4 files included: skipped.gtf, transcripts.gtf, genes.fpkm_tracking, isoforms. fpkm_tracking.

**VirFinder (metagenome virus recognition)**

Introduction: R package for identifying viral sequences from metagenomic data using sequence signatures.

Input: Sequence file (in fasta format).

Output: Matching results (in txt format). Each row represents a contig/sequences, with name, length, score, p-value and q-value. The higher score or lower p-value indicate higher likelihood of being a viral sequence. The q-value measures the proportion of false positives incurring when predicting viral sequences using the corresponding p-value as a threshold.

Test inputs:

input_reference (fasta/fa/fna)

imcas:/[test_kira](javascript:;)/virfinder/contigs.fa

Test example:

?.txt 2.32KB

**VirHostMatcher (metagenome virus recognition)**

Introduction: Matching hosts of viruses based on oligonucleotide frequency (ONF) comparison.

Input: Taxonomy of the hosts (in text format), virus and corresponding host sequences database (in fasta format).

Output: Matching results (zip file including report and interactive interface)

Test inputs:

input_vir_fasta (fasta/fa/fna)

/[test_kira](javascript:;)/VirHostMatcher/vir­_fasta/*

input_host_fasta (fasta/fa/fna)

/[test_kira](javascript:;)/VirHostMatcher/host_fasta/*

input_host_taxa (txt)

/[test_kira](javascript:;)/VirHostMatcher/[hostTaxa.txt](javascript:;)

Test arguments:

args_d2star (compute only d2star dissimilarity, 1 for yes, 0 for no, enum=[0]|1) 0

Test example:

?.tgz 24.20MB

unzip it, open html file:


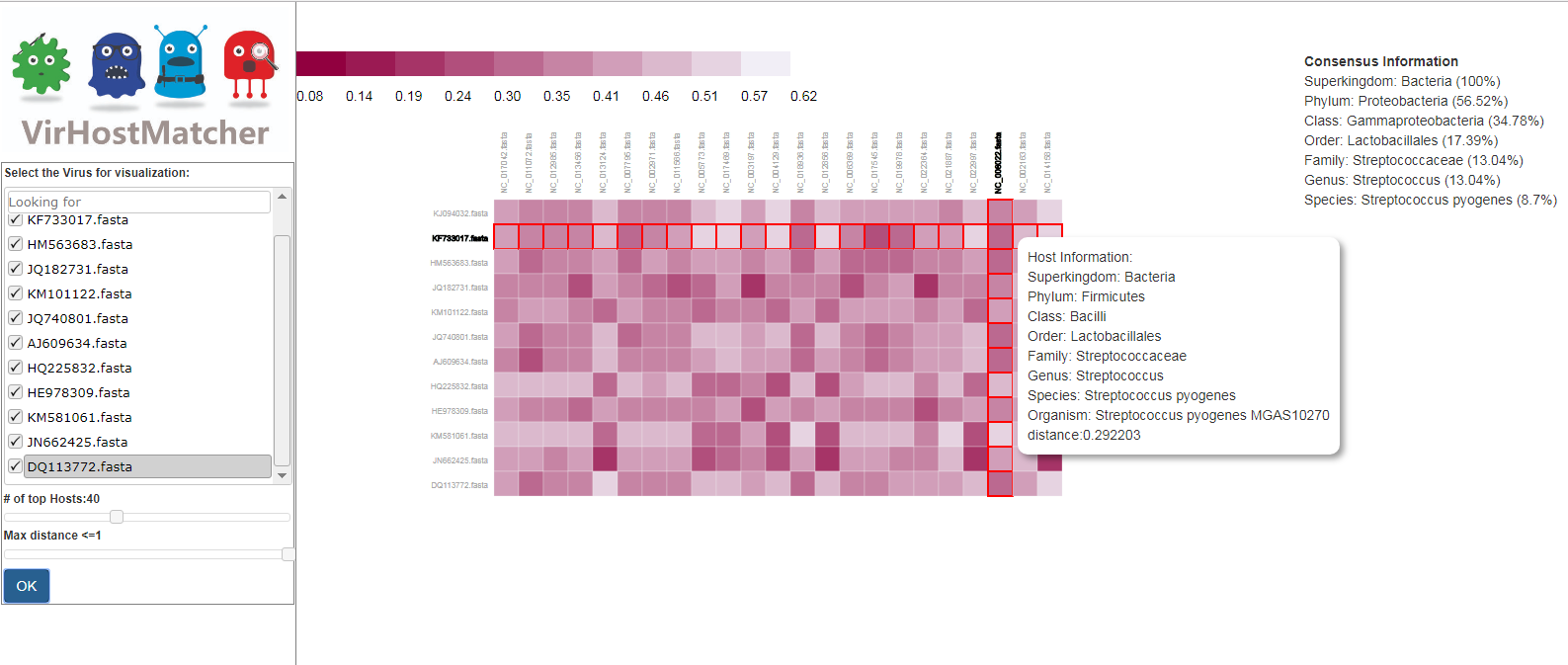


Figure: interactive interface of matching result

**PCoA plot (R script)**

Input: data matrix (tab-separated value, in txt format)

Test inputs:

input_data (txt)

imcas:/test_kira/nm.4272-S2.txt

Test arguments:

skippedline (first N rows will be skipped as marker, integer, [1]) **4**

topvector (first N rows sorted by average abundance will be calculated, integer, [80]) 80

datamulti (data will be multiple by N, integer, [1]) 1

markercol (color marker, see below, integer, [0]) **3**

markershape (shape marker, see below, integer, [0]) **2**

markeralpha (integer, [0]) 0

markersize (integer, [0]) 0

markerlabel (integer, [0]) 0

vegmethod (string=mahalanobis, cao, chao, binomial, raup, mountford, horn, morisita, altGower, gower, jaccard, kulczynski, canberra, manhattan, euclidean, bray, ... , [bray])

Test example:


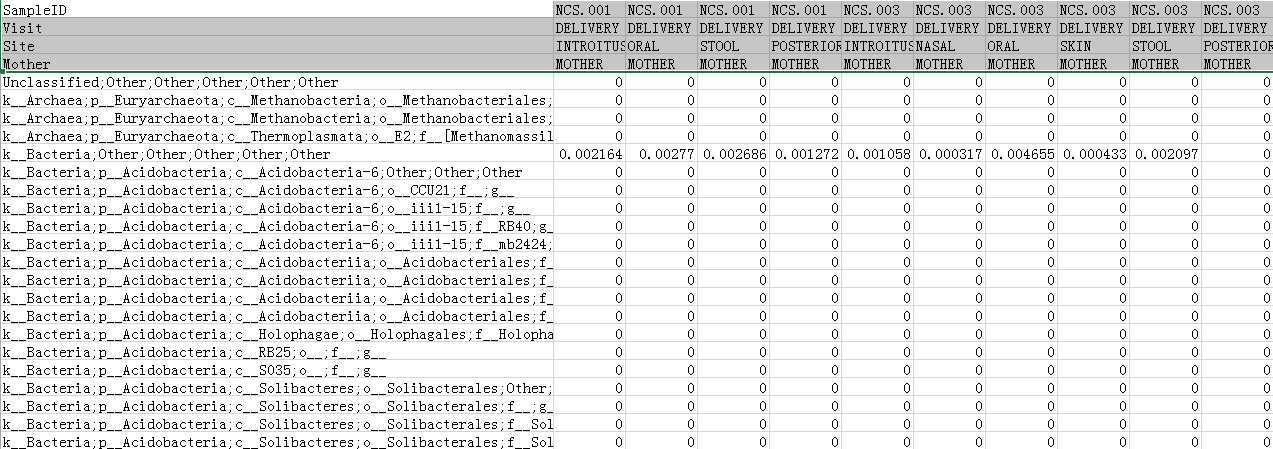


Figure: input data


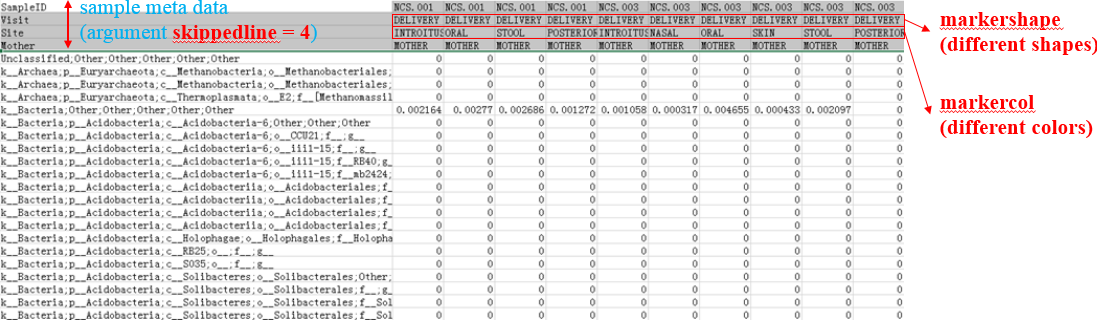


Figure: arguments explanation


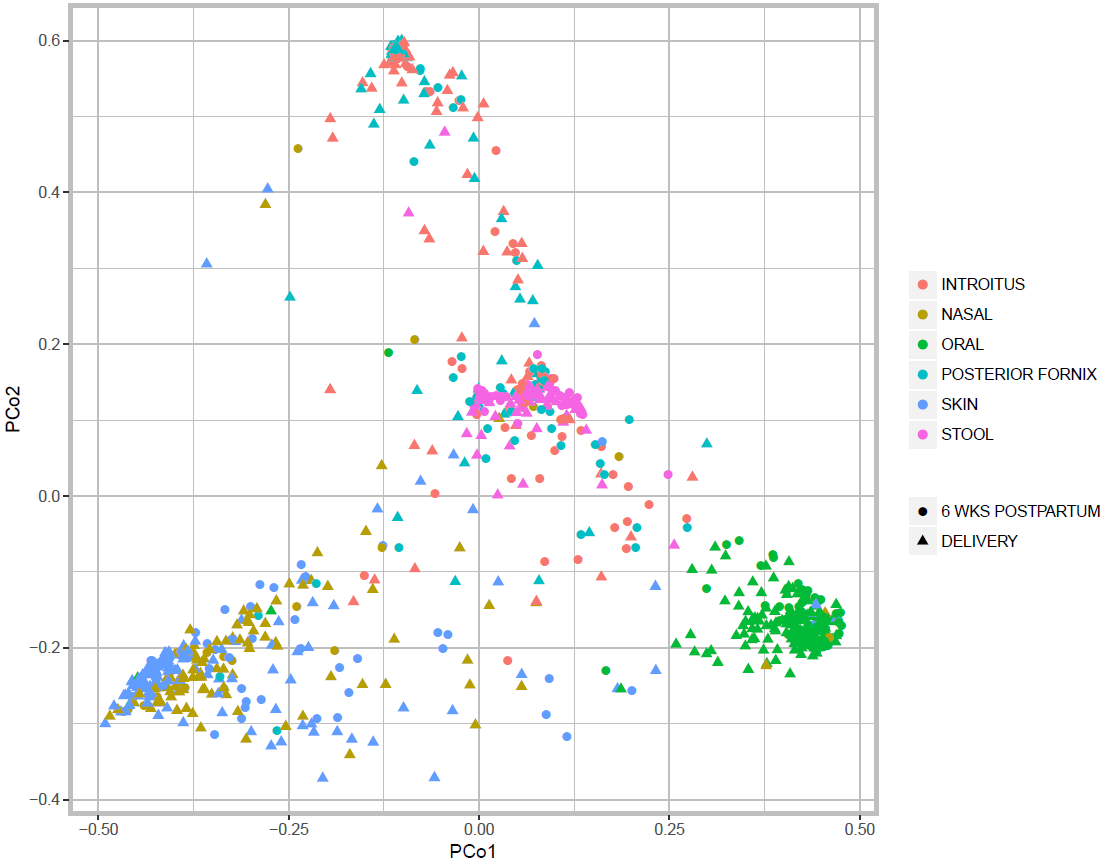


Figure: PCoA plot output

**Heatmap plot (R script)**

Input: data matrix (tab-separated value, in txt format)

Test inputs:

input_data (txt)

imcas:/test_kira/nm.4272-S2.txt

Test arguments:

skippedline (integer, [1]) **4**

topvector (integer, [80]) 80

colclust (integer=0~1, [1]) 1

rowclust (integer=0~1, [1]) 1

marker0 (integer, [1]) **2**

tranverse (integer=0~1, [0]) **1**

datafunc (enum=none|sqrt|log|[log10]) log10

datamulti (integer, [1]) **100000**

highcol (color, [#e34a33]) #e34a33

lowcol (color, [#fff7bc]) #fff7bc

midcol (color, [NULL])

Test example:


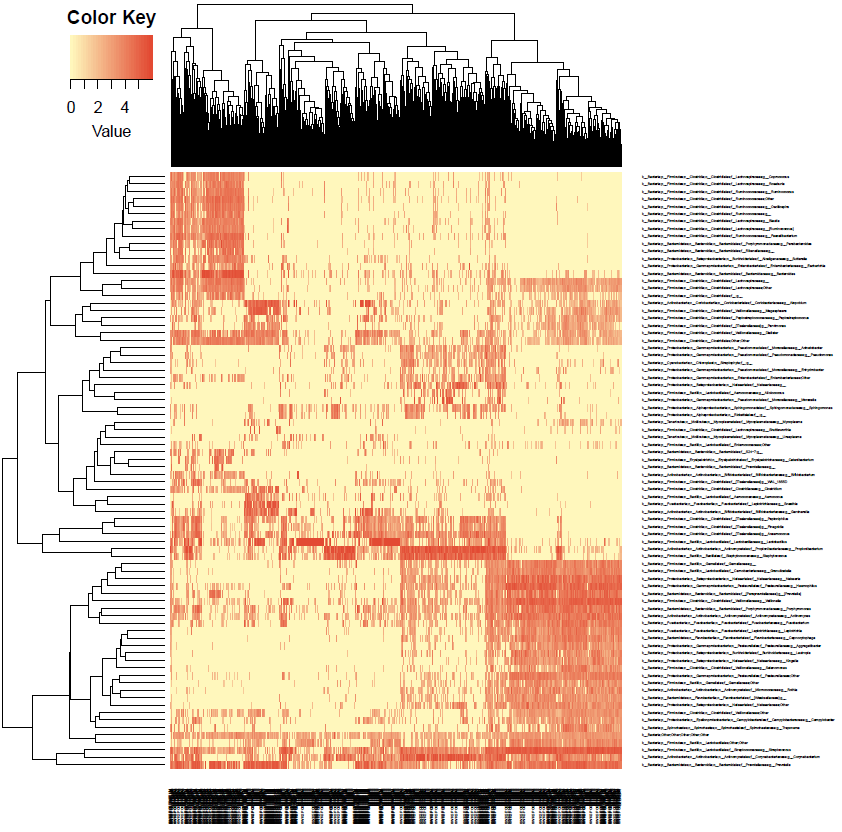


Figure: heatmap output

**box plot / violin plot (R script)**

Input: data matrix (tab-separated value, in txt format)

Test inputs:

input_data (txt)

imcas:/test_kira/huge_data.txt

Test arguments:

skippedcolumn (integer, [3]) **4**

datamulti (integer, [1]) 1

datafunc (enum=none|sqrt|log|[log10]) none

markercol (integer, [1]) **3**

markerfacet (integer, [2]) **4**

markerx (integer, [3]) **2**

outlier (display outlier, integer=0~1, [1]) 1

Test example:


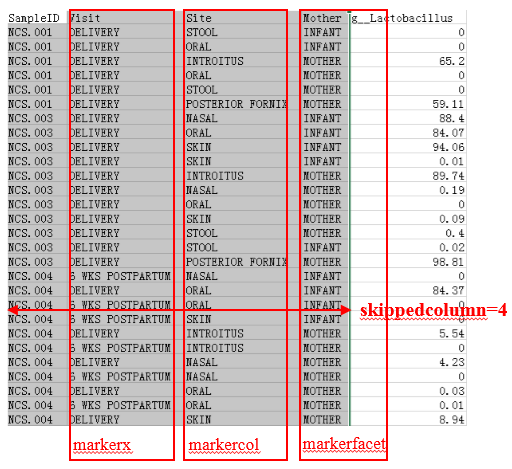


Figure: input data and arguments explanation


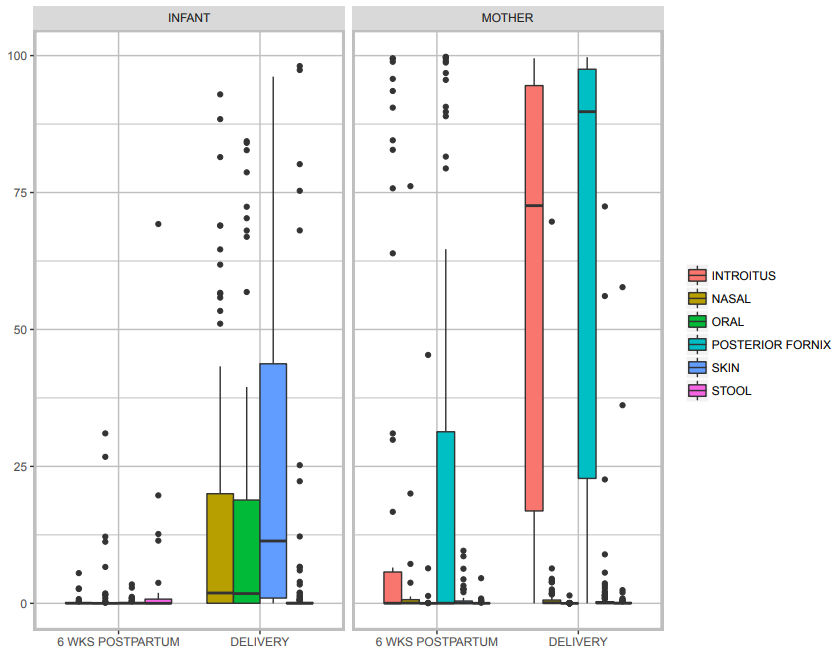


Figure: boxplot output

**bar plot (R script)**

Input: data matrix (tab-separated value, in txt format)

Test inputs:

input_data (txt)

imcas:/test_kira/barplot_data.txt

Test arguments:

skippedline (integer, [1]) 1

datamulti (integer, [1]) 1

datafunc (enum=none|sqrt|log|[log10]) none

markercol (enum=[row]|col) row

marker0 (integer, [1]) 1

type (enum=[stack]|dodge) stack

Test example:


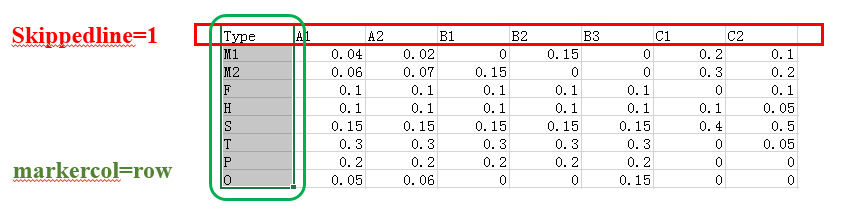


Figure: input data and arguments explanation


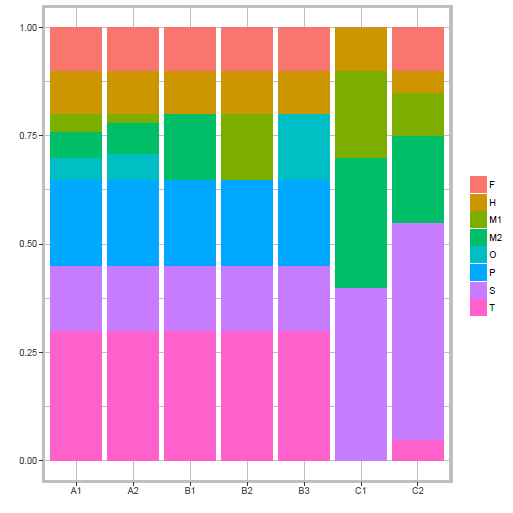

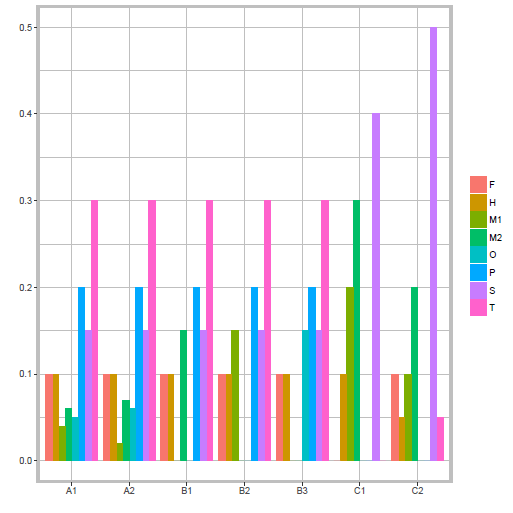


Figure: barplot output (stack vs. dodge)

**point plot (R script)**

Input: data matrix (tab-separated value, in txt format)

Test inputs:

input_data (txt)

imcas:/test_kira/point_data.txt

Test arguments:

skippedline (integer, [1]) 1

markerx (integer, [1]) 1

markery (integer, [2]) 2

markercol (integer, [3]) 3

markershape (integer, [4]) 4

markeralpha (integer, [5]) 5

markersize (integer, [6]) 6

Test example:


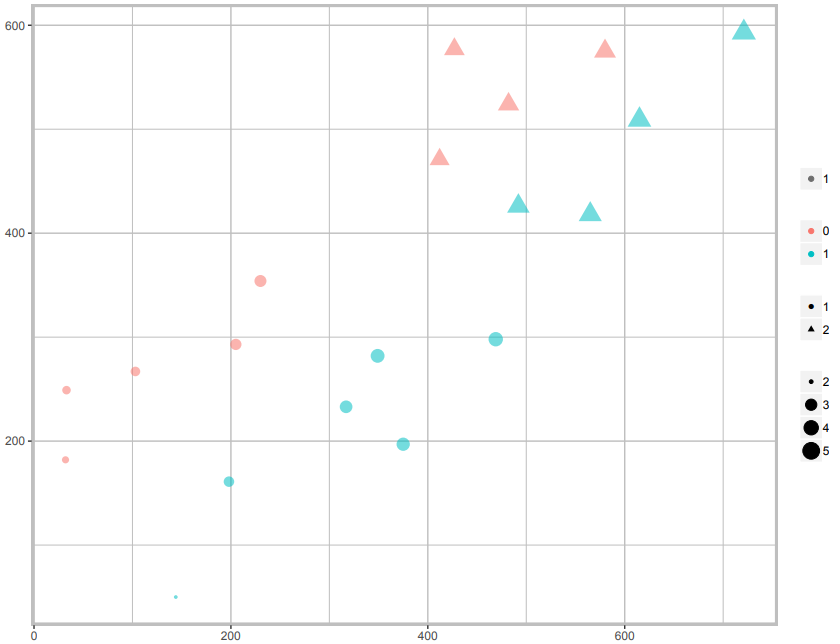


Figure: pointplot output

**DustMasker (quality control)**

Introduction: DustMasker is a program that identifies and masks out low complexity parts of a genome using a new and improved DUST algorithm. The main advantages of the new algorithm are symmetry with respect to taking reverse complements, context insensitivity, and much better performance.

Input: DustMasker takes its input as a FASTA formatted file containing one or more nucleotide sequences (fasta or blastdb).

Output: File that identified and masked out low complexity parts of a genome.

Test inputs:

genome_fasta (fasta/fna/fa/blastdb)

imcas:/test_kira/OrthoANI/Staphylococcus_aureus.fa

Test arguments:

args_window (DUST window length, integer=1~1000, [64])

args_level (DUST level, score threshold for sub windows, integer =1~150, [20])

Test example:

*.txt 32.39KB


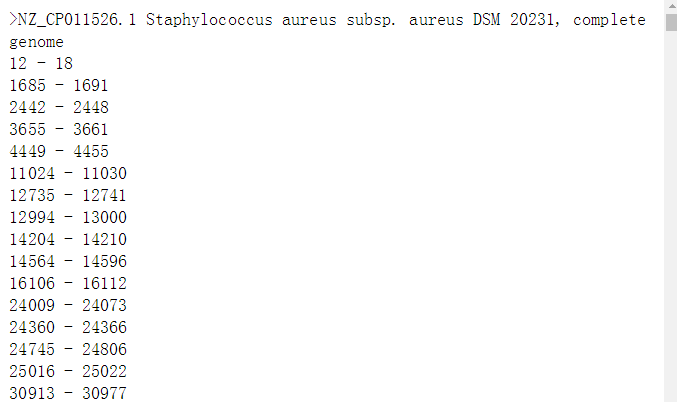


Figure: DustMasker results

**sickle (quality control)**

Introduction: Sickle is a tool that uses sliding windows along with quality and length thresholds to determine when quality is sufficiently low to trim the 3'-end of reads and also determines when the quality is sufficiently high enough to trim the 5'-end of reads.

Input: Raw reads data in fastq format.

Output: Trimed fastq file.

Test inputs:

input_fastq1 (fastq/fq)

imcas:/test_kira/StrainEst/reads1.fastq

input_fastq2 (fastq/fq)

imcas:/test_kira/StrainEst/reads2.fastq

Test arguments:

args_qual_type (Type of quality values, solexa: CASAVA < 1.3, illumina: CASAVA 1.3 to 1.7, sanger: which is CASAVA >= 1.8, enum=solexa|illumina|[sanger])

args_qual_threshold (Threshold for trimming based on average quality in a window (int=1~40, [20]).

args_length_threshold (Threshold to keep a read based on length after trimming, integer=1~100, [20])

args_no_fiveprime (Don't do five prime trimming, flag, [FALSE]).

args_truncate_n (Truncate sequences at position of first N, flag, [FALSE]).

Test example:

?.tgz 11.82MB

?.tgz 12.03MB

**StrainEst (strain profiling)**

Introduction: StrainEst is a novel, reference-based method that uses the Single Nucleotide Variants (SNV) profiles of the available genomes of selected species to determine the number and identity of coexisting strains and their relative abundances in mixed metagenomic samples.

Input: alignment file including bam, bai, and snp_clust.dgrp (snp database) file.

Output: estimated strain abundance.

Test inputs:

input_bai (bai)

imcas:/test_kira/StrainEst/reads.sorted.bai

input_database (dgrp)

imcas:/test_kira/StrainEst/P_acnes/snp_clust.dgrp

input_bam (bam)

imcas:/test_kira/StrainEst/reads.sorted.bam

Test example:

?.tgz 405.50KB

Unzip it, 4 txt files and a pdf file are included:

abund.txt: the predicted abundances for each reference genome;

max_ident.txt: for each reference genome, the percentage of alleles that are present in the metagenome;

info.txt: information about the prediction, including the prediction Pearson R;

counts.txt: number of counts for each SNV position/base pairs;

mse.pdf: Lasso cross-validation plot as a function of the shrinkage coefficient.


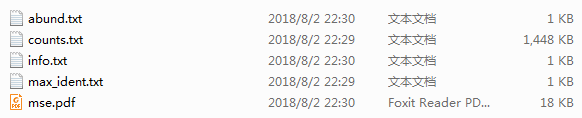


Figure: StrainEst results


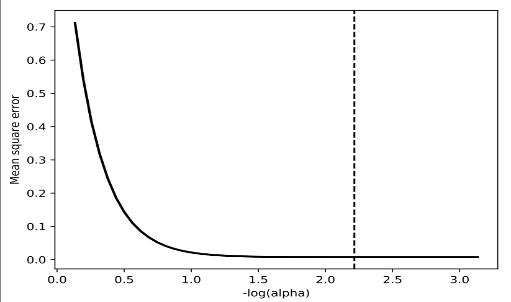


Figure: StrainEst Lasso cross-validation plot results

**Trinity (RNA *de novo* assembly)**

Introduction: Trinity, developed at the Broad Institute and the Hebrew University of Jerusalem, represents a novel method for the efficient and robust de novo reconstruction of transcriptomes from RNA-seq data. Trinity combines three independent software modules: Inchworm, Chrysalis, and Butterfly, applied sequentially to process large volumes of RNA-seq reads.

Input: pair-end reads in fastq format.

Output:

When Trinity completes, it will create a "Trinity.fasta" output file in the "trinity_out_dir/" output directory. Trinity groups transcripts into clusters based on shared sequence content. Such a transcript cluster is very loosely referred to as a "gene". This information is encoded in the Trinity fasta accession.

Test inputs:

Input_seq1 (fastq/fq/gz)

imcas:/test_kira/Trinity/reads.left.fq.gz

Input_seq2 (fastq/fq/gz)

imcas:/test_kira/Trinity/reads.right.fq.gz

Test arguments:

args_seqtype (type of reads, enum=fa|[fq]) fq

args_max_memory (suggested max memory to use by Trinity where limiting can be enabled, string, [10G]) 10G

args_jaccard_clip (option, set if you have paired reads and you expect high gene density with UTR overlap, use FASTQ input file format for reads, flag, [False]) False

args_CPU (number of CPUs to use, integer, [2]) 2

Test example:

?.tgz 14.04 MB

An example of the output file “Trinity.fasta”:

>TRINITY_DN23_c0_g1_i4 len=799 path=[1:0-324 4:325-325 5:326-326 10:327-798]

GAGAGGCTCCTGAAGGTGGAACCTAAAGCTGCCCAAGGCACTGCATTGTGTGAGCTTCAGGAGTTCAGAGGCAAAGCAGGTGACACTTCACTTACCATTTGGCTGTGTGAAATGACCCAGTCTAGGACAGGAAAGATATCTGCCTAGTATGGAGACTGAGACGAATGGTCAGGAATGGTTATCTGGTTGCAGCTTCTCTTTTTAAGTTTGGTTTAAATGCGTGACTATACTTAGCTATGAGGCAGGCTGATTCCTACCCAGGTAACACTAGGTTGTGGTGCTGCTGTTAACCGTGGTCTCTTCTGTTTCCCTTTCGCTGACCTCAGGCACCACACAGTCACTCGAGGCATCACCAAAGGTGTGAAGGAGGACTTCCGCCTGGCCATGGAGCGCCAGGTCTCCCGCTGTGGTGAGAACCTGATGGCGGTACTACATCGCTTCTGCATTAATGAGAAGATCTTGCTCCTGCAGACTCTGACCTGATAGACCTCCTGGCCACCAGCGGCTCAGCCATGACAAGCGCTGCCAGACCAGGGCTGATGAAGAAGAAAAACACCAAGTTCTAACTCCCTTAGTTGCTTAAAAGTCAATCCGAAGAGTATAGGAAGTTATTTCTATTTTTAAGACTTCAGGTTTTTTTAGTTTGTACAAAATAAGATTCAATCCATTTTGTAAATAAAAACCCGAAAGATTTGAAGTTTTAAAAAAAAACTTATTTTTAGTTTCAAGTAGGGTACATTTGTCTTCTATTTTTCTAATCATGGCAAAGGGGCAAAAATCCAACACAATTAAATGCAGG

Note: In the example above, the accession 'TRINITY_DN23_c0_g1_i4' indicates Trinity read cluster 'TRINITY_DN23_c0', gene 'g1', and isoform 'i4'. Because a given run of trinity involves many clusters of reads, each of which are assembled separately, and because the 'gene' numberings are unique within a given processed read cluster, the 'gene' identifier should be considered an aggregate of the read cluster and corresponding gene identifier, which in this case would be 'TRINITY_DN23_c0_g1'.

So, in summary, the above example corresponds to 'gene id: TRINITY_DN23_c0_g1' encoding 'isoform id: TRINITY_DN23_c0_g1_i4'.

The Path information stored in the header ("path=[1:0-324 4:325-325 5:326-326 10:327-798]") indicates the path traversed in the Trinity compacted de Bruijn graph to construct that transcript. In this case, node '1' corresponds to sequence range 0-324 of the transcript, and node 10 corresponds to sequence range 327-798 of the transcript sequence. The node numbers are unique only in the context of a given Trinity gene identifier, and so graph nodes can be compared among isoforms to identify unique and shared sequences of each isoform of a given gene.

**StringTie (RNA assembler)**

Introduction: StringTie is a fast and highly efficient assembler of RNA-Seq alignments into potential transcripts. It uses a novel network flow algorithm as well as an optional de novo assembly step to assemble and quantitate full-length transcripts representing multiple splice variants for each gene locus. Its input can include not only the alignments of raw reads used by other transcript assemblers, but also alignments longer sequences that have been assembled from those reads.

Input: a BAM file sorted by reference position and a reference annotation file in GTF/GFF format (Optional).

Note :A text file in SAM format which was produced by HISAT2 must be sorted and converted to BAM format using the samtools program (view and sort).

Output:

Main Outputs:

1. Stringtie's main output is a GTF file containing the assembled transcripts

2. Gene abundances in tab-delimited format (need to specify the parameters “-A”)

3. Fully covered transcripts that match the reference annotation, in GTF format (need to specify the parameters “-C” and input a GTF/GFF file)

4. Files (tables) required as input to Ballgown, which uses them to estimate differential 5. Expression (need to specify the parameters “-B”)

6. In merge mode, a merged GTF file from a set of GTF files

Test_input:

Input_bam (bam)

imcas:/test_kira/StringTie/yeast_sorted.bam

Input_gtf (gtf/gff3/gff/gff2)

imcas:/test_kira/StringTie/yeast.gff

Test arguments:

args_A (Gene abundances will be reported in tab delimited format in the output file with the given name, string, [gene_abundence.tab])

args_B (This switch enables the output of Ballgown input table files (*.ctab) containing coverage data for the reference transcripts given with the -G option, flag, [true])

args_C (StringTie outputs a file with the given name with all transcripts in the provided reference file that are fully covered by reads (requires -G), string, [cov_refs.gtf])

args_M (Sets the maximum fraction of muliple-location-mapped reads that are allowed to be present at a given locus, float = 0.0~1.0, [0.95])

args_f (Sets the minimum isoform abundance of the predicted transcripts as a fraction of the most abundant transcript assembled at a given locus, float=0.0~1.0, [0.1])

args_label (Sets <label> as the prefix for the name of the output transcripts, string, [STRG])

args_m (Sets the minimum length allowed for the predicted transcripts, integer, [200])

Test example:

?.tar.gz 1.02 MB

Unzip it, the file contains:

assembly.gtf, cov_refs.gtf, gene_abundence.tab, Ballgown_input.tgz


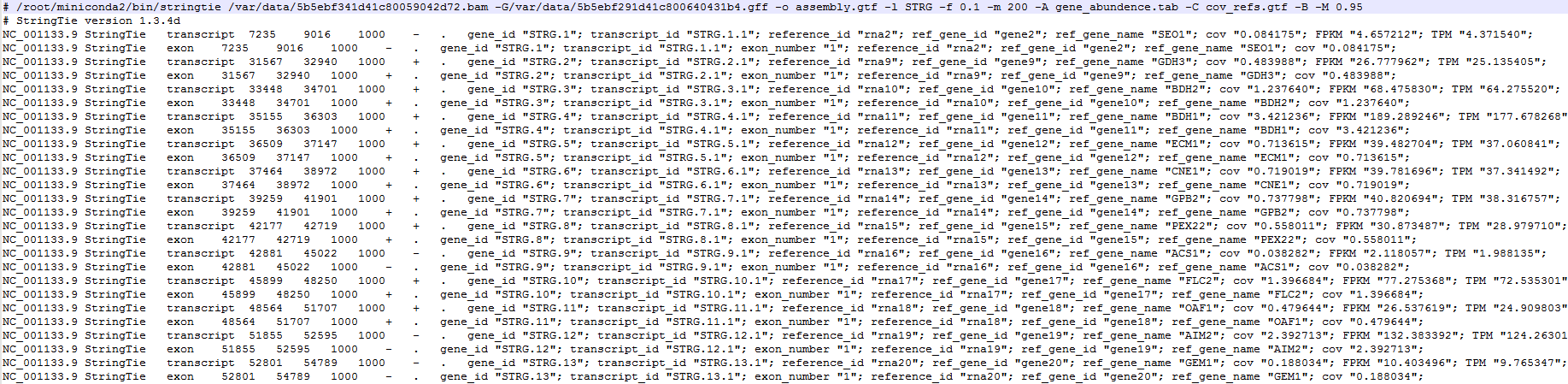


Figure: an example of a transcript assembled by StringTie (assembly.gtf file)

Colum1: seqname: Denotes the chromosome, contig, or scaffold for this transcript. Here the assembled transcript is on chromosome X.

Colum2: source: The source of the GTF file. Since this example was produced by StringTie, this column simply shows 'StringTie'.

Cloum3: start: Start position of the feature (exon, transcript, etc), using a 1-based index.

Cloum4: end: End position of the feature, using a 1-based index.

Colum5: score: A confidence score for the assembled transcript. Currently this field is not used, and StringTie reports a constant value of 1000 if the transcript has a connection to a read alignment bundle.

Colum6: strand: If the transcript resides on the forward strand, '+'. If the transcript resides on the reverse strand, '-'.

Cloum7: frame: Frame or phase of CDS features. StringTie does not use this field and simply records a ".".

Cloum8: attributes: A semicolon-separated list of tag-value pairs, providing additional information about each feature. Depending on whether an instance is a transcript or an exon and on whether the transcript matches the reference annotation file provided by the user, the content of the attributes field will differ. The following list describes the possible attributes shown in this column:

gene_id: A unique identifier for a single gene and its child transcript and exons based on the alignments' file name.

transcript_id: A unique identifier for a single transcript and its child exons based on the alignments' file name.

exon_number: A unique identifier for a single exon, starting from 1, within a given transcript.

reference_id: The transcript_id in the reference annotation (optional) that the instance matched.

ref_gene_id: The gene_id in the reference annotation (optional) that the instance matched.

ref_gene_name: The gene_name in the reference annotation (optional) that the instance matched.

cov: The average per-base coverage for the transcript or exon.

FPKM: Fragments per kilobase of transcript per million read pairs. This is the number of pairs of reads aligning to this feature, normalized by the total number of fragments sequenced (in millions) and the length of the transcript (in kilobases).

TPM: Transcripts per million. This is the number of transcripts from this particular gene normalized first by gene length, and then by sequencing depth (in millions) in the sample.

**STAR (spliced mapping)**

Introduction: STAR (Spliced Transcripts Alignment to a Reference) is an ultrafast universal RNA-seq aligner. It not only can perform unbiased de novo detection of canonical junctions, but also can discover non-canonical splices and chimeric (fusion) transcripts, and is capable of mapping full-length RNA sequences.

Input: genome reference sequence in fasta format, the file with annotated transcripts in the standard GTF format and pair-end reads in fastq format.

Output:

1. Unsorted or sorted-by-coordinate BAM file. (specify by “--outSAMtype” arguments )

2. Log.out: main log file with a lot of detailed information about the run. This file is most useful for troubleshooting and debugging.

3. Log.progress.out: reports job progress statistics, such as the number of processed reads, % of mapped reads etc. It is updated in 1 minute intervals.

4. Log.final.out: summary mapping statistics after mapping job is complete, very useful for quality control. The statistics are calculated for each read (single- or paired-end) and then summed or averaged over all reads. Note that STAR counts a paired-end read as one read, (unlike the samtools flagstat/idxstats, which count each mate separately). Most of the information is collected about the UNIQUE mappers (unlike samtools flagstat/idxstats which does not separate unique or multi-mappers). Each splicing is counted in the numbers of splices, which would correspond to summing the counts in SJ.out.tab. The mismatch/indel error rates are calculated on a per base basis, i.e. as total number of mismatches/indels in all unique mappers divided by the total number of mapped bases.

5. SJ.out.tab: contains high confidence collapsed splice junctions in tab-delimited format. Note that STAR defines the junction start/end as intronic bases, while many other software define them as exonic bases.

Test_input:

Input_ref (fasta/fas/fna/fa)

imcas:/test_kira/STAR/yeast.fas

Input_gtf (gff/gff2/gff3/gtf)

imcas:/test_kira/STAR/yeast.gff

Input_seq1 and Input_seq2 (fastq/fq)

imcas:/test_kira/STAR/s2_y_1.fq

imcas:/test_kira/STAR/s2_y_2.fq

Test arguments:

args_len (specifies the length of the genomic sequence around the annotated junction to be used in constructing the splice junctions database. Ideally, this length should be equal to the ReadLength - 1, integer, [100]) 75

args_outSAMtype (STAR can output alignments directly in binary BAM format, thus saving time on converting SAM files to BAM. It can also sort BAM files by coordinates, which is required by many downstream applications, enum=BAM Unsorted|[BAM SortedByCoordinate]|BAM Unsorted SortedByCoordinate) BAM SortedByCoordinate

**output sorted by coordinate Aligned.sortedByCoord.out.bam file, similar to samtools sort command**

Test example:

?.bam 12.4 MB

?Log.final.out 2 KB

?Log.out 20 KB

?Log.progress.out 1 KB

?SJ.out.tab 10 KB


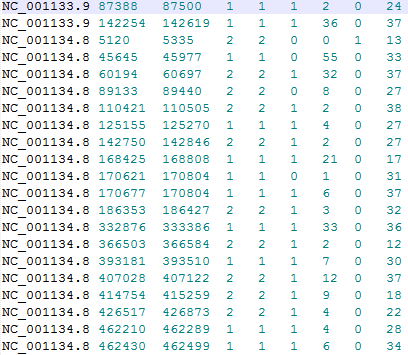


Figure: part of the SJ.out.tab file

Supplementary:

SJ.out.tab output format:

column 1: chromosome

column 2: first base of the intron (1-based)

column 3: last base of the intron (1-based)

column 4: strand (0: undefined, 1: +, 2: -)

column 5: intron motif: 0: non-canonical; 1: GT/AG, 2: CT/AC, 3: GC/AG, 4: CT/GC, 5:AT/AC, 6: GT/AT

column 6: 0: unannotated, 1: annotated (only if splice junctions database is used)

column 7: number of uniquely mapping reads crossing the junction

column 8: number of multi-mapping reads crossing the junction

column 9: maximum spliced alignment overhang

**PfamScan (Pfam annotation)**

Introduction: PfamScan is used to search a FASTA sequence against a library of Pfam HMM.

Input: A file containing a valid sequence in FASTA format can be used as input.

Output: A text format output file that contains the predicted domain of each sequence.

Test inputs:

input_fasta_file (fasta/fas)

imcas:/test_kira/pfamscan/test.fasta

Test arguments:

args_align (show the HMM-sequence alignment for each match, flag, [False]) False

args_evalue (specify hmmscan evalue sequence cutoff for Pfam-A searches, float, [1.0]) 1.0

args_overlap (show overlapping hits within clan member families, applies to Pfam-A families only, label, [False]) False

Test example:

?.txt 3.17KB


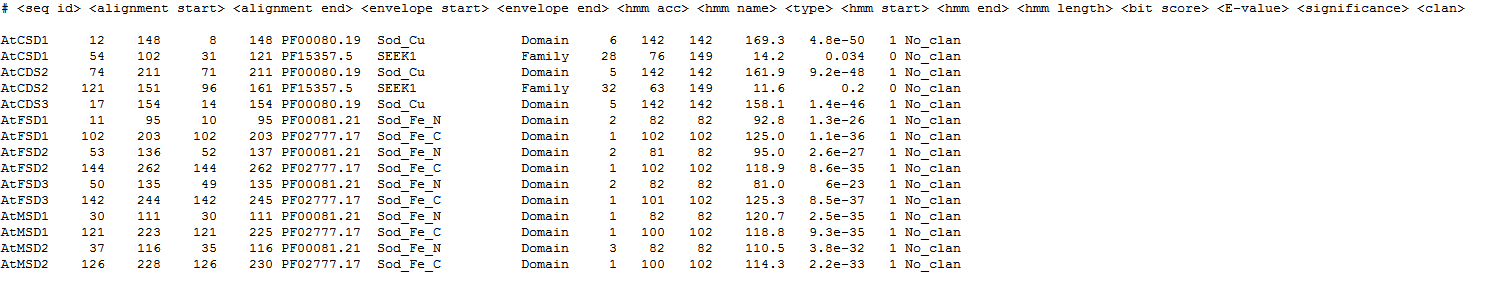


Figure: a txt output file of PfamScan

**Cutadapt (quality control)**

Introduction: Cutadapt finds and removes adapter sequences, primers, poly-A tails and other types of unwanted sequence from your high-throughput sequencing reads. Cleaning your data in this way is often required: Reads from small-RNA sequencing contain the 3' sequencing adapter because the read is longer than the molecule that is sequenced. Amplicon reads start with a primer sequence. Poly-A tails are useful for pulling out RNA from your sample, but often you don’t want them to be in your reads.

Input: pair-end reads

Output: pair-end reads with removed adapter

Test inputs:

input_seq1 and input_seq2 (fasta/fas/fa/fna/fastq/fq/gz/bz2/xz)

imcas:/test_kira/cutadapt/test_1.fasta

imcas:/test_kira/cutadapt/test_2.fasta

Test arguments:

args_a (Sequence of an adapter ligated to the 3' end (paired data: of the first read), The adapter and subsequent bases are trimmed. If a '$' character is appended ('anchoring'), the adapter is only found if it is a suffix of the read, string, specified by users)

args_A (3' adapter to be removed from second read in a pair, string, specified by users)

args_g (Sequence of an adapter ligated to the 5' end (paired data: of the first read). The adapter and any preceding bases are trimmed. Partial matches at the 5' end are allowed. If a '^' character is prepended ('anchoring'), the adapter is only found if it is a prefix of the read, string, specified by users) **CAGGTATATCGA**

args_G (5' adapter to be removed from second read in a pair, string, specified by users) **CAGGTATATCGA**

args_error (Maximum allowed error rate, no. of errors divided by the length of the matching region, float, [0.1]) 0.1

args_discard (Discard reads that contain an adapter. Also use -O to avoid discarding too many randomly matching reads, flag, [False]) False

args_O (Require MINLENGTH overlap between read and adapter for an adapter to be found, integer, [3]) 3

args_m (Discard reads shorter than LENGTH, integer, [0]) 0

args_pair_filter (Which of the reads in a paired-end read have to match the filtering criterion in order for the pair to be filtered, enum=[any]|both) any

Test example:

?.tgz 1.34 KB

Unzip it, this file contains two file which removed adapters. The format of these two file was the same as the input file.

**ART-illumina (simulator)**

Introduction: ART is a set of simulation tools to generate synthetic next-generation sequencing reads. ART simulates sequencing reads by mimicking real sequencing process with empirical error models or quality profiles summarized from large recalibrated sequencing data. ART can also simulate reads using user own read error model or quality profiles. ART supports simulation of single-end, paired-end/mate-pair reads of three major commercial next-generation sequencing platforms: Illumina's Solexa, Roche's 454 and Applied Biosystems' SOLiD.

Input: Genome reference sequence in fasta format.

Output: Simulated pair-end reads in fastq format.

Test inputs:

input_ref (fasta/fna/fas/fa format)

imcas:/test_kira/Lactobacillus.fasta

Test arguments:

args_f (the fold of read coverage to be simulated or number of reads/read pairs generated for each amplicon, integer, [20]) 20

args_l (the length of reads to be simulated, integer, [150]) 150

args_m (the mean size of DNA/RNA fragments for paired-end simulations, integer, [200]) 200

args_s: (the standard deviation of DNA/RNA fragment size for paired-end simulations, integer, [10]) 10

Test example:

?.fastq 41.27MB

?.fastq 41.27MB

**pIRS (simulator)**

Introduction: pIRS is a program for simulating Illumina PE reads, with a series of characters generated by Illumina sequencing platform, such as insert size distribution, sequencing error(substitution, insertion, deletion), quality score and GC content-coverage bias.

Input: Genome reference sequence in fasta format.

Output: Simulated pair-end reads in fastq format.

Test inputs:

input_ref (fasta/fna/fa/gz)

imcas:/test_kira/Lactobacillus.fasta

Test arguments:

args_l (read_len, set length of read, read1 and read2 have the same length, integer, [100])

args_x (coverage, set the sequencing coverage, sometimes called depth, integer, [5]) **50**

args_m (insertsize_mean, set the average value of insert size, integer, [500]) **600**

args_v (insertsize_sd, set the standard deviation of insert sizes, default is insertsize_mean/20, int)

args_e (substitution-error rate, set the average substitution-error rate (0 or 0.0001~0.63) over all cycles, default is average substitution-error rate of Base-calling profile, float)

args_a (simulate reads indel, 0 is no, 1 is yes, integer, [1])

args_g (simulate GC bias, 0 is no, 1 is yes, integer, [1])

args_f (cyclize insert fragment (influence on PE reads' direction) 0 is read1-forward read2-reverse, 1 is read1-reverse read2-forward, integer [0])

Test example:

?.fastq 105.89 MB

?.fastq 105.89 MB

**Glimmer (gene prediction)**

Introduction: Glimmer is a system for finding genes in microbial DNA, especially the genomes of bacteria, archaea, and viruses. Glimmer (Gene Locator and Interpolated Markov ModelER) uses interpolated Markov models (IMMs) to identify the coding regions and distinguish them from noncoding DNA.

Input: Genome in fasta format.

Output: Glimmer gene predict and detail report file

Test inputs:

input_genome (fasta/fa/fna)

imcas:/test_kira/OrthoANI/Staphylococcus_aureus.fa

Test arguments:

args_gene_len (Set minimum gene length to n, integer=0~10000000, [110])

args_max_olap (Set maximum overlap length to n, Overlaps this short or shorter are ignored, int=0~100000, [50])

args_threads (Set threshold score for calling as gene to n, If the in-frame score≥n, then the region is given a number and considered a potential gene, int=1~16, [16])

Test example:

?.txt 1.02MB **details**

?.txt 99.42KB **predict**


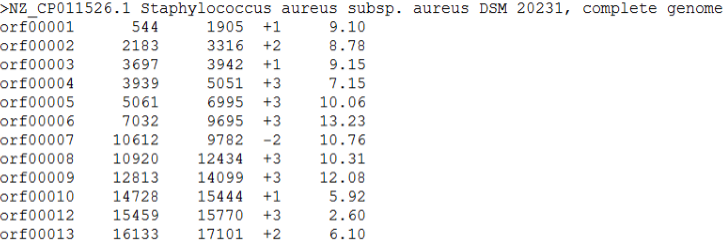


Figure: gene predict file


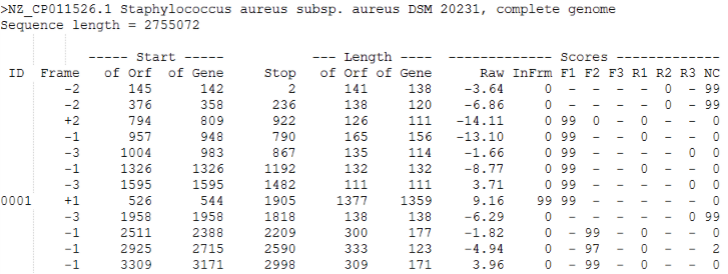


Figure: detail report file

Supplementary:

Glimmer predict file:

Column 1: The identifier of the predicted gene. The numeric portion matches the number in the ID column of the .detail file.

Column 2: The start position of the gene.

Column 3: The end position of the gene. This is the last base of the stop codon, i.e., it includes the stop codon.

Column 4: The reading frame.

Column 5: The per-base “raw” score of the gene. This is slightly different from the value in the .detail file, because it includes adjustments for the PWM and startcodon frequency.

The detailed information can be seen in http://ccb.jhu.edu/software/glimmer/ glim302notes.pdf

**mash (distance calculator)**

Introduction: Estimate the distance of each query sequence to the reference. And determine how well query sequences are contained within a pool of sequences.

Input: reference and queries in either fasta or fastq, and Mash sketch files (.msh) with matching k-mer sizes.

Output: distance calculation result.

Test inputs:

input_database_ref (msh)

imcas:/test_kira/mash_db/refseq.genomes.k21s1000.msh

input_seq (fasta/fa/fastq/fq/gz/fna)

imcas:/test_kira/mash_testdata/genome3.fna

imcas:/test_kira/mash_testdata/genome2.fna

imcas:/test_kira/mash_testdata/genome1.fna

Test arguments:

args_Maximum_distance (Maximum distance to report, float=0~1, [1.0])

args_Maximum_p_value (Maximum p-value to report, float=0~1,[1.0])

args_thread (int=1~16, [16])

args_Winner_takes_all (Winner-takes-all strategy for identity estimates. After counting hashes for each query, hashes that appear in multiple queries will be removed from all except the one with the best identity (ties broken by larger query), and other identities will be reduced. This removes output redundancy, providing a rough compositional outline, flag,[TURE])

Test example:

?.txt 20.95MB **#distance.tab**

?.txt 4.09MB **#screen.tab**


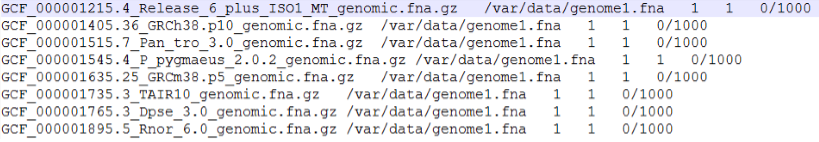


Figure: Distance.tab


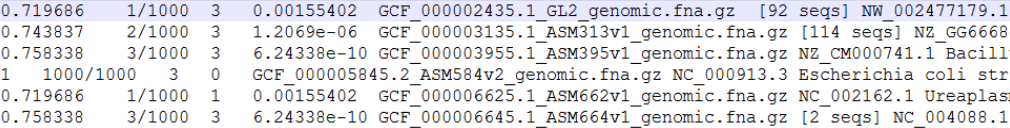


Figure: Screen.tab

Supplymentary:

Distance.tab: [reference-ID, query-ID, distance, p-value, shared-hashes].

Screen.tab: [identity, shared-hashes, median-multiplicity, p-value, query-ID, query-comment].

**OrthoANI (distance calculator)**

Introduction: Although ANI is widely used to classify and identify bacteria, OrthoANI ([Lee et al. 2015](http://ijs.microbiologyresearch.org/content/journal/ijsem/10.1099/ijsem.0.000760)) was developed to overcome the large differences in reciprocal ANI values associated with the ANI algorithm. Furthermore, OrthoANI tool employees USEARCH over BLAST for its OrthoANI calculations which increases the number of comparative studies and substantially decrease computational time.

Input: Genome WGS sequences in fasta format.

Output: ANI and GGDC value between two genomes.

Test inputs:

input_genome_seq1 & input_genome_seq2 (fasta/fa/fna):

imcas:/test_kira/OrthoANI/Staphylococcus_aureus.fa

imcas:/test_kira/OrthoANI/Bacillus_anthracis.fna

Test arguments:

args_num_threads (integer, default=1~16, [NULL])

Test example:

GGDC1: 98.7345

GGDC2: 15.0929

GGDC3: 98.5095

ANI: 66.5128

(Recommended Use GGDC2.The GGDC is a state-of-the-art in silico method for genome-to-genome comparison, thus reliably mimicking conventional DDH, except for its pitfalls.)

**BLASR (TGS reads mapping)**

Introduction: The method BLASR (Basic Local Alignment with Successive Refinement) was used to map Single Molecule Sequencing (SMS) reads that are thousands of bases long, with divergence between the read and genome dominated by insertion and deletion error. The method is benchmarked using both simulated reads and reads from a bacterial sequencing project.

Input:

Query sequence: long reads in bam, fasta or bax.h5 format

Reference sequence: fasta format

Output:

A specific format file (set by users) which contains the alignment information.

Test inputs:

input_query (bam/fasta/bax.h5/fofn)

imcas:/test_kira/blasr/pacbio.fasta

input_ref (fa/fasta)

imcas:/test_kira/blasr/Ecoli_genome.fasta

Test arguments:

args_clipping (Use no/hard/subread/soft clipping, ONLY for SAM/BAM output. enum =[none]|hard|subread|soft) none

args_maxscore (Maximum score to output, high is bad, negative is good, integer, [-200]) -200

args_minmatch (Minimum seed length. Higher minMatch will speed up alignment, but decrease sensitivity, integer, [12]) 12

args_minReadLength (Skip reads that have a full length less than l, Subreads may be shorter, integer, [50]) 50

args_outformat (Output format, enum={[--sam]|-m 0|-m 1|-m 2|-m 3|-m 4|-m 5) --sam

Test example:

?.tgz 65.45MB

Supplementary:

-m 0 format:

blasr like human-readable output with |'s connecting matched nucleotides.

-m 1 format:

Space-delimited summary of alignments containing 11 fields: qName tName qStrand tStrand score percentSimilarity tStart tEnd tLength qStart qEnd qLength nCells

-m 2 format:

XML format.

-m 3 format:

Vulgar format (deprecated).

-m 4 format:

Space-delimited summary of alignments containing 13 fields: qName tName score percentSimilarity qStrand qStart qEnd qLength tStrand tStart tEnd tLength mapQV

-m 5 format:

Space-delimited machine-parsable format containing 19 fields: qName qLength qStart qEnd qStrand tName tLength tStart tEnd tStrand score numMatch numMismatch numIns numDel mapQV qAlignedSeq matchPattern tAlignedSeq

**BLAST (alignment)**

Introduction: Basic Local Alignment Search Tool

Blastn searches a nucleotide query against a nucleotide database;

Blastp searches a protein query against a protein database;

Blastx search protein databases using a translated nucleotide query;

Tblastn search translated nucleotide databases using a protein query.

Input: reference sequence; query sequence in fasta format.

Output: blast results in txt format.

Test inputs:

blastn:

input_reference (fa/fas/fasta/fna)

imcas:/test_kira/blast/reference_nucl.fas

input_query (fa/fas/fasta/fna)

imcas:/test_kira/blast/query_nucl.fas

blastp:

input_reference (fa/fas/fasta/fna)

imcas:/test_kira/blast/reference_prot.fas

input_query (fa/fas/fasta/fna)

imcas:/test_kira/blast/query_prot.fas

blastx:

input_reference (fa/fas/fasta/fna)

imcas:/test_kira/blast/reference_prot.fas

input_query (fa/fas/fasta/fna)

imcas:/test_kira/blast/query_nucl.fas

tbastn:

input_reference (fa/fas/fasta/fna)

imcas:/test_kira/blast/reference_nucl.fas

input_query (fa/fas/fasta/fna)

imcas:/test_kira/blast/query_prot.fas

Test arguments:

args_threads (integer, [1])

args_evalue (float, [NULL])

args_dbtype (string, [NULL]) **nucl for blastn and tblastn, prot for blastp and blastx.**

Test Example:

blastn ?.txt 124.96 KB

blastp ?.txt 76.40 KB

blastx ?.txt 63.03 KB

tblastn ?.txt 116.82 KB

**AbundanceBin (community profiling)**

Introduction: AbundanceBin is an abundance-based tool for binning metagenomic sequences, such that the reads classified in a bin belong to species of identical or very similar abundances. AbundanceBin also gives estimations of species abundances and their genome size, these two important characteristic parameters for a microbial community.

Input: Sequencing reads file in fasta format.

Output: Results in tgz file (in tgz format) including

Test inputs (fasta/fna/fa/fas)

imcas:/test_kira/AbundanceBin/2_genome_50000.fasta

Test arguments:

args_bin_number (if the bin number is known, integer = 1~200, [NULL]) **2**

args_recursive_classification (using recursive method , flag, [FALSE])

args_exclude_max (exclude the kmers with counts > the number you set, integer = 0~100000, [NULL])

args_exclude_count (exclude the kmers with counts <= the number you set, integer = 0~10000, [NULL])

Test example:

?.tgz 592.82 KB

After uncompressed:

log file, several fasta files after bin (temp.1 temp.2), bin feature file, count file, unclassified of reads file.


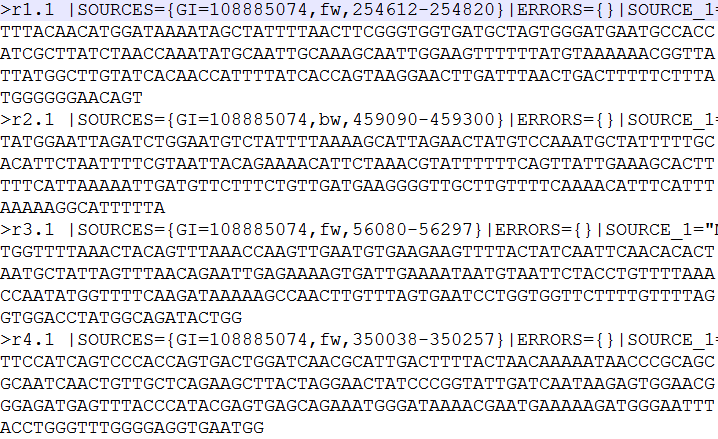


Figure: input fasta file


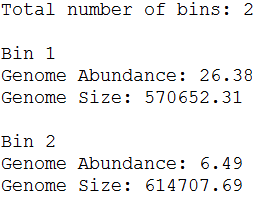

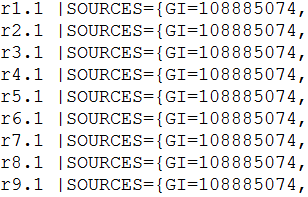

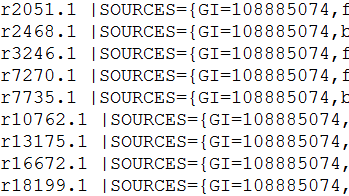


Figure: bining result and sequence id in two bin files

**BUSCO (validation with conserved gene)**

Introduction: BUSCO assessments are implemented in open-source software, with a large selection of lineage-specific sets of Benchmarking Universal Single-Copy Orthologs. These conserved orthologs are ideal candidates for large-scale phylogenomics studies, and the annotated BUSCO gene models built during genome assessments provide a comprehensive gene predictor training set for use as part of genome annotation pipelines.

Input: Genome assembly, Gene set (proteins), Transcriptome assessment in fasta format.

Output: BUSCO matches file.

Test inputs:

input_file (fasta/fa/fna/gz)

imcas:/test_kira/Lactobacillus.fasta

Test arguments:

args_evalue_blast (E-value cutoff for BLAST searches, float, [1e-03])

args_mode (Specify which BUSCO analysis mode to run, enum=prot|tran|[geno])

args_species (Name of existing Augustus species gene finding parameters, str, [null])

Test example:

?.tgz 12.63MB

Main results files:

short_summary_?.txt Contains a plain text summary of the results in BUSCO notation.Also gives a brief breakdown of the metrics.

full_table_?.tsv Contains the complete results in a tabular format with scores and lengths of BUSCO matches and coordinates (for genome mode) .

missing_buscos_list_?.tsv Contains a list of missing BUSCOs.

more information, please see: http://gitlab.com/ezlab/busco/raw/master/BUSCO _v3_userguide.pdf

**DFAST (annotation)**

Introduction: DFAST is a flexible and customizable pipeline for prokaryotic genome annotation as well as data submission to the INSDC. It is originally developed as the background engine for the DFAST web service and is also available as a stand-alone command-line tool.

Input: Genome in fasta format.

Output: Annatation files.

Test inputs:

input_fasta (fasta/fa)

/test_kira/simulate/Lactobacillus.fasta

Test arguments:

args_cpu (integer=1~32, [16])

Test example:

?.tgz 6.62MB

Unzip it:


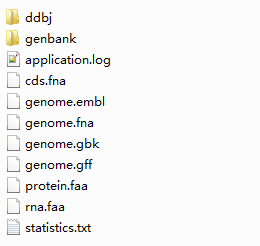


Figure: Results of DFAST


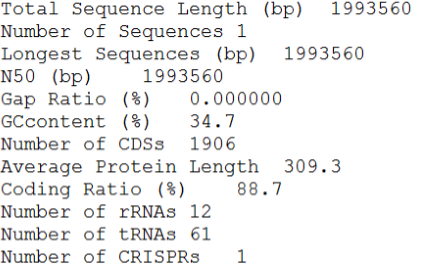


Figure: summary of DFAST

**DUDes (community profiling)**

Introduction: A top-down taxonomic profiler for metagenomics.

Input: Output file of bowtie2 in sam format.

Output: Taxonomic Profiling Output.

Test inputs:

input_sam (sam)

imcas:/test_kira/DUDes/mapping_output.sam

input_dudes_database (npz/bt2l)

imcas:/test_kira/DUDes_DB/dudesdb_arc-bac_refseq-cg_201709/arc-bac_refseq-cg_201709.npz

Test arguments:

args_threads (integer, default=1~32, [16])

Test example:

*.out 885.00B

The table contain 5 clounms, "@@TAXID RANK TAXPATH TAXPATHSN PERCENTAGE":


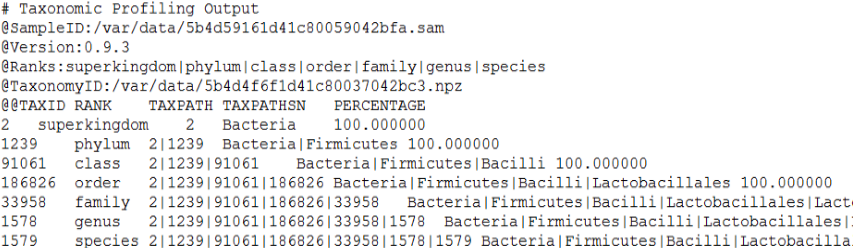


Figure: output file of DUDes

**Kaiju (community profiling)**

Introduction: Kaiju is a program for sensitive taxonomic classification of high-throughput sequencing reads from metagenomic whole genome sequencing or metatranscriptomics experiments.

Input: Input file containing reads both DNA or protein sequences in FASTA or FASTQ format, and a formatted database.

Output: Taxonomy results produced by Kaiju.

Test inputs:

input_fmi (fmi)

imcas:/test_kira/kaiju/kaiju_db.fmi

input_file1 & input_file2 (fasta/fa/fna/gz/fastq/fq)

imcas:/test_kira/mOTU/pirs_100_600_1.fq

imcas:/test_kira/mOTU/pirs_100_600_2.fq

input_nodes_dmp (dmp)

imcas:/test_kira/kaiju/nodes.dmp

Test arguments:

args_Minimum_Evalue (Minimum E-value in Greedy mode, float, [null] )

args_Minimum_match_length (Minimum match length, int=1~10000, [11])

args_Minimum_match_score_in_Greedy (Minimum match score in Greedy mode, int=1~100000, [65])

args_mode (Run mode, either mem or greedy enum=mem|[greedy])

args_Number_of_greedy_mismatches (Number of mismatches allowed in Greedy mode, int=1~10000, [3])

args_protein_seq (Ture if the input file is protein sequences, float, [FALSE])

args_threads (Number of parallel threads for classification, int=1~16, [16])

Test example:

*.tgz 317.51 MB

Unzip it, only a txt file is contained:


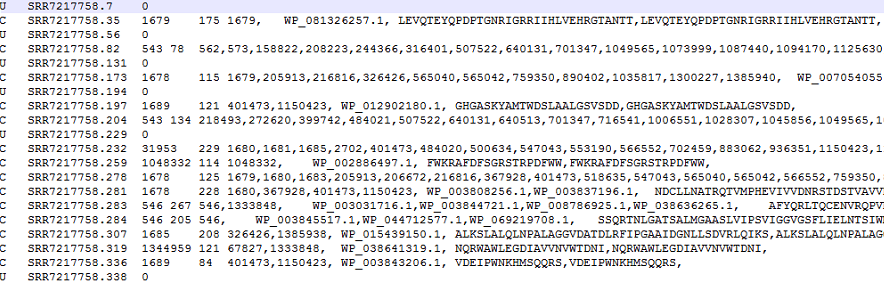


Figure: output of Kaiju

Column statement:

1. Either C or U, indicating whether the read is classified or unclassified.

2. name of the read

3. NCBI taxon identifier of the assigned taxon

4. the length or score of the best match used for classification

5. the taxon identifiers of all database sequences with the best match

6. the accession numbers of all database sequences with the best match

7. matching fragment sequence(s).

**mOTU (community profiling)**

Introduction: Phylogenetic marker genes are suitable to reconstruct the evolutionary history of organisms and to profile the taxonomic composition of environmental samples. For this purpose, a set of 40 protein-coding phylogenetic marker genes (MGs) have been identified . In the vast majority of known organisms, these 40 MGs occur in single copy and they have recently been used to delineate prokaryotic organisms at the species level. Due to these properties, they can be used to detect and accurately quantify not only known species, but also those that still lack genomic information. Based on a subset of these MGs that are suitable for shotgun sequencing data, we developed a method for taxonomic composition profiling of environmental samples.

Input: Paired-end reads in fastq format.

Output: Annotation file produced by mOTU.

Test inputs:

input_raw_reads_R1 & input_raw_reads_R2 (fastq/fq/gz)

imcas:/test_kira/mOTU/pirs_100_600_1.fq

imcas:/test_kira/mOTU/pirs_100_600_2.fq

Test example:

*.tgz 3.62MB

The most important results are saved in the RESULTS folder:

annotated.mOTU.abundances.gz, mOTU.abundances.gz,NCBI.species.abundances.gz


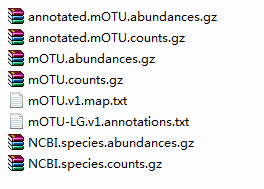


Figure: mOTU results

**RepeatMasker (repeat region detection)**

Introduction: RepeatMasker is a program that screens DNA sequences for interspersed repeats and low complexity DNA sequences. The output of the program is a detailed annotation of the repeats that are present in the query sequence as well as a modified version of the query sequence in which all the annotated repeats have been masked.

Input: DNA sequences in fasta format.

Output: Repeat region results.

Test inputs:

input_query (fasta/fa/fna)

imcas:/test_kira/virfinder/contigs.fa

Test arguments:

args_html (Creates an additional output file in xhtml format, flag, [TRUE])

args_gff (Creates an additional Gene Feature Finding format output, flag, [TRUE])

args_species (Specify the species or clade of the input sequence. The species name must be a valid NCBI Taxonomy Database species name and be contained in the RepeatMasker repeat database, string, **required**) **arabidopsis**

args_parallel (The number of processors to use in parallel, integer= 1~40, [NULL])

Test example:

?.tgz 20.66KB

Unzip it:

A file contains the submitted sequence(s) in which have been masked (fasta formate).

A table annotating the masked sequences.

A table summarizing the repeat content of the query sequence

Optionally file with alignments of the query with the matching repeats HTML file and GFF file.


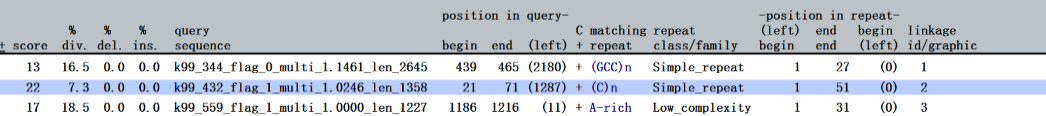


Figure: HTML file of RepeatMasker


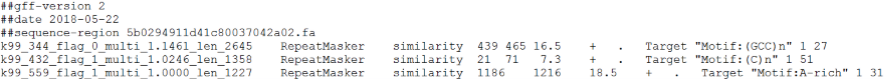


Figure: GFF file of RepeatMasker

**RNAmmer (RNA finding)**

Introduction: The RNAmmer 1.2 server predicts 5s/8s, 16s/18s, and 23s/28s ribosomal RNA in full genome sequences.

Input: Full genome sequences in fasta format.

Output: 5s/8s, 16s/18s, and 23s/28s ribosomal RNA position and sequence.

Test inputs:

input_reference (fasta/fa/fna)

imcas:/test_kira/Lactobacillus.fasta

Test arguments:

args_kingdom (Specifies the super kingdom of the input sequence, enum=euk|bac|[arc]) **bac**

args_multi (Runs all molecules and both strands in parallel, flag, [TRUE])

args_molecule_type (Molecule type can be 'tsu' for 5/8s rRNA, 'ssu' for 16/18s rRNA, 'lsu' for 23/28s rRNA or any combination seperated by comma, enum=lsu|tsu|ssu| [lsu,tsu,ssu])

Test example:

?.html 92.90 KB Hmmer search report

?.gff2 1.06 KB Position information

?.txt 16.02 KB Ribosomal RNA sequence

?.xml 68.99 KB Details about matching


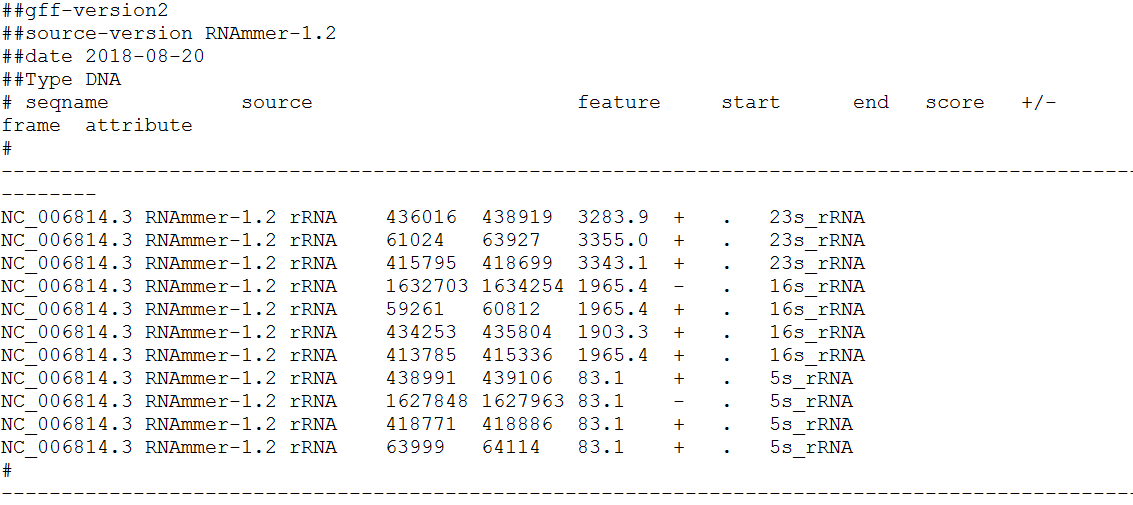


Figure: RNAmmer GFF file

**tRNAscan (tRNA prediction)**

Introduction: tRNAscan-SE identifies transfer RNA genes in genomic DNA or RNA sequences.

Input: FASTA sequences in fasta format.

Output: Transfer RNA genes results.

Test inputs:

input_reference (fasta/fa/fna)

imcas:test_kira/Lactobacillus.fasta

Test arguments:

args_bacterial_tRNAs (search for bacterial tRNAs, flag, [False])

args_archaeal_tRNAs (search for archaeal tRNAs, flag, [False])

args_organellar (search for organellar tRNAs, flag, [False])

args_general_tRNA (use general tRNA model cytoplasmic tRNAs from all 3 domains included, flag, [False])

Test example:

?.ss.txt 21.22 KB Secondary structure information

?.stats.txt 3.58 KB Statistic summary results

?.out.txt 3.17 KB Final tRNA results


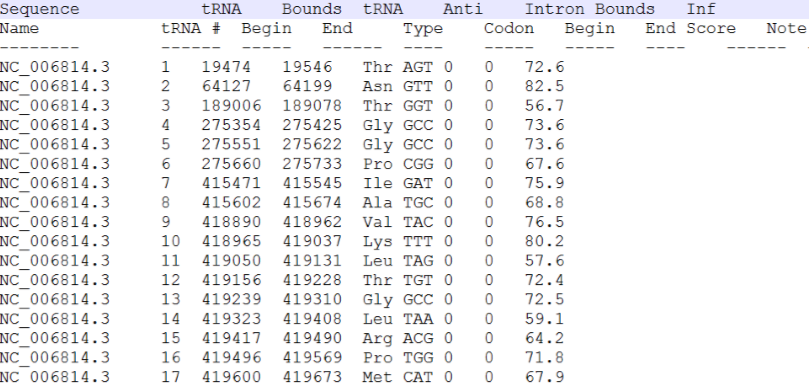


Figure: tRNAScan out.txt file

**Cuffdiff (differential expression)**

Introduction: Cuffdiff, an algorithm that estimates expression at transcript-level resolution and controls for variability evident across replicate libraries. Cuffdiff robustly identifies differentially expressed transcripts and genes and reveals differential splicing and promoter-preference changes.

Input: a gtf file generated by cufflinks，cuffcompare，cuffmerge or other software, reference sequence (in fasta format, Optional), SAM files for each samples.

Output: FPKM tracking files, Count tracking files, Read group tracking files, Differential expression test.

Test inputs:

input_gtf (gtf)

imcas:/test_kira/cuffdiff/transcripts.gtf

input_ref_seq (fasta/fa/fas)

imcas:/test_kira/cuffdiff/yeast.fas

input_sam (sam)

imcas:/test_kira/cuffdiff/s2_sorted.sam

imcas:/test_kira/cuffdiff/s1_sorted.sam

Test arguments:

args_L (comma-separated list of condition labels, array, default=NULL)

args_library_type (library prep used for input reads, enum=ff-firststrand|ff-secondstrand|[ff-unstranded]|fr-firststrand|fr-secondstrand|fr-unstranded|transfrags)

args_threads (number of threads used during quantification, integer, [1])

args_time_series (treat samples as a time-series, label, [False])

args_u (use “rescue method” for multi-reads, label, [False])

args_FDR (false discovery rate used in testing, float, [0.05])

Test example:

?.tgz 1.22MB

Unzip it, it contains the following files:

1. FPKM tracking files:

isoforms.fpkm_tracking: Transcript FPKMs

genes.fpkm_tracking: Gene FPKMs. Tracks the summed FPKM of transcripts sharing each gene_id

cds.fpkm_tracking: Coding sequence FPKMs. Tracks the summed FPKM of transcripts sharing each p_id, independent of tss_id

tss_groups.fpkm_tracking: Primary transcript FPKMs. Tracks the summed FPKM of transcripts sharing each tss_id

1. Count tracking files:

isoforms.count_tracking: Transcript counts

genes.count_tracking: Gene counts. Tracks the summed counts of transcripts sharing each gene_id

cds.count_tracking: Coding sequence counts. Tracks the summed counts of transcripts sharing each p_id, independent of tss_id

tss_groups.count_tracking: Primary transcript counts. Tracks the summed counts of transcripts sharing each tss_id

1. Read group tracking files:

isoforms.read_group_tracking: Transcript read group tracking

genes.read_group_tracking: Gene read group tracking. Tracks the summed expression and counts of transcripts sharing each gene_id in each replicate

cds.read_group_tracking: Coding sequence FPKMs. Tracks the summed expression and counts of transcripts sharing each p_id, independent of tss_id in each replicate

tss_groups.read_group_tracking: Primary transcript FPKMs. Tracks the summed expression and counts of transcripts sharing each tss_id in each replicate

1. Differential expression test:

isoform_exp.diff: Transcript differential FPKM.

gene_exp.diff: Gene differential FPKM. Tests differences in the summed FPKM of transcripts sharing each gene_id

tss_group_exp.diff: Primary transcript differential FPKM. Tests differences in the summed FPKM of transcripts sharing each tss_id

cds_exp.diff: Coding sequence differential FPKM. Tests differences in the summed FPKM of transcripts sharing each p_id independent of tss_id


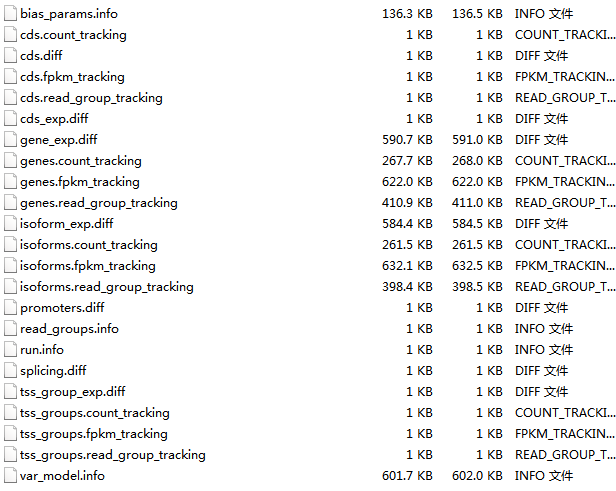


Figure: result of Cuffdiff


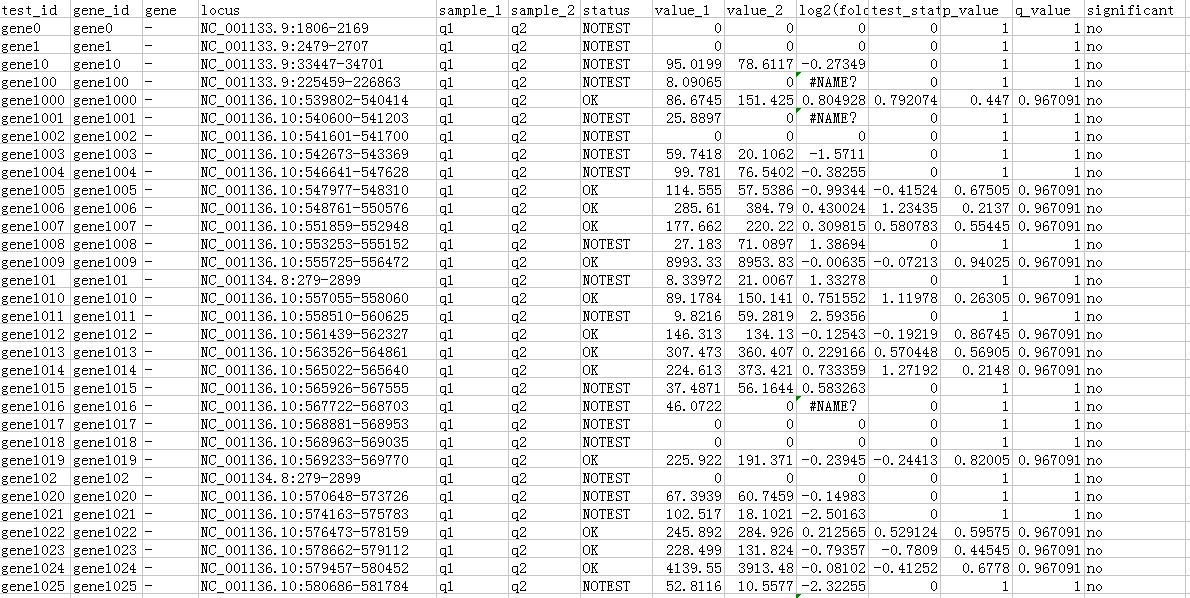


Figure: an example of *.diff file

Supplementary:

Cloumn 1: A unique identifier describing the transcipt, gene, primary transcript, or CDS being tested.

Cloumn 2: The gene_name(s) or gene_id(s) being tested.

Cloumm 3: Genomic coordinates for easy browsing to the genes or transcripts being tested.

Cloumn 4: Label (or number if no labels provided) of the first sample being tested.

Cloumn 5: Label (or number if no labels provided) of the second sample being tested

Cloumn 6: Can be one of OK (test successful), NOTEST (not enough alignments for testing), LOWDATA (too complex or shallowly sequenced), HIDATA (too many fragments in locus), or FAIL, when an ill-conditioned covariance matrix or other numerical exception prevents testing.

Cloumn 7: FPKM of the gene in sample x.

Cloumn 8: FPKM of the gene in sample y.

Cloumn 9: The (base 2) log of the fold change y/x

Column 10: The value of the test statistic used to compute significance of the observed change in FPKM.

Cloumn 11: The uncorrected p-value of the test statistic.

Cloumn 12: The FDR-adjusted p-value of the test statistic.

Cloumn 13: Can be either "yes" or "no", depending on whether p is greater then the FDR after Benjamini-Hochberg correction for multiple-testing.

**FLASH (merging)**

Introduction: FLASH (Fast Length Adjustment of SHort reads) is an accurate and fast tool to merge paired-end reads that were generated from DNA fragments whose lengths are shorter than twice the length of reads. Merged read pairs result in unpaired longer reads, which are generally more desired in genome assembly and genome analysis processes.

Input: pair-end reads in fastq format

Output: merged result.

Test inputs:

Input_seq1 (fastq/fq)

imcas:/test_kira/flash/subsample_r1.fq

Input_seq2 (fastq/fq)

imcas:/test_kira/flash/subsample_r2.fq

Test arguments:

Args_m (the minimum required overlap length between two reads to provide a confident overlap, integer, [10])

Args_M (maximum overlap length expected in approximately 90% of read pairs, integer, [65])

Test example:

?.tgz 672.9 KB

Unzip it, it contains the following files:

?.extendedFrags.fastq 3.8 MB The merged reads.

?.notCombined_1.fastq 8.5 KB Read 1 of mate pairs that were not merged

?.notCombined_2.fastq 8.5 KB Read 2 of mate pairs that were not merged

?.hist 1 KB Numeric histogram of merged read lengths.

?.histogram 1 KB Visual histogram of merged read lengths.

**HISAT2 (spliced mapping)**

Introduction: HISAT2 is a fast and sensitive alignment program for mapping next-generation sequencing reads (both DNA and RNA) to a population of human genomes (as well as to a single reference genome).

Input: sequencing reads in fastq format, reference sequence in fasta format and a list of known splice sites (optional)

Output: alignment result in SAM foumat

Test inputs:

input_reference (fa/fasta/fna/fas)

imcas:/test_kira/human_genome/hg19.fa

input_trim_seq1 (fastq/fq/gz)

imcas:/test_kira/RNA-pipeline/MBKD-4_1.fq.gz

input_trim_seq2 (fastq/fq/gz)

imcas:/test_kira/RNA-pipeline/MBKD-4_2.fq.gz

Test arguments:

args_CPU (integer, [2])

Test example:

?.sam 186.94 MB

**LoRDEC (error corrector for long reads)**

Introduction: LoRDEC is a program to correct sequencing errors in long reads from 3rd generation sequencing with high error rate, and is especially intended for PacBio reads. It uses a hybrid strategy, meaning that it uses two sets of reads: the reference read set, whose error rate is assumed to be small, and the PacBio read set, which is then corrected using the reference set. Typically, the reference set contains Illumina reads.

Input: long reads from 3rd generation sequencing in fasta or fastq format, short sequence with low error rate in fasta or fastq format.

Output: corrected long reads in fasta format.

Test inputs:

Input_reads (fasta/fastq/fq)

imcas:/test_kira/blasr/pacbio.fasta

Input_reference (fasta/fastq/fq/gz/zip)

imcas:/test_kira/blasr/SRR7624353.fasta

Test arguments:

args_k (the length of the k-mers that are counted and used in the graph, integer, [19])

args_s (solid k-mer abundance threshold. In other words a minimal number of occurrences of a k-mer such that it is assumed to be correct in Illumina reads, integer, [3])

Test example:

?.fasta 109.50MB

Supplementary:

**For bacterial species or eukaryotic species with small genomes, you may choose k=19 or 17, and s=2 or 3. For species with larger genomes, k=21 and s=2 or 3.**

**Oases (RNA *de novo* assembly)**

Introduction: Oases is a de novo transcriptome assembler designed to produce transcripts from short read sequencing technologies, such as Illumina, SOLiD, or 454 in the absence of any genomic assembly.

Input: Pair-end reads in fasta or fastq format.

Output: predicted transcript sequences and the file contig-ordering.txt explains the composition of these transcripts.

Test inputs:

Input_seq1 (fastq/fasta/fq/fa)

imcas:/test_kira/oases/reads2.left.fq

Input_seq2 (fastq/fasta/fq/fa)

imcas:/test_kira/oases/reads2.right.fq

Test arguments:

args_reads_type (enum = -short|[-shortPaired]})

args_reads_format (enum = fasta|[fastq])

args_k (the length of the k-mers being entered in the hash table, must be an odd number, integer, [27])

args_ins_len (expected distance between two paired-end reads in the second short-read dataset, integer, [200])

args_read_trkg (velvet’s read tracking can be turned on with the readtracking option, this will cost slightly more memory and calculation time, but will have the advantage of producing in the end a more detailed description of the assembly, enum = [yes]|no)

args_cov_cutoff (removal of low coverage nodes AFTER tour bus or allow the system to infer it, integer, [3])

args_edgeFractionCutoff (remove edges which represent less than that fraction of a nodes outgoing flow, float, [0.01])

args_min_pair_count (minimum length of output transcripts, integer, [5])

args_min_trans_lgth (the minimum transfrag length, integer, [100])

Test example:

?.tgz 45.74MB

Unzip it, it contains two files:

transcripts.fa (82KB): A FASTA file containing the transcripts imputed directly from trivial clusters of contigs.

**Proovread (error corrector for long reads)**

Introduction: proovread is a hybrid correction pipeline for single molecule real-time (SMRT) reads, which can be flexibly adapted on existing hardware and infrastructure from a laptop to a high-performance computing cluster.

Input: long reads from 3rd generation sequencing in fasta or fastq format, high coverage short read data with with small error rate in fasta or fastq format, unitigs can be used for correction in particular for large data sets (eukaryotes)(optional).

Output: trimmed and untrimmed reads.

Test inputs:

Input_long_reads (fasta/fastq/fq)

imcas:/test_kira/test_kira/blasr/pacbio.fasta

Input_short_reads (fasta/fastq/fq)

imcas:/test_kira/test_kira/blasr/SRR7624353.fasta

Test arguments:

args_coverage (stimated short read coverage, 50X recommended, integer, [50])

Test example:

?.tgz 34.22MB

Unzip it, it contains the following files:

?.trimmed.fa 150.5 KB

?.trimmed.fq 273.5 KB

high accuracy pacbio reads, trimmed for uncorrected/low quality regions.

?.untrimmed.fq 212.6 MB

complete corrected pacbio reads including un-/ poorly corrected regions.

?.chim.tsv 48.7 KB

annotations of potential chimeric joints clipped during trimming.

?.ignored.tsv 22.5 KB

ids of reads and the reason for excluding them from correction.

?.parameter.log 21.8 KB

the parameter set used for this run.

**REAPR (assembly validation)**

Introduction: REAPR is a tool that evaluates the accuracy of a genome assembly using mapped paired end reads, without the use of a reference genome for comparison. It can be used in any stage of an assembly pipeline to automatically break incorrect scaffolds and flag other errors in an assembly for manual inspection. It reports mis-assemblies and other warnings, and produces a new broken assembly based on the error calls.

Input: assembly result in FASTA format and the corresponding pair-end reads in fastq format.

Output: assembly error report, a new version of the assembly.

Test inputs:

input_assembly (fa/fas/fasta)

imcas:/test_kira/reapr/assembly.fasta

input_reads_1 (fastq/fq)

imcas:/test_kira/reapr/pirs_100_600_1.fq

input_reads_2 (fastq/fq)

imcas:/test_kira/reapr/pirs_100_600_2.fq

Test arguments:

NULL

Test example:

?.tgz 36.88 MB

Unzip it, it contains the following main files:

03.score.errors.gff.gz 2 KB a report of the errors found

04.break.broken_assembly.fa 1951 KB

a new version of the assembly, with scaffolds broken based on the errors found

05.summary.report.txt 1 KB

a summary of the errors found in the assembly, plus contiguity statistics (N50 etc) of the original and broken assemblies.

**Sailfish (transcript quantification)**

Introduction: Sailfish is a tool for transcript quantification from RNA-seq data. It requires a set of target transcripts (either from a reference or de-novo assembly) to quantify. All you need to run sailfish is a fasta file containing your reference transcripts and a (set of) fasta/fastq file(s) containing your reads. Sailfish runs in two phases; indexing and quantification. The indexing step is independent of the reads, and only needs to be run once for a particular set of reference transcripts and choice of k (the k-mer size). The quantification step, obviously, is specific to the set of RNA-seq reads and is thus run more frequently.

Input: the reference transcripts (in fasta format), Pair-end reads (in fastq format)

Output: quantification results (in sf format)

Test inputs:

input_trasncript (fa/fas/fasta)

imcas:/test_kira/sailfish/transcripts.fa

input_seq1 (fasta/fq)

imcas:/test_kira/sailfish/reads2.left.fq

input_seq2 (fasta/fq)

imcas:/test_kira/sailfish/reads2.right.fq

Test arguments:

args_kmer (must be an odd number, integer, [31])

args_library_type (format string describing the library type, enum = [IU]|ISF|ISR)

args_threads (the number of threads to use, integer, [4])

Test example:

?.sf 6.08KB


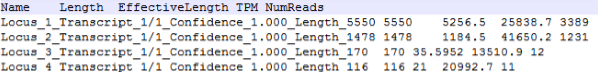


Figure: sailfish result

This file contains the result of the Sailfish quantification step. This file contains a number of columns (which are listed in the last of the header lines beginning with ‘#’). Specifically, the columns are (1) Transcript ID, (2) Transcript Length, (3) Transcripts per Million (TPM) and (6) Estimated number of reads (an estimate of the number of reads drawn from this transcript given the transcript’s relative abundance and length). The first two columns are self-explanatory, the next four are measures of transcript abundance and the final is a commonly used input for differential expression tools.

**SOAPdenovo-Trans (RNA *de novo* assembly)**

Introduction: SOAPdenovo-Trans is a de novo transcriptome assembler basing on the SOAPdenovo framework, adapt to alternative splicing and different expression level among transcripts.The assembler provides a more accurate, complete and faster way to construct the full-length transcript sets.

Input: pair-end reads (in fasta/fastq format)

Output: assembly contig sequence, assembly scaffold sequence.

Test inputs:

input_fq_seq1 (fastq/fq)

/test_kira/oases/reads2.left.fq

input_fq_seq2 (fastq/fq)

/test_kira/oases/reads2.right.fq

Test arguments:

Config_file arguments:

args_rd_len_cutof (The assembler will cut the reads from the current library to this length, integer, [100])

args_avg_ins (This value indicates the average insert size of this library or the peak value position in the insert size distribution figure, integer, [200])

args_reverse_seq (It tells the assembler if the read sequences need to be complementarily reversed. Illumima GA produces two types of paired-end libraries: a) forward-reverse, generated from fragmented DNA ends with typical insert size less than 500 bp; b) reverse-forward, generated from circularizing libraries with typical insert size greater than 2 Kb. The parameter “reverse_seq” should be set to indicate this: 0, forward-reverse; 1, reverse-forward, enum = [0]|1)

args_asm_flags (This indicator decides in which part(s) the reads are used. It takes value 1(only contig assembly), 2 (only scaffold assembly), 3(both contig and scaffold assembly), enum = 1|2|[3])

args_map_len (This takes effect in the “map” step and is the mininum alignment length between a read and a contig required for a reliable read location. The minimum length for paired-end reads and mate-pair reads is 32 and 35 respectively, enum = [32]| 35)

Other arguments:

args_K (kmer size., integer=23~127, [23])

args_M (mergeLevel (min 0, max 3): the strength of merging similar sequences during contiging, integer, [1])

args_L (minContigLen: shortest contig for scaffolding, integer, [100])

args_e (EdgeCovCutoff: edges with coverage no larger than EdgeCovCutoff will be deleted, integer=1~3), [2])

args_t (locusMaxOutput:output the number of transcripts no more than locusMaxOutput in one locus, integer, [5])

args_R (output assembly RPKM statistics,label, [False])

args_F (fill gaps in scaffolds, label, [False])

args_f (output gap related reads for SRkgf to fill gap, label, [False])

args_S (scaffold structure exists, label, [False])

Test example:

?.tgz 905.9KB

Unzip it, including the following main results:

?.contig 88KB

Contig information: corresponding edge index, length, kmer coverage, whether it's tip and the sequence. Either a contig or its reverse complementary counterpart is included. Each reverse complementary contig index is indicated in the *.ContigIndex file.

?.scafSeq 87KB Sequences of each scaffolds.

*.scaf: Contigs for each scaffold: contig index (concordant to index in *.contig), approximate start position on scaffold, orientation, contig length, and its links to others contigs.


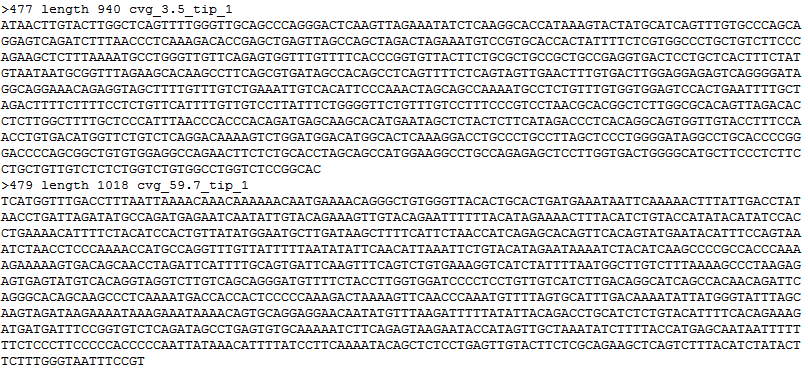


Figure: contig sequence

**TopHat (spliced mapping)**

Introduction: TopHat is a fast splice junction mapper for RNA-Seq reads. It aligns RNA-Seq reads to mammalian-sized genomes using the ultra high-throughput short read aligner Bowtie, and then analyzes the mapping results to identify splice junctions between exons.

Input: reference genome sequences (in fasta format), pair-end reads (in fastq format)

Output: a list of read alignments, a UCSC BED track of junctions, insertions and deletions

Test inputs:

input_reads_1 (fa/fas/fasta/fna)

imcas:/test_kira/cuffdiff/s1_y_1.fq

input_reads_2 (fa/fas/fasta/fna)

imcas:/test_kira/cuffdiff/s1_y_2.fq

input_reference (fa/fas/fasta/fna)

imcas:/test_kira/cuffdiff/yeast.fas

Test arguments:

args_threads (integer, default=NULL)

Test example:

?.tgz 12.45 MB

Unzip it, it contains the following main files:

accepted_hits.bam 11.9 MB

A list of read alignments in BAM format.

junctions.bed 15 KB

A UCSC BED track of junctions reported by TopHat. Each junction consists of two connected BED blocks, where each block is as long as the maximal overhang of any read spanning the junction. The score is the number of alignments spanning the junction.

insertions.bed 16 KB

A UCSC BED track of insertions reported by TopHat, chromLeft refers to the last genomic base before the insertion.

deletions.bed 20 KB

A UCSC BED track of deletions reported by TopHat, chromLeft refers to the first genomic base of the deletion.


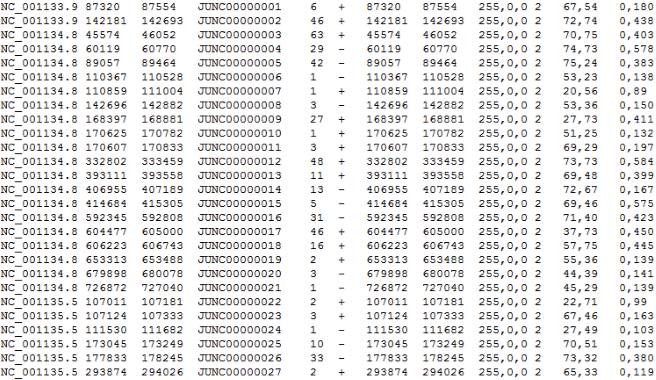


Figure: identified exon-exon splice junctions.

**Trim_Galore (quality control)**

Introduction: Trim Galore is a wrapper script to automate quality and adapter trimming as well as quality control, with some added functionality to remove biased methylation positions for RRBS sequence files (for directional, non-directional (or paired-end) sequencing).

Input: pair-end reads (in fastq format)

Output: trimmed reads, fastQC report

Test inputs:

input_seq1 (gz/tgz/fastq/fq)

imcas:/test_kira/RNA-pipeline/MBKD-4_1.fq.gz

Input_seq2 (gz/tgz/fastq/fq)

imcas:/test_kira/RNA-pipeline/MBKD-4_2.fq.gz

Test arguments:

args_e (maximum allowed error rate, float, [0.1])

args_length (discard reads that became shorter than length INT because of either quality or adapter trimming, integer, [20])

args_N (the total number of Ns (as integer) a read may contain before it will be removed altogether, integer, [1])

Test example:

?.tgz 37.51 MB

Unzip it, it contains the following files:

Trimmed reads file:

?_1.fq.gz 17.9 MB

?_2.fq.gz 18.4 MB

Fastqc report:

?_1_fastqc.html 638 KB

?_2_fastqc.html 635 KB

?_1_fastqc.zip 358 KB

?_2_fastqc.zip 360 KB

Trimming report:

?_trimming_report.txt 3KB

?_trimming_report.txt 3KB

**QIIME2 (metagenome 16s analysis pipeline)**

Introduction: QIIME 2 is a powerful, extensible, and decentralized microbiome analysis package with a focus on data and analysis transparency. QIIME 2 enables researchers to start an analysis with raw DNA sequence data and finish with publication-quality figures and statistical results.

Input: (1)16s RNA pair end sequence: paired-end demultiplexed fastq/fq/fastq.gz /fq.gz format OR 16s RNA single end sequence: multiplexed single-end fastq/fastq.gz format. (2) Metadata: sample metadata file in tsv format.

Output: Feature table produced by 16s RNA sequences. Tree files produced by phylogenetic diversity analyses. Alpha and beta diversity results file and Alpha rarefaction plotting. OTU file with taxonomy annotation.

Test inputs:

Input_directory(fq/fq.gz/fastq/fastq.gz)

imcas:/test_kira/QIIME2/single-end-sequence/*

Input_sample_metadata (tsv)

imcas:/test_kira/QIIME2/kinds-sample-metadata/Moving-Pictures-sample-metadata.tsv

Test arguments:

(QIIME2-1)

args_type_of_data (Which type data you want to import with qiime2, enum=single-end-sequence|[pair-end-sequence] ) **single-end-sequence**

arsg_p_n_threads (The number of threads to use for multithreaded processing. If 0 is provided, all available cores will be used, integer=0~40, [0] )

args_single_end_trunc_len (This truncates the 3' end of the of the input sequences, reads that are shorter than this value will be discarded, integer=0~500, [120], [required])

args_single_end_trim_left (Position at which sequences should be trimmed due to low quality. This trims the 5' end of the of the input sequences, which will be the bases that were sequenced in the first cycles, integer=0~500, [0])

arsg_pair_end_trunc_len_f (Position at which forward read sequences should be truncated due to decrease in quality, integer=0~500, [150], [required])

args_pair_end_trunc_len_r (Position at which reverse read sequences should be truncated due to decrease in quality, integer=0~500, [150], [required])

args_pair_end_trim_left_f (Position at which forward read sequences should be trimmed due to low quality, integer=0~500, [0])

args_pair_end_trim_left_r (Position at which reverse read sequences should be trimmed due to low quality. This trims the 5' end of the input sequences, which will be the bases that were sequenced in the first cycles, integer=0~500, [0])

(QIIME2-2)

args_alpha_rarefaction_plotting_max_depth (the maximum rarefaction depth, integer=0~1000000, [400])

args_beta_diversity_metadata_column (column from metadata file or artifact iewable as metadata, string, [NULL], [required]) **BodySite**

args_alpha_diversity_sampling_depth (the total frequency that each sample should be rarefied to prior to computing diversity metrics, integer=0~1000000, [1000], [required])

(QIIME2-3)

args_main_vector (sqlite where clause specifying sample metadata criteria that must be met to be included in the filtered feature table. If not provided, all samples in metadata that are also in the feature table will be retained, string, [NULL], [required]) **Bodysite**

args_value_of_main_vector (string, [NULL]) **gut**

args_ancom_metadata_cloumn (column from metadata file or artifact viewable as metadata. The categorical sample metadata column to test for differential abundance across, string, [NULL], [required]) **Subject**

args_ancom_level (the taxonomic level at which the features should be collapsed. All ouput features will have exactly this many levels of taxonomic annotation, integer=0~11, [6], [required])

Test example:

*.tgz 97.21MB QIIME2-1 output file

*.tgz 6.57MB QIIME2-2 output file

*.tgz 2.49MB QIIME2-3 output file


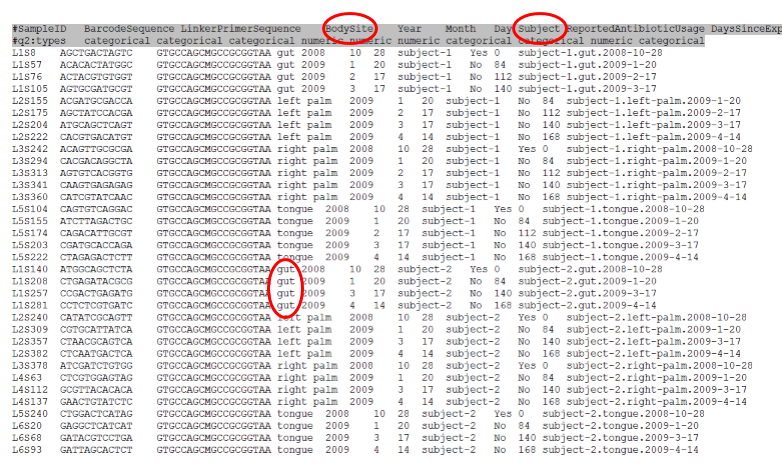


Figure: input metadata for QIIME2

The detailed requirement of metadata, please see https://docs.qiime2.org/2018.6/ tutorials/metadata/. The argument **BodySite**, **gut**, and **Subject** are all from metadata table.

QIIME2-1 output file contains:

emp-single-end-sequences.qza (file produced by importing data)

demux.qza (file produced by demultiplexing sequences)

demux.qzv (demux.qza visualizations)

denoising-stats.qza (file produced after Sequence quality control)

denoising-stats.qzv (denoising-stats.qza visualizations)

table.qza/rep-seqs.qza (FeatureTable and FeatureData summaries)

aligned-rep-seqs.qza/masked-aligned-rep-seqs.qza/unrooted-tree.qza/rooted-tree.qza (Generate a tree for phylogenetic diversity analyses)


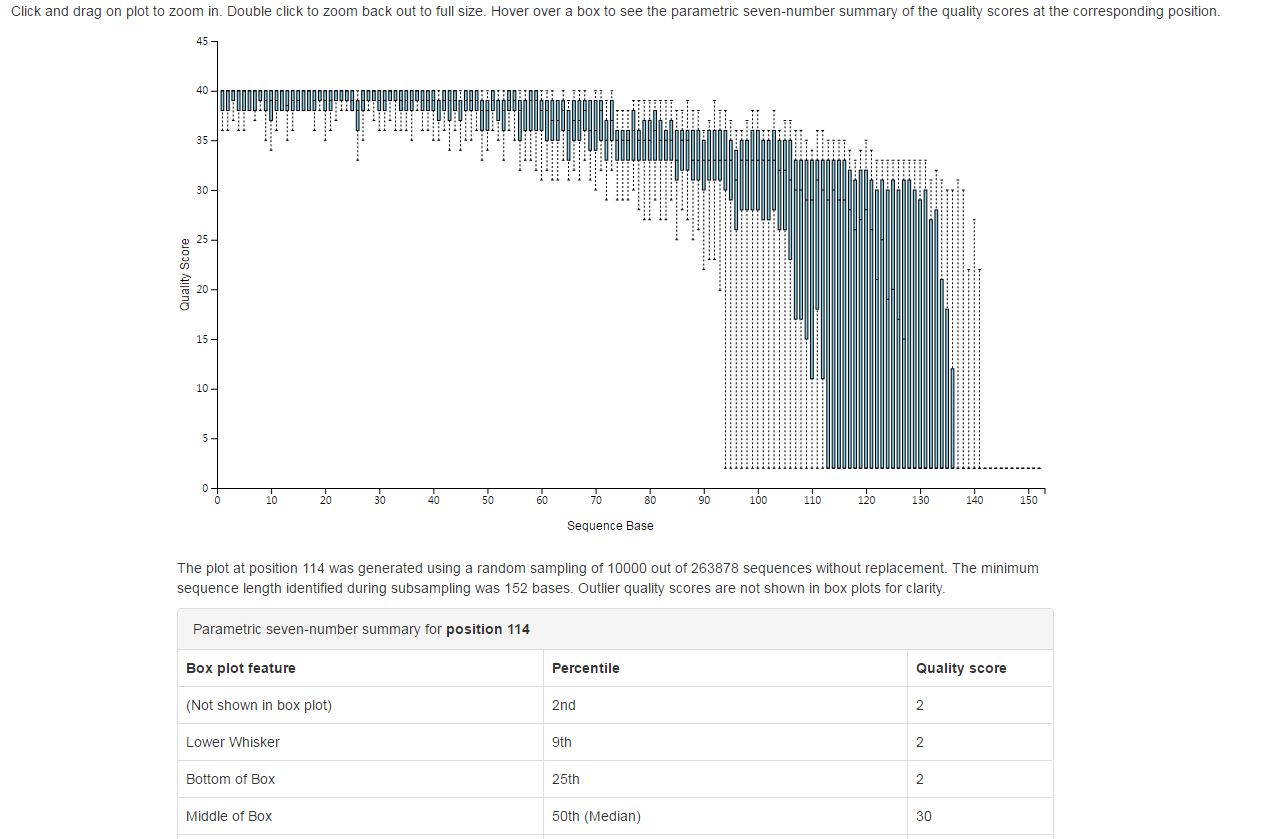


Figure1-2. demux.qzv


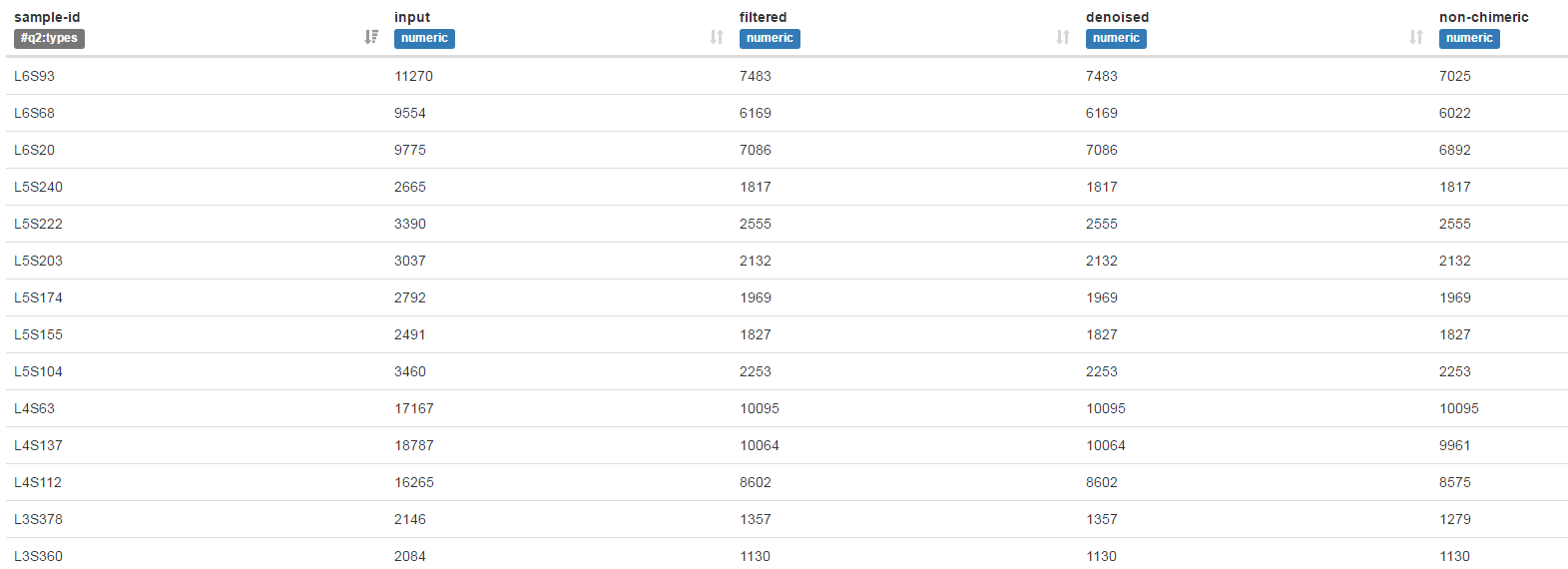


Figure1-3. denoising-stats.qzv

QIIME2-2 output file contains:

Alpha diversity:

Shannon_vector.qza (a quantitative measure of community richness)

Observed_otus_vector.qza (a qualitative measure of community richness)

Faith_pd_vector.qza (a qualitiative measure of community richness that incorporates phylogenetic relationships between the features)

Evenness_vector.qza ( a measure of community evenness);

Beta diversity:

Jaccard_distance_matrix.qza (a qualitative measure of community dissimilarity)

Bray_curtis_distance_matrix.qza (a quantitative measure of community dissimilarity)

Unweighted_unifrac_distance_matrix.qza/unweighted_unifrac_pcoa_results.qza (a qualitative measure of community dissimilarity that incorporates phylogenetic relationships between the features)

Weighted_unifrac_pcoa_results.qza/weighted_unifrac_distance_matrix.qza (a quantitative measure of community dissimilarity that incorporates phylogenetic relationships between the features)


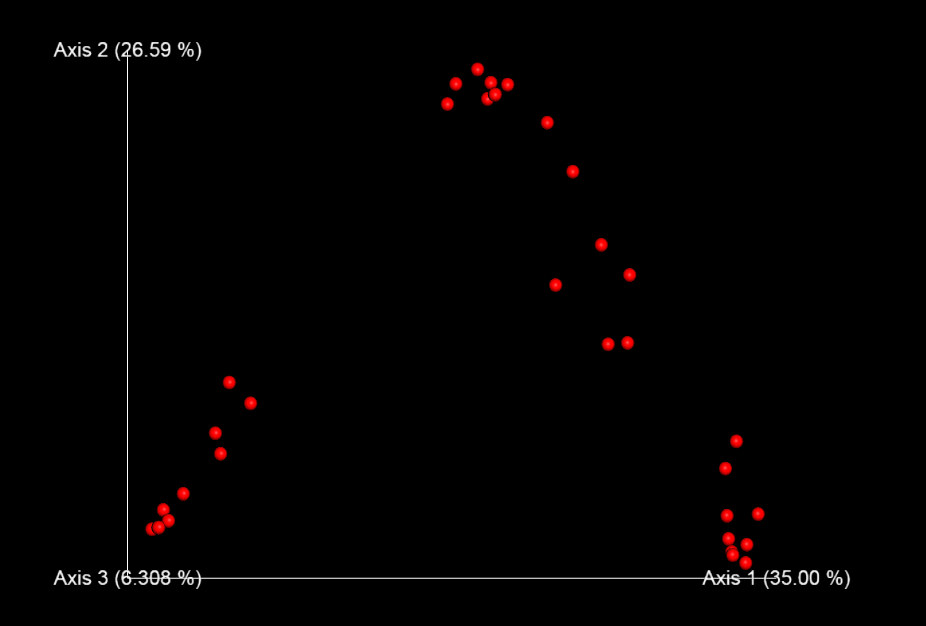

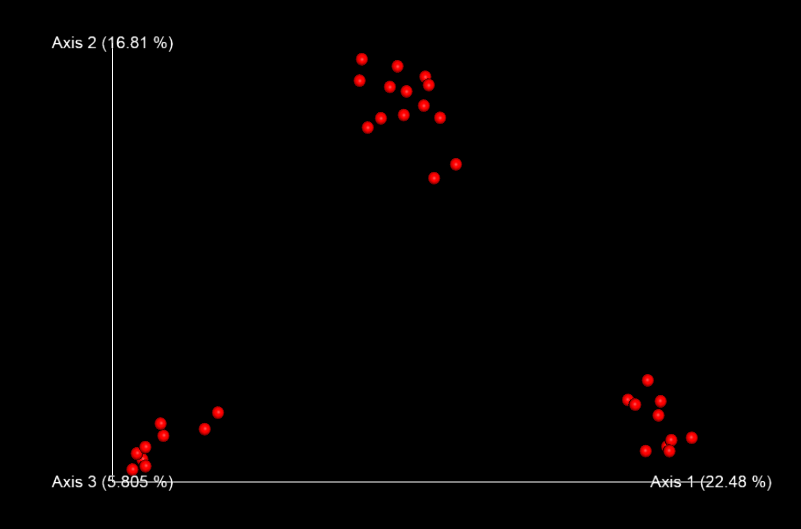


Figure: unweighted_unifrac_emperor.qzv Figure: jaccard_emperor.qzv


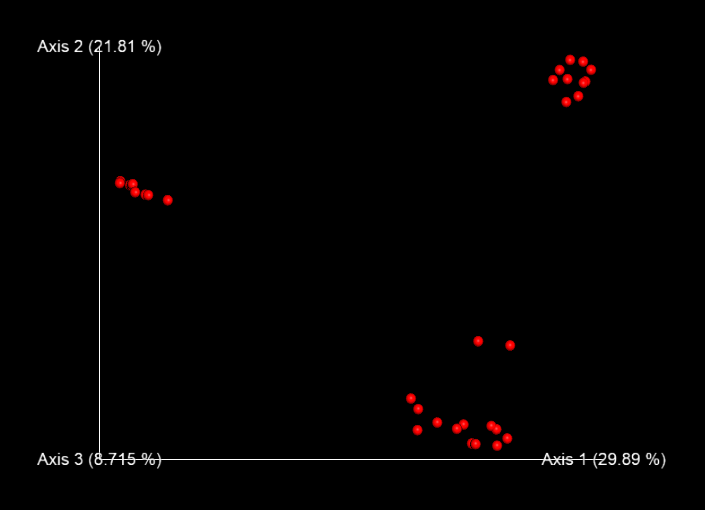

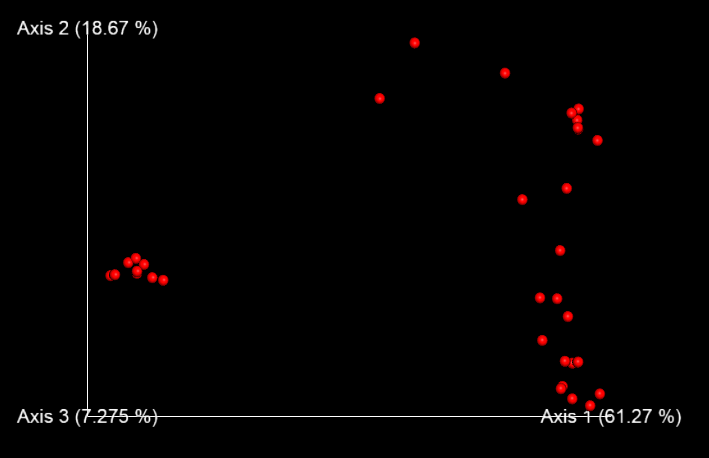


Figure: bray_curtis_emperor.qzv Figure: weighted_unifrac_emperor.qzv

QIIME2-3 output file:

Taxonomy.qza (Taxonomic analysis)

Gut-table.qza/comp-gut-table.qza/ gut-table-l6.qza /comp-gut-table-l6.qza (Differential abundance testing with ANCOM)


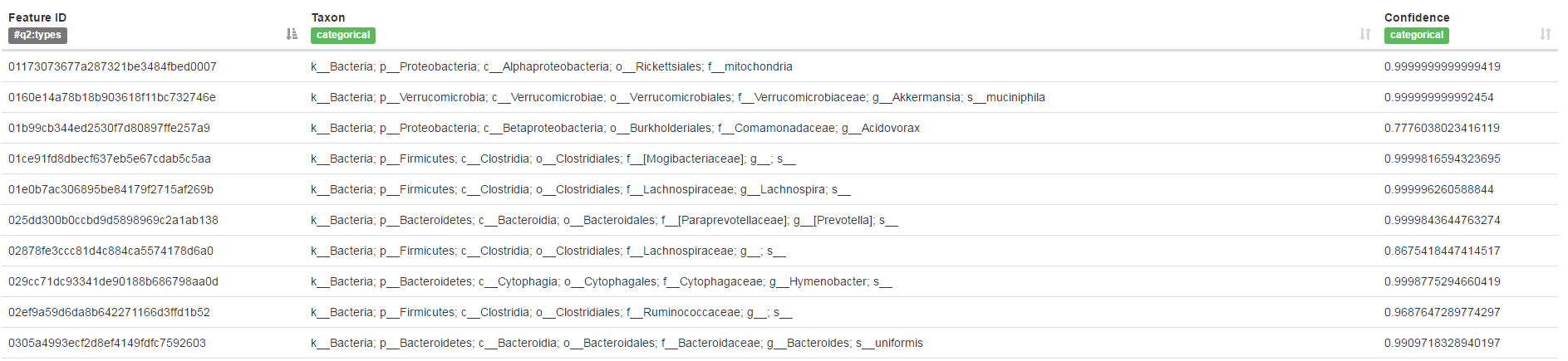


Figure: taxonomy.qzv


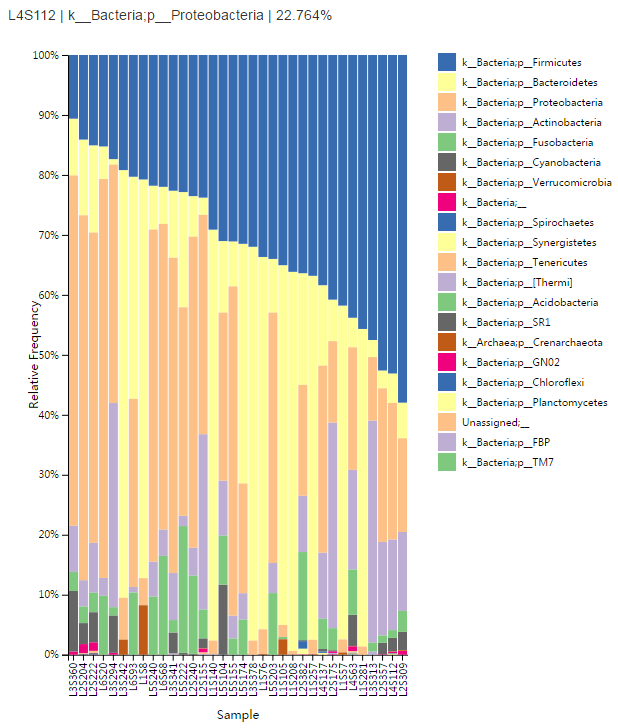


Figure: taxa-bar-plots.qzv

**Contamination removal (reads alignment)**

Introduction: Host contamination removal is an essential step for metagenomic analysis. This tools is used to remove contamination with reference genome alignment.

Input: Paired-end reads in fastq format and reference genome index.

Output: Filtered reads in fastq format.

Test inputs:

input_reference (bt2)

imcas:/test_kira/bowtie2_index/H_sapiens_hg19/ **(folder)**

input_seq1 (fastq/fq)

imcas:/test_kira/HUMAnN2/metagenome/ERR1474578_1.fastq

imcas:/test_kira/HUMAnN2/metagenome/ERR1474576_1.fastq

input_seq2 (fastq/fq)

imcas:/test_kira/HUMAnN2/metagenome/ERR1474578_2.fastq

imcas:/test_kira/HUMAnN2/metagenome/ERR1474576_2.fastq

Test arguments:

**(Presets)**

args_presets (enum=--very-fast|--fast|--sensitive|[--very-sensitive]|--very-fast-local|--fast-local|--sensitive-local|--very-sensitive-local)

**(Alignment)**

args_N (max mismatches in seed alignment, enum=[0]|1)

args_length (length of seed substrings, integer, default=3~32, [22])

args_i (interval between seed substrings w/r/t read len, string, [S,1,1.15])

args_n_ceil (func for max non-A/C/G/Ts permitted in aln, string, [L,0,0.15])

args_dpad (include <int> extra ref chars on sides of DP table, integer, [15])

args_gbar (disallow gaps within <int> nucs of read extremes, integer, [4])

args_ignore_quals (treat all quality values as 30 on Phred scale, flag, [FALSE])

args_nofw (do not align forward (original) version of read, flag, [FALSE])

args_norc (do not align reverse-complement version of read, flag, [FALSE])

args_no_1mm_upfront (do not allow 1 mismatch alignments before attempting to scan for the optimal seeded alignments, flag, [FALSE])

args_align_mode ( enum=[--end-to-end]|--local)

**(Scoring)**

args_ma (match bonus (0 for --end-to-end, 2 for --local), enum=[0]|2)

args_mp (max penalty for mismatch, integer, [6])

args_np (penalty for non-A/C/G/Ts in read/ref, integer, [1])

args_rdg (read gap open, extend penalties, string, [5,3])

args_rfg (reference gap open, extend penalties, string, [5,3])

**(Effort)**

args_D (give up extending after <int> failed extends in a row, integer, [15])

args_R (for reads w/ repetitive seeds, try <int> sets of seeds, integer, [2])

**(Performance)**

args_threads (integer, default=1~16, [NULL])

Test example:

removed_contaminate_seq1.tgz 45.13MB

removed_contaminate_seq2.tgz 44.76MB

Supplementary:

We provide several Bowtie2 index file in the folder imcas:/test_kira/bowtie2_index/(genus)_(species)_(addition_details).

For example:

H. sapiens (hg19), H. sapiens (GRCh38), R. norvegicus, M. maniculatus, B. taurus, A. thaliana and O. sativa.
